# Membrane Permeability in a Large Macrocyclic Peptide Driven by a Saddle-Shaped Conformation

**DOI:** 10.1021/jacs.3c10949

**Published:** 2024-02-08

**Authors:** Justin H. Faris, Emel Adaligil, Nataliya Popovych, Satoshi Ono, Mifune Takahashi, Huy Nguyen, Emile Plise, Jaru Taechalertpaisarn, Hsiau-Wei Lee, Michael F. T. Koehler, Christian N. Cunningham, R. Scott Lokey

**Affiliations:** † Department of Chemistry and Biochemistry, University of California, Santa Cruz, California 95064, United States; ‡ Department of Peptide Therapeutics, Genentech, South San Francisco, California 94080, United States; § Department of Early Discovery Biochemistry, Genentech, South San Francisco, California 94080, United States; ∥ Innovative Research Division, Mitsubishi Tanabe Pharma Corporation, Kanagawa 227-0033, Japan; ⊥ Department of Drug Metabolism and Pharmacokinetics, Genentech, South San Francisco, California 94080, United States; # Department of Analytical Research, Genentech, South San Francisco, California 94080, United States; ∇ Department of Medicinal Chemistry, Genentech, South San Francisco, California 94080, United States

## Abstract

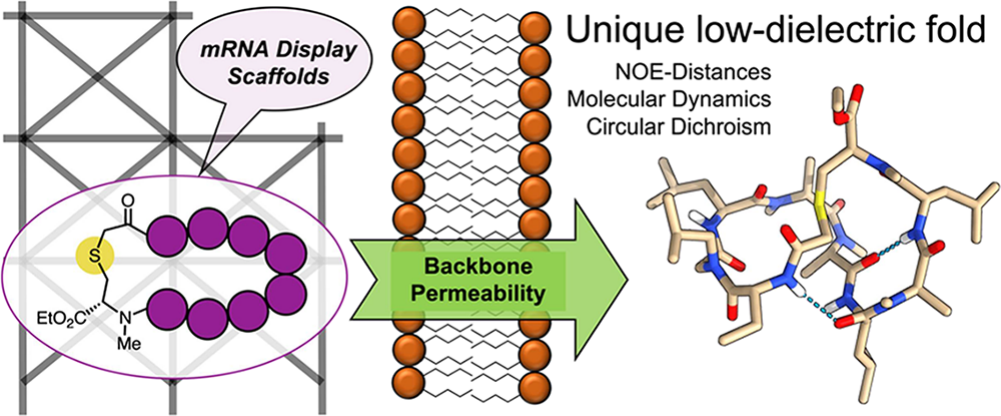

The effort to modulate challenging protein targets has stimulated interest in ligands that are larger and more complex than typical small-molecule drugs. While combinatorial techniques such as mRNA display routinely produce high-affinity macrocyclic peptides against classically undruggable targets, poor membrane permeability has limited their use toward primarily extracellular targets. Understanding the passive membrane permeability of macrocyclic peptides would, in principle, improve our ability to design libraries whose leads can be more readily optimized against intracellular targets. Here, we investigate the permeabilities of over 200 macrocyclic 10-mers using the thioether cyclization motif commonly found in mRNA display macrocycle libraries. We identified the optimal lipophilicity range for achieving permeability in thioether-cyclized 10-mer cyclic peptide-peptoid hybrid scaffolds and showed that permeability could be maintained upon extensive permutation in the backbone. In one case, changing a single amino acid from d-Pro to d-NMe-Ala, representing the loss of a single methylene group in the side chain, resulted in a highly permeable scaffold in which the low-dielectric conformation shifted from the canonical cross-beta geometry of the parent compounds into a novel saddle-shaped fold in which all four backbone NH groups were sequestered from the solvent. This work provides an example by which pre-existing physicochemical knowledge of a scaffold can benefit the design of macrocyclic peptide mRNA display libraries, pointing toward an approach for biasing libraries toward permeability by design. Moreover, the compounds described herein are a further demonstration that geometrically diverse, highly permeable scaffolds exist well beyond conventional drug-like chemical space.

## Introduction

Early-stage pharmaceutical discovery programs have begun to tackle challenging targets such as protein–protein interactions (PPIs), whose large, diffuse interfaces have made them notoriously difficult to drug with small molecules.^1,2^ Similar to biologics, macrocyclic peptides (MCPs) are larger and chemically more complex than typical small molecule drugs, allowing them to bind with a high affinity to these historically undruggable targets. However, unlike biologics, MCPs can, in principle, cross biological membranes, although identifying MCPs that are both biochemically functional and membrane permeable has remained a challenge due to the structural constraints imposed on permeability as molecules become larger. The modularity of MCPs allows for their large-scale diversification by way of combinatorial methods such as phage display,^3−5^ mRNA display,^6^ and DNA-encoded library technologies.^7^ Biasing the design of such libraries toward membrane-permeable scaffolds would thus significantly improve their application toward undruggable intracellular targets.^8,9^

**Figure 1:**
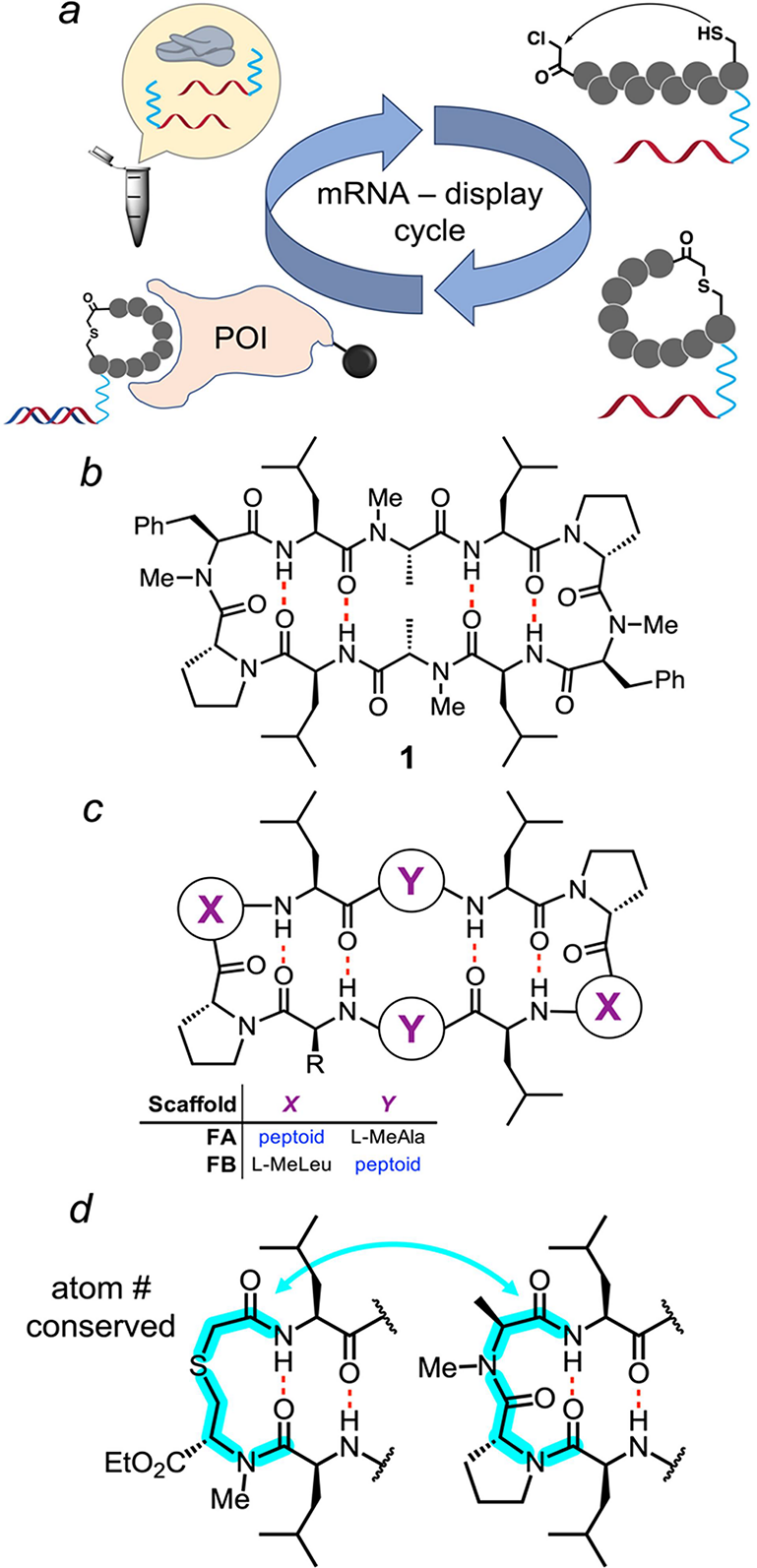
(a) General scheme describing the mRNA display process. POI = “protein of interest”. (b) Structure of original decapeptide (**1**) showing both permeability (PAMPA *P*_app_ = 5.01 × 10^–6^ cm/s, ALogP = 4.94) and oral bioavailability (46%*F* in rat) as reported by Fouché et al. (c) A general representation of the peptide/peptoid hybrid cyclic peptomer scaffolds (FA and FB) investigated by Furukawa et al. Side chain identity “*R*” is either Ala or Phe. (d) Comparison of the d-Pro-(NMe)Aaa β-turn mimetic with the mRNA display-type thioether linkage; emphasizing the conservation of backbone length.

In particular, mRNA display has enabled the discovery of high-affinity MCP leads against a variety of challenging targets.^10−12^ This technology utilizes an engineered in vitro ribosomal translation system to generate large libraries of MCPs in which each library member is covalently linked to its encoding nucleic acid strand. In one version of this chemistry, following the translation of the linear sequence, an S_N_2 reaction between a Cys side chain and an N-terminal chloroacetamide leads to the final cyclized product (Figure 1a). Highly diverse mRNA display libraries of up to 10^13^ unique MCPs can be synthesized, in which customization of the tRNA synthons^13−16^ allows incorporation of nonproteinogenic building blocks such as *N*-methyl^17^ and d-amino acids,^18^ peptoids,^19^ as well as β-,^20^ and γ-amino acids.^21,22^ Furthermore, postsynthetic tailoring reactions inspired by natural product synthases, such as enzymes involved in the biosynthesis of ribosomally synthesized and post-translationally modified peptides (RiPPs), enable additional diversity of mRNA display-derived scaffolds.^23,24^

The high-affinity leads derived from mRNA display libraries typically have high molecular weights (>1000 Da) and contain multiple polar and/or charged side chains.^25,26^ Optimization of druglike properties, including cell permeability, is often deferred to subsequent stages once initial hits are identified. Consequently, the number of leads emerging from screens against intracellular targets that also show potent activity in cells has been relatively modest, suggesting an alternative strategy in which permeability constraints are incorporated into the design of the initial libraries. The recent development of an mRNA display-derived lead MCP into a clinical KRAS inhibitor highlights the power of using drug-like elements such as backbone *N*-methylation to heavily bias the design of the initial library toward favorable ADME properties.^11^

Recent computational evaluation of backbone *N*-methylation variants of thioether MCPs in the 6-, 7-, and 8-mer size range have yielded highly permeable and orally bioavailable scaffolds relevant to mRNA display.^27^ Complementary to these computational approaches, we have previously reported an empirical strategy in which libraries of geometrically diverse, mass-encoded MCPs are synthesized and their permeabilities determined in a highly multiplexed assay format.^28−32^ Here we apply these tools to investigate the impact of backbone geometry on passive permeability in a series of 10-mer MCP thioethers (9 AAs with a linear thioether linker; ∼1000 Da size range) toward the discovery of a permeable scaffold that adopts a saddle-shaped, low-dielectric (i.e., membrane-associated) conformation. These findings not only underscore the diverse conformational landscape available to MCP scaffolds for achieving membrane permeability but also demonstrate the use of empirical methods for discovering drug-like MCP thioether scaffolds and their implications for the design of large, naïve mRNA display libraries with permeability as a guiding criterion.

## Results and Discussion

To determine the permeability landscape in large, thioether-derived MCPs, we started with the highly permeable cyclic decapeptide scaffold (**1**, Figure 1b) previously reported by Fouché, et al., which contains an extensive, low-dielectric intramolecular hydrogen bond (IMHB) network that sequesters all four of the backbone amide hydrogens in a cross-β conformation.^33,34^ Substitution of the *N*-methyl peptide residues in **1** with *N*-alkyl-Gly (peptoid) residues at either the turns (Figure 1c, “*X*”) or within the strands (Figure 1c, “*Y*”) yielded scaffolds with the same low-dielectric cross-β conformation as **1**, but with different degrees of solvent-dependent flexibility (i.e., “chameleonicity”) (Figure 1c).^35^ Since peptoids are derived synthetically from primary amines, the inclusion of peptoids in the design of permeable MCPs can enable extensive side chain diversification while removing the HBD of the secondary amide in canonical peptide space.^30,35,36^

**Figure 2:**
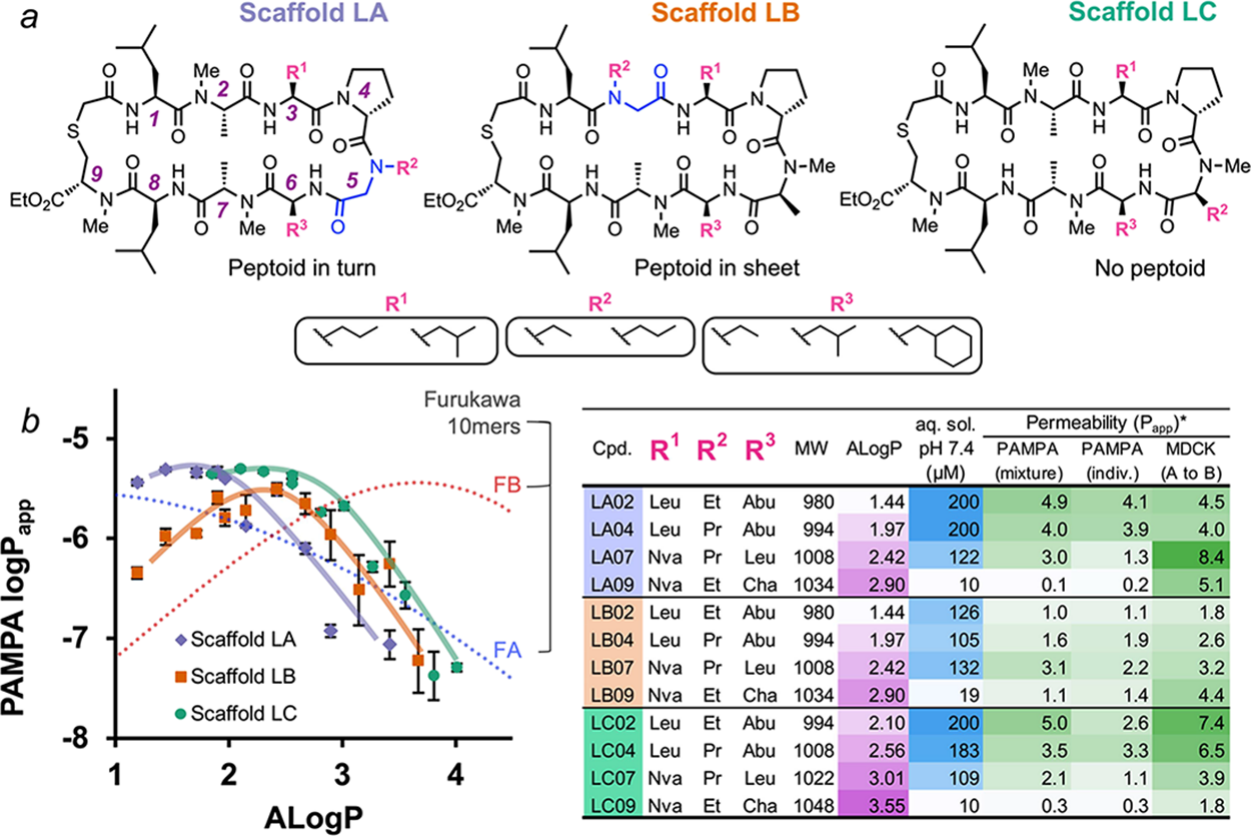
(a) Liposcan design broken down by scaffold-type. (b) Scatter plot of the liposcan compounds showing the relationship between ALogP and PAMPA permeability. Scaffold permeability trends of the cyclic decapeptomers investigated by Furukawa et al. (Figure 1c) are shown as dotted lines in blue (scaffold FA) and red (scaffold FB). Permeability values below the limit of detection are omitted in the plot. Resynthesized compound data are displayed in the table adjacent. *PAMPA and MDCK values are reported as *P*_app_ = (value) × 10^–6^ cm/s. See the Supporting Information for standard deviation values where applicable.

We noted that the d-Pro-(NMe)Ala dipeptide motif, which templates the two β-turns in **1**,^37^ contains the same number of atoms as the thioether linkage introduced by one of the more common cyclization strategies employed in mRNA display libraries (via an S_N_2 substitution from a Cys side chain onto an N-terminal haloacetamide), suggesting that substitution of one of the β-turn motifs in **1** (and similar MCP scaffolds^38^) with a thioether linkage may preserve the same cross-β IMHB network found in the parent scaffolds, thus preserving their favorable membrane permeability (Figure 1d). To test this hypothesis, we generated a series of atom-conserving thioether substitutions at one of the β-turns in three individual scaffolds based on **1**: scaffold LA, containing a peptoid residue in the turn, scaffold LB, containing a peptoid residue in the strand, and scaffold LC, containing no peptoid residues (Figure 2a). All amino acid residues were of L-stereochemistry except for the single, shared d-Pro (position 4) in the β-turn opposite the thioether.

**Scheme 1:**
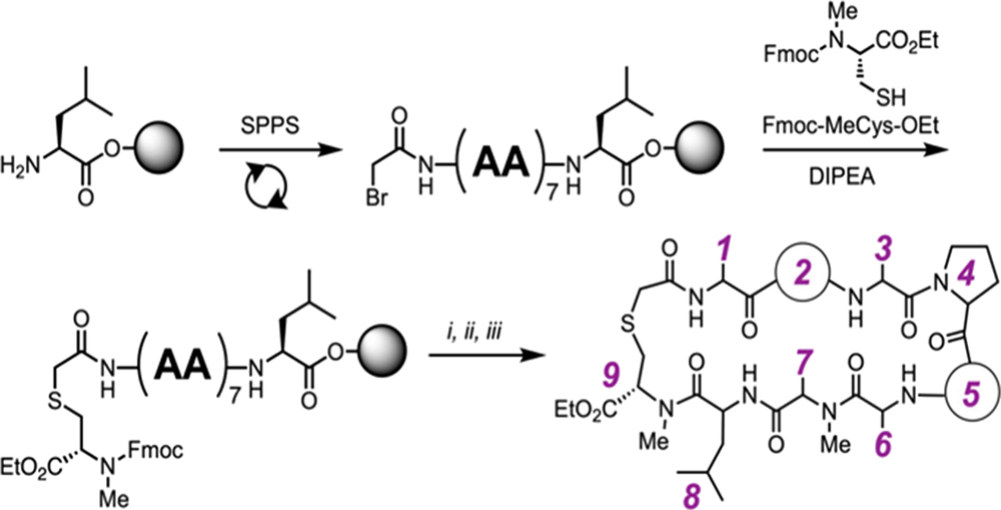
Synthesis of Thioether-Backbone Macrocyclic Peptides; Positions 2 and 5 May Assume the Identity of a Peptoid or Amino Acid Residue Depending on the Scaffold Type: (i) 20% Piperidine, DMF; (ii) 10% TFA, DCM; (iii) COMU, DIPEA, Acetonitrile (See the Supporting Information for Full Synthetic Conditions)

Passive permeability is a function of both membrane partitioning, which is often determined by the ability to sequester backbone NH groups in the membrane’s low dielectric through IMHB, and aqueous solubility, which decreases with increasing “bulk” lipophilicity as quantified by metrics such as the calculated octanol–water partition coefficient, ALogP.^39−41^ Thus, both membrane partitioning and aqueous solubility are governed by lipophilicity but in mutually opposing directions, leading to the often-observed inverted parabolic relationship between permeability and ALogP.^30,35,39,40,42^ Previous studies showed that for highly rigid scaffolds such as parent scaffold FA, the ALogP of maximum permeability (i.e., the *x*-axis displacement of the curve’s peak) occurs in the relatively polar (low) ALogP regime, while “chameleonic” scaffolds that display solvent-dependent conformational flexibility, such as scaffold FB and cyclosporine A, show peak permeabilities at higher lipophilicities (Figure 2b).^35^ Therefore, to determine the optimal ALogP for each thioether 10-mer scaffold, we performed a lipophilicity scan (“liposcan”) by generating a series of derivatives with different combinations of aliphatic side chains of varying length (*R*^1^–*R*^3^Figure 2a). The side chains at positions 1, 7, and 8 were held constant, while positions 3, 2, and 6 were varied among different aliphatic residues (*R*^1^, *R*^2^, and *R*^3^ respectively), generating 12 derivatives of each scaffold designed to span a broad lipophilicity range, between ALogP ∼1 and ∼4. The compounds were synthesized in a multiplex fashion using routine split-pool Fmoc-SPPS, incorporating Fmoc-MeCys-OEt as the final residue in the linear synthesis (Scheme 1). This route was chosen to both reduce racemization at the Cys side chain^43^ as well as to allow for synthetic automation of the linear precursor.

Passive permeabilities of the 36 compounds were measured in multiplex as mixtures of six compounds each using the parallel artificial membrane permeability assay (PAMPA).^44−46^ The permeabilities showed the expected inverted parabolic relationship between the permeability and ALogP (Figure 2b), with peak permeabilities for the three scaffolds occurring at different positions along the ALogP axis. The permeability for scaffold LA peaked at a relatively low ALogP of ∼1.7, while the permeabilities of scaffolds LB and LC peaked at higher ALogP values (2.1 and 2.5, respectively). In comparison to the parent scaffolds FA and FB, whose peak permeabilities were separated by more than two log units (Figure 2b, dotted lines), the peak permeabilities of the three thioether scaffolds spanned a narrower ALogP range (Figure 2b). Nonetheless, like their all-amide counterparts, placement of the peptoid residue in the β-turn (opposite the thioether) resulted in a peak permeability at a lower ALogP (scaffold LA), while the placement of the peptoid within the strand resulted in a peak permeability at a relatively higher ALogP (scaffold LB). These results are consistent with the increased backbone flexibility introduced by the thioether linkage relative to the d-Pro-(NMe)Aaa turn, leading to an increased chameleonicity overall relative to the parent scaffolds. These results were corroborated by multicanonical molecular dynamics (McMD) simulations of a representative member from scaffold LA and LB in solvents of increasing polarity (cyclohexane, chloroform, water, and DMSO).^47−49^ (Figures S1–S3). The simulations showed a more rigid backbone for LA and a greater solvent-dependent flexibility for LB, consistent with the destabilizing effect of peptoid placement in the strand vs the turn with respect to the cross β-sheet conformation in high-dielectric media observed for the all-amide parent scaffolds.^35^ These results were confirmed by amide temperature coefficient NMR experiments^50^ of two similar and permeable members of each scaffold (**LA04**, **LB07**, Figure S4); revealing a single major conformation for **LA04** in both CDCl_3_ and DMSO-d_6_ while **LB07** demonstrated multiple conformations in DMSO-d_6_ yet only a single conformer in CDCl_3_.

To confirm the permeabilities of the liposcan libraries measured in multiplex for scaffolds LA, LB, and LC (Figure 2, Table S1), we selected four compounds within each scaffold for resynthesis and tested their PAMPA permeabilities as pure compounds. The PAMPA permeabilities of the pure compounds showed an excellent correlation (Figure S5, *R*^2^ = 0.90) with their permeabilities determined in the original mixtures, consistent with previous reports comparing multiplexed permeabilities with those obtained from pure compounds.^29,31,51−53^ We also tested the permeabilities of the pure compounds in a cell-based trans-well permeability assay using Mdr1 knockout Madin–Darby canine kidney (MDCK) cells which minimize the endogenous efflux activity of traditional MDCK cells.^54^ The correlation between the PAMPA and MDCK assays was fair (Figure S6, *R*^2^ = 0.53), with permeabilities in MDCK cells being higher overall, especially for the higher-ALogP compounds. These observations are consistent with previous studies in cyclic peptides^39,40^ showing a higher penalty for more lipophilic compounds in PAMPA compared to cell-based permeability assays. Not surprisingly, the aqueous solubilities of all three scaffolds decreased as a function of increasing ALogP, although the solubility trends did not exactly match the predictions based on the shapes and *x*-axis displacements of the ALogP-vs-permeability curves. For example, at ALogP = 1.44, **LA02** was more soluble than LB02, despite the prediction that the representative of the more chameleonic scaffold LB would be more soluble at the more polar end of the continuum. Nonetheless, taken together, these results confirm that the thioether-linked versions of the Fouché 10-mer and its peptoid congeners maintain their high passive permeability over a wide lipophilicity range, suggesting that extensive side chain variation is possible while maintaining the desired low-dielectric conformation of the parent scaffolds.

**Figure 3:**
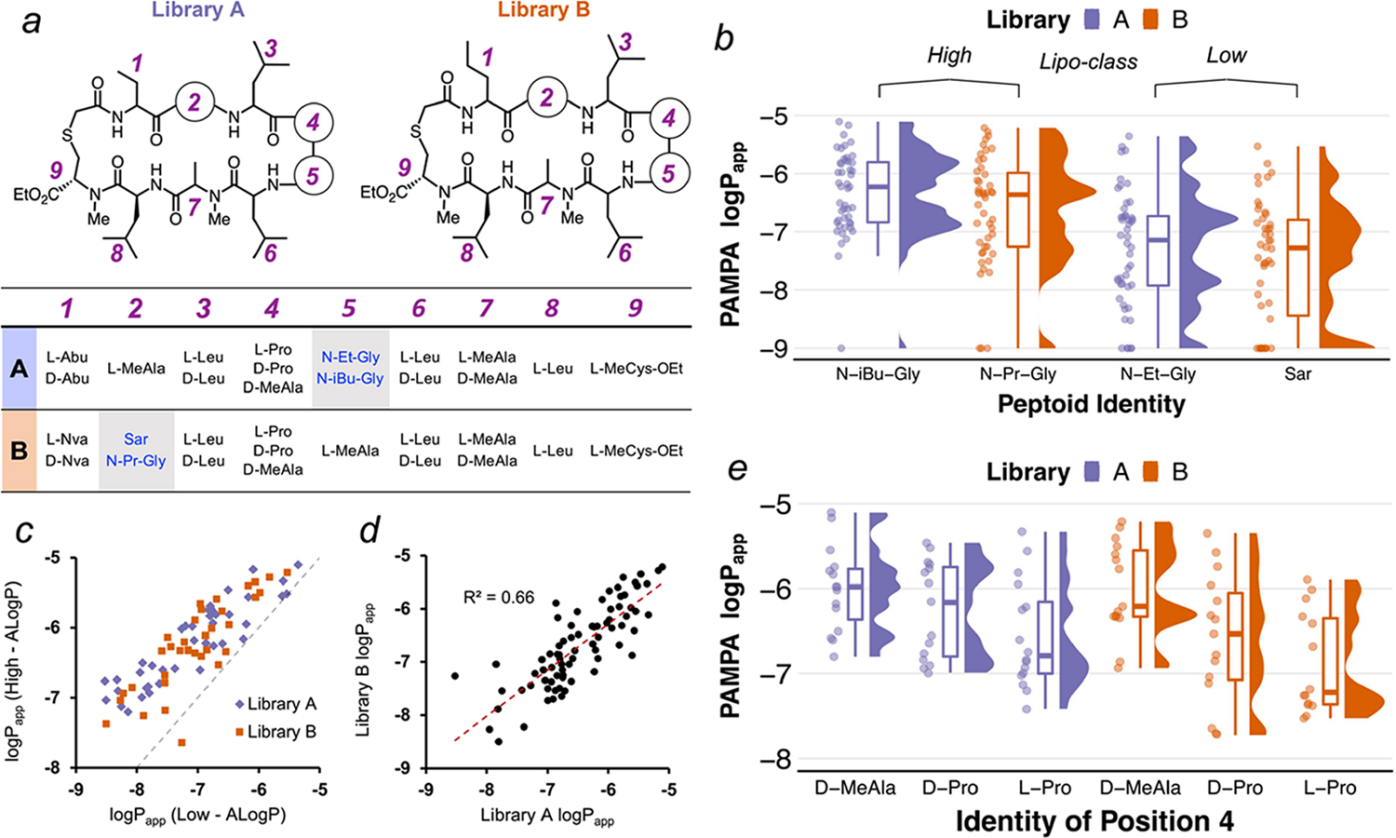
(a) Stereoscan library design broken down by scaffold type. Boxes shaded in gray denote the peptoid position. (b) Scatter, box, and density plots of PAMPA permeability versus peptoid identity. (c) Scatter plot showing the correlation of permeability values between matched pairs of compounds within the lower and higher lipophilicity classes. The dashed line in gray represents unity. (d) Scatter plot showing the correlation between matched pairs of compounds differing only in peptoid position. (e) Scatter, box, and density plots of the PAMPA permeability of library compounds only within the higher lipophilicity class shown as a function of the identity of position 4. Compounds below the limit of detection were omitted in all plots c–e.

For relatively small and/or rigid macrocycles, backbone stereochemistry is known to have a significant impact on MCP permeability, either by stabilizing lipophilic conformations through IMHB formation or by sterically shielding polar groups in the membrane.^29,31,55−62^ In contrast, there have been relatively few studies on the effect of stereochemistry on permeability in larger, more flexible scaffolds, such as the thioethers in the present study. Therefore, we performed a stereochemical scan (“stereoscan”) on scaffolds LA and LB to generate new libraries, A and B, respectively (Figure 3a). For synthetic efficiency, the chirality at l-Leu8 and l-Cys9 was held constant, while stereochemistry was permuted at positions 1, 3, 6, and 7 (Figure S7).

Based on the differences observed in the ALogP values at which the 10-mer scaffolds (i.e., LA, LB, LC, FA, and FB) (Figure 2b) achieved maximum permeability, we hypothesized that varying backbone stereochemistry among thioethers LA and LB would similarly give rise to different permeability maxima among the stereoisomers. Thus, we generated two ALogP variants for each stereochemical scaffold by varying the lipophilicity at the peptoid position, producing two lipophilicity classes for each library, at ALogP ∼1.4 and ∼2.3. In addition, due to its importance in templating one of the β-turns found in the low dielectric conformations of the parent scaffolds, we varied the geometry at the d-Pro residue of position 4, replacing it with either l-Pro or d-MeAla, thus allowing possible access to alternative low dielectric conformations. In total, the PAMPA permeabilities of 96 thioether MCP scaffolds were investigated, in which stereochemistry, peptoid position, and rigidity at position 4, were varied, with each scaffold being represented by two ALogP variants (Figure S7) based on the length of the *R*-group at the peptoid position.

Although the permeabilities of these scaffolds spanned at least 4 log units, nearly one-third of them (31/96) had permeabilities above 1 × 10^–6^ cm/s, while three-quarters (73/96) had permeabilities over 0.1 × 10^–6^ cm/s. For most of the matched pairs containing the same backbone geometry but different ALogP values, the correlation between ALogP and permeability was positive, indicating that most of these compounds fall on the left, positive sloping portion of the ALogP-permeability curve (Figures 3c, and S8). The permeability differences between the peptoid positional variants, that is, the matched pairs between libraries A and B, were similar across the scaffolds, indicating that other backbone features such as relative stereochemistry and the nature of the turn-promoting residue at position 4 have a greater impact on permeability than peptoid position in these scaffolds (Figure 3d, and S9). While compounds with an l-Pro at position 4 were, on the whole, less permeable than their d-Pro or d-MeAla counterparts, l-Pro4 was particularly detrimental to permeability for library B, indicating that the peptoid position can cooperate with other backbone elements to have a significant impact on permeability (Figure 3e).

**Figure 4:**
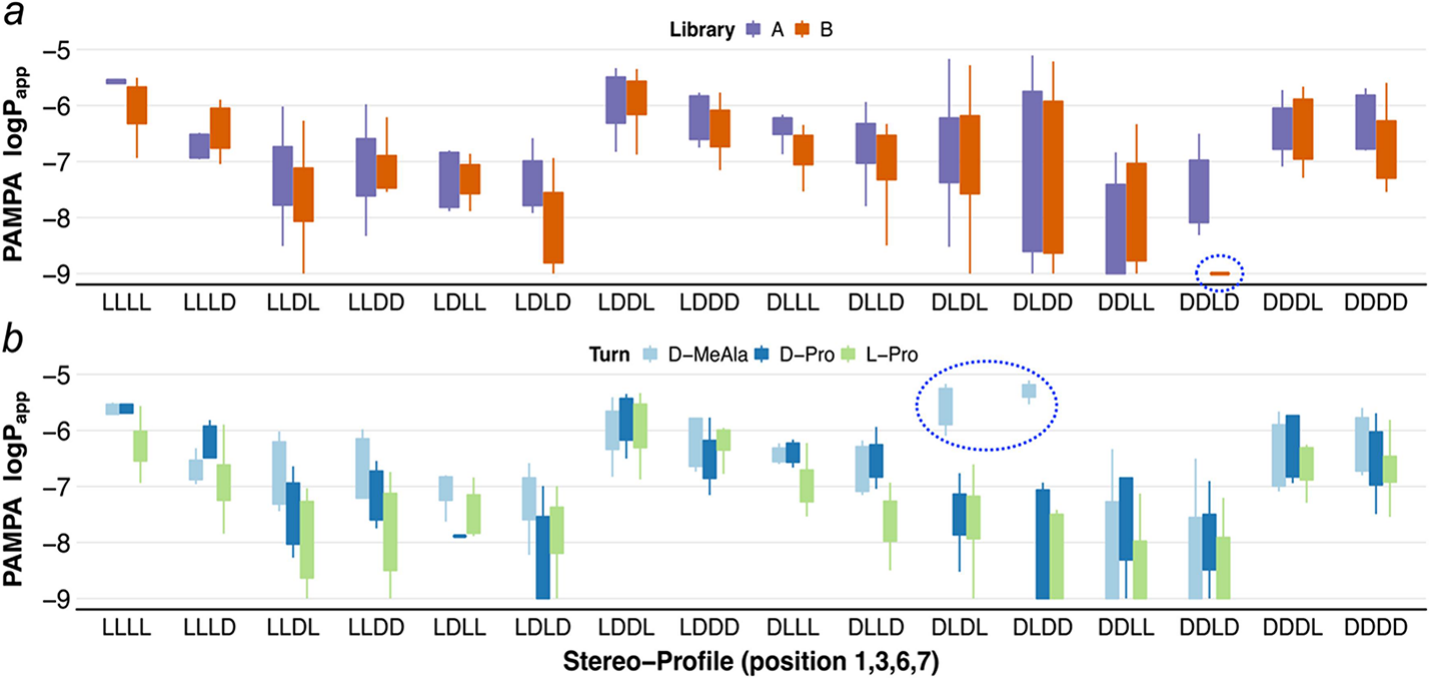
Box plots displaying the PAMPA permeability of each stereochemical combination at positions 1, 3, 6, and 7, respectively (termed “stereo-profile”). (a) Analyzed by scaffold-type; Library A (purple) and Library B (orange). *N* = 6 for each box. (b) Analyzed by the identity of the turn-residue at position 4; d-MeAla (light-blue), d-Pro (dark-blue), and l-Pro (green). *N* = 4 for each box, and significant anomalies are circled with a blue dashed line.

Analyzing permeability as a function of stereochemistry at positions 1, 3, 6, and 7 revealed that certain diastereomers were particularly favorable for permeability, while other stereochemical patterns were unfavorable. For most stereoisomers, libraries A and B performed similarly, except for those with the DDLD arrangement (corresponding to stereochemistry at residues 1, 3, 6, and 7, respectively), which showed much lower permeability for Library B compared with Library A (Figure 4a). Permeability trends were also generally conserved among the different turn-promoting residues, again highlighting the overall detrimental effect of l-Pro compared with d-Pro and d-MeAla (Figures 4b, and 3e). While the parent stereochemistry (LLLL) ranked highly among the other diastereomers, the DLDD and DLDL stereochemical groups demonstrated the largest range of permeabilities and contained the highest permeating members. Interestingly, the highest permeating compounds share a d-MeAla at position 4 (Figure 4b, blue dotted oval) and performed far better than the other compounds with this stereochemistry that contained d- or l-Pro at position 4. The unusually strong preference for d-MeAla at this position for only these two closely related stereochemical groups suggested that these compounds may exhibit a low-dielectric conformation that is distinct from the classic cross-β conformation of the parent compounds.

**Figure 5:**
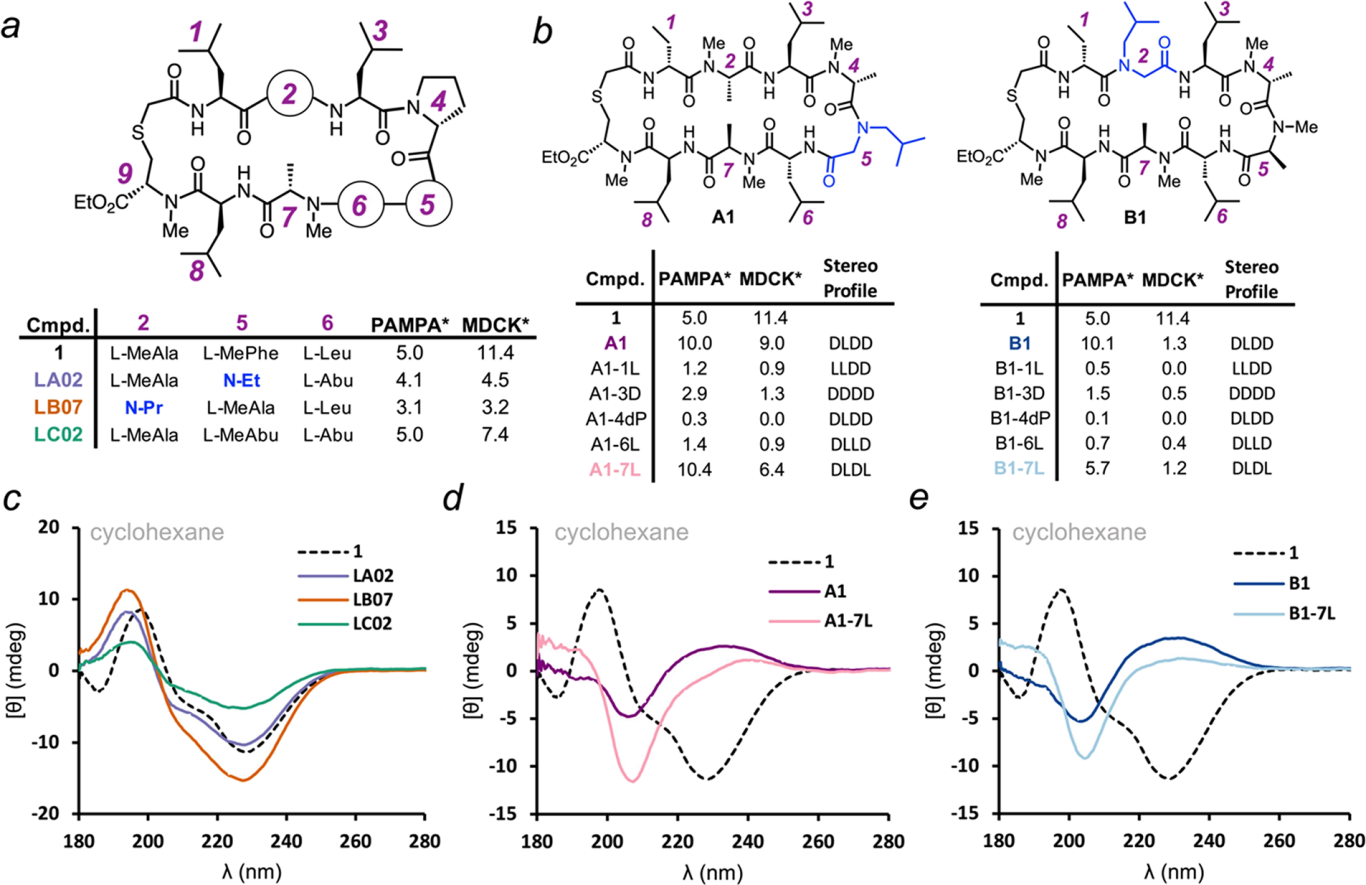
(a) Structural representation of highest permeating liposcan scaffold members [compounds **LA02, LB07**, and **LC02**] and decapeptide **1**. Side chain identities and measured permeability values (PAMPA and MDCK) are shown below. Peptoid residues are highlighted in blue. (b) Structures of the highest permeating stereoscan scaffold members: **A1** and **B1**. Measured permeability values (PAMPA and MDCK) and stereoprofile of each compound are shown below. (c) CD spectra of liposcan compounds **LA02** (light purple), **LB07** (orange), **LC02** (green)], and decapeptide **1** (black, dashed). The general shape of the CD-spectra demonstrated by all three liposcan compounds is like that of decapeptide **1** suggesting a shared cross-β conformation. (d) CD spectra of stereoscan compounds: **A1** (purple), **A1–7L** (light-pink) and decapeptide **1** (black, dashed). (e) CD spectra of stereoscan compounds: **B1** (navy), **B1–7L** (light-blue) and decapeptide **1** (black, dashed). All CD spectra were acquired in triplicate for each peptide at 50 μM in cyclohexane, displayed as the average of the three runs. *PAMPA and MDCK values are reported as *P*_app_ = (value) × 10^–6^ cm/s.

To better understand the permeability bias exhibited by the DLDD and DLDL stereochemical groups, we individually resynthesized the top permeating compound in each library (A1 and B1; Figure 5b) and each of the single-residue stereoisomers (varying only one of the following positions: 1, 3, 6, 7) for each of those compounds. Additionally, we synthesized the d-Pro variant of each compound to confirm that the bias was truly dependent upon having a d-MeAla at position 4 (Table S2). Taken together, the PAMPA permeabilities of the individually synthesized compounds corroborated the library cassette analysis (*R*^2^ = 0.92, Figures S10 and 5b) and the MDCK cell-permeability rates among the stereoisomers demonstrate the same trend (*R*^2^ = 0.82, Figure S11).

To understand the conformational aspects of the scaffolds investigated thus far, we first sought to validate whether the thioether liposcan compounds adopt the same cross-β conformation as seen in the original Fouché scaffold, in which the overall fold is defined by two opposing β-turns and a cross-β network of four transannular hydrogen bonds between residues 1 and 8 and between residues 3 and 6. Circular dichroism (CD) spectroscopy represents one such structural method that can be used to identify secondary structures in peptides.^63−65^ We hypothesized that since the liposcan thioether scaffolds are very similar to decapeptide **1**, a CD spectrum similar in shape to that of decapeptide **1** would be indicative of a shared cross-β conformation. All CD measurements were performed in cyclohexane to mimic the cell membrane’s low-dielectric environment, and we used the Fouché decapeptide **1** as the cross-β standard (Figure 5c–e). Indeed, the CD spectra of the top-permeating compound from each liposcan scaffold, **LA02**, **LB07**, and **LC02** (Figure 5a) were similar to that of parent decapeptide **1**, with similar minima and maxima near 230 and 190 nm, respectively (Figure 5c). Extensive NMR, X-ray, and computational evidence exist in support of the canonical cross-β low-dielectric conformation for **1**;^33,34,66−68^ therefore, the similarity in the CD spectra between compound **1** and its derivative thioethers in cyclohexane supports the hypothesis that they adopt a similar cross-β conformation stabilized by the same network of intramolecular hydrogen bonds in low-dielectric media.

To assess the low-dielectric conformations of the DLDD and DLDL stereochemical variants containing d-MeAla at position 4, CD spectra were obtained for the Library A and B representatives from each stereochemical group (**A1** and **B1** representing DLDD, and **A1–7L** and **B1–7L** representing DLDL). The CD spectra in cyclohexane of these four compounds were markedly different from those of decapeptide **1** and its thioether derivatives **LA02**, **LB07,** and **LC02** (Figure 5d,e), suggesting that this series adopts a low-dielectric conformation that, while capable of sequestering all four backbone NH groups from solvent, may be unique and somewhat distinct from the cross-β fold found in the parent scaffolds.

**Figure 6:**
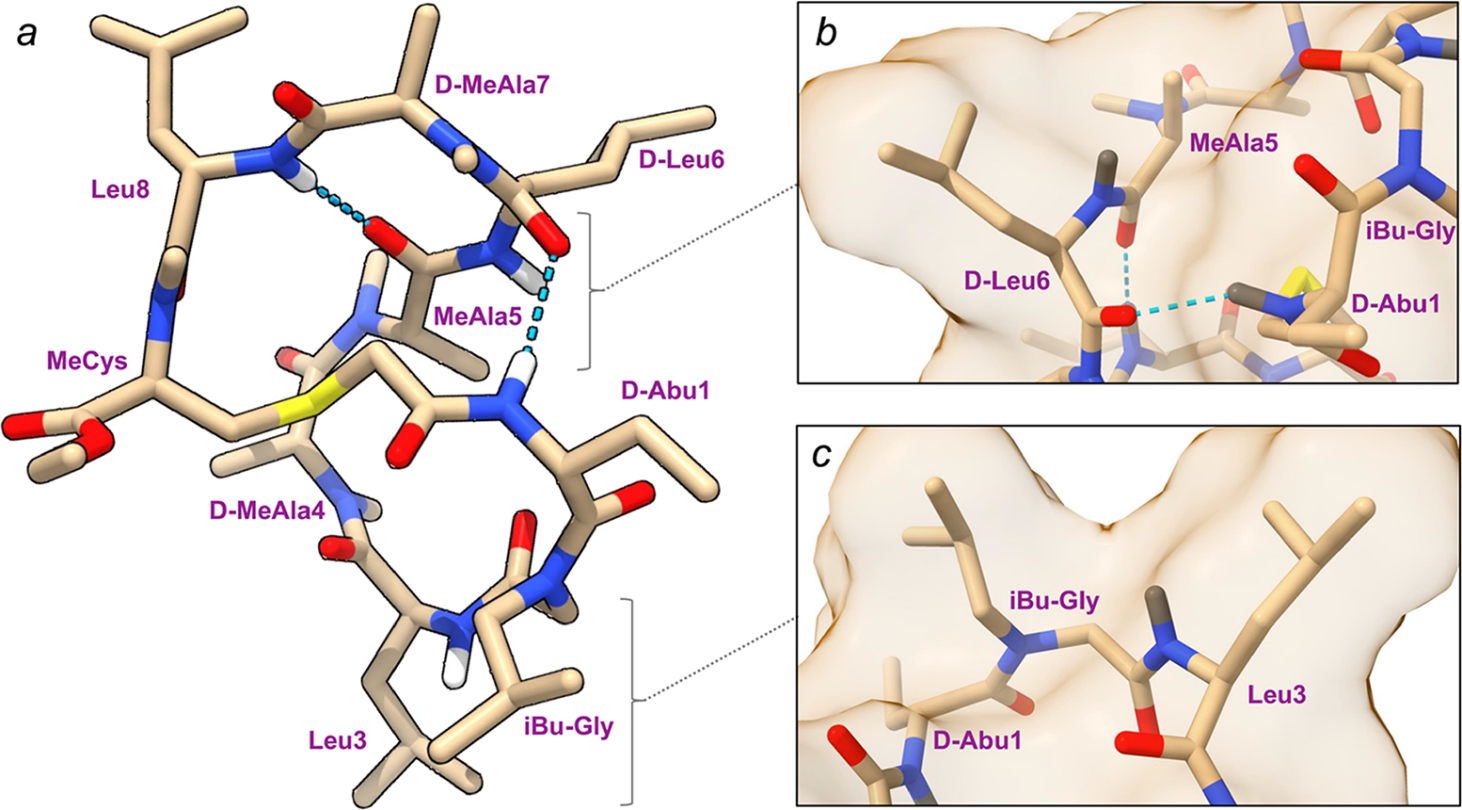
(a) NOE derived structure of **B1** in chloroform. Hydrogen bonds are shown in blue dashes. (b) Focused visualization of the hydrophobic surface area surrounding the NH amide moiety of d-Leu6. (c) Focused visual of the hydrophobic surface area surrounding the NH amide moiety of Leu3.

To gain further insight into the nature of this low-dielectric conformation, we investigated the solution NMR structure of **B1** in CDCl_3_. Key through-space interproton distances for **B1** were calculated by quantifying the cross-peak volumes identified in the ^1^H–^1^H EASY-ROESY spectrum (Table S3). These experimental interproton distances were then combined with NH-Hα *J*-coupling constants to provide distance and ϕ-torsional restraints respectively as input into CYANA structure calculations. The 20 lowest-energy conformers revealed a twisted saddle-shaped conformation containing two intramolecular hydrogen bonds (Figure S12). Both d-MeAla residues, positions 4 and 7, adopt the cis-amide configuration, one of which helps to template the hydrogen bond between Leu8 (donor) and MeAla5 (acceptor) (Figures 6a and S13). A second hydrogen bond between d-Abu1 (donor) and d-Leu6 (acceptor) serves to stitch the two lobes of the saddle together. The two remaining amide NH groups are sequestered from the solvent by the hydrophobic surface afforded by the neighboring aliphatic side chains (Figure 6b,c). This observation was confirmed via amide temperature coefficient NMR experiments in CDCl_3_ which yielded coefficient magnitudes <3 ppb/K for all amide NHs in **B1.**

In further support of the NMR-derived solution structure, we performed McMD simulations of **B1** in chloroform and cyclohexane solvent. Comparing the interproton distances of each conformation in both McMD trajectories with the NOE-derived interproton distances, an overall RMSD value for each conformer was determined. The lowest RMSD conformer in each ensemble demonstrates the same overall backbone fold as observed in the NMR structure (Figure S14). The 10 conformations of **B1** from the McMD ensemble in chloroform with the lowest RMSD to the calculated NOE distances from the EASY-ROESY spectrum in chloroform yield an average RMSD value of 1.16 Å, (Figure S15, Table S4), which decreases to 0.80 Å in the cyclohexane McMD ensemble (Figure S16, Table S5). Both ensembles contain the same two cis-amide bonds observed by NMR (Figure S13). Furthermore, these results support the observed hydrogen bonds in the NMR structure of **B1** and highlight the possibility of two additional intramolecular hydrogen bonds with minimal disturbance of the overall backbone shape. The d-Abu1 (NH)→ d-Leu6 (C=O) hydrogen bond is observed in 18% of the entire chloroform ensemble and 12% of the cyclohexane ensemble, yet the same two populations also exhibit two additional intramolecular hydrogen bonds: Leu3 (NH) → cyclothioacetyl (C=O) and d-Leu6 (NH) → d-Abu1 (C=O) (Figure S17). Surprisingly, Leu8 (NH) is more promiscuous in both the chloroform and cyclohexane McMD ensembles, participating either in a hydrogen bond with the carbonyl of either MeAla5 (as observed in the NMR structure) or d-Leu6. Given that the ROESY cross-peak volumes are determined by time-averaged interproton distances, the McMD trajectories provide a dynamic perspective on the solution behavior of **B1** that is complementary to the static ensembles derived from distance geometry calculations.

The saddle-shaped, low-dielectric conformation observed for **B1** is more spherical in shape than the elongated, rodlike conformations seen with other passively permeable MCPs such as Fouché scaffold and its derivatives, and cyclosporine A. In a recent report, molecular shape factors, or “*r*-values” from 0 (rod-like) to 1 (spherical) were determined for a series of cyclic peptides.^69^ The authors concluded that passive permeability in this size regime requires the ability to access rod-like conformations with *r*-values below ∼0.5, while more spherical conformations with *r*-values above ∼0.8, even ones that with extensive intramolecular hydrogen bonding, are likely to be impermeable. The more spherical low-dielectric conformation of **B1** has an *r*-value of 0.8, countering this hypothesis and suggesting that factors beyond the molecular shape alone may contribute to its passive permeability characteristics.

## Conclusions

Drawing inspiration from Fouché’s decapeptide **1** and Furukawa’s peptide/peptoid hybrids, all of which exhibit passive permeability and a proclivity for adopting a cross-β conformation in a low-dielectric solvent, we identified several passively permeable, thioether-cyclized MCP scaffolds, which led to the discovery of a novel permeable conformation. Incorporation of a thioether bond into the backbone preserved the core ring size of the parent MCPs, while modulation of scaffold lipophilicity aided in establishing the ideal lipophilic window for achieving permeability and aqueous solubility within this new scaffold space. Altering the stereochemistry of the parent scaffolds led to 96 additional compounds, whose diverse properties further underscored the interplay between lipophilicity, stereochemistry, and passive permeability. Circular dichroism experiments in the membrane-mimicking solvent cyclohexane verified that the cross-β conformation characteristic of the Fouché/Furukawa scaffolds was preserved in their thioether derivatives, while also revealing a different spectroscopic signature for a family of stereochemical variants containing d-MeAla in the turn at position 4. The NOE-derived solution-conformation in chloroform and McMD conformational ensembles indeed revealed a unique, saddle-shaped low-dielectric folded conformation of **B1** (Figure 6) in which all hydrogen bond donors (HBDs) are internally sequestered via either IMHB or neighboring steric occlusion.

Although the bulk of the 210 compounds analyzed in this work falls below the conventional threshold accepted as “permeable” for a lead compound in a drug campaign (*P*_app_ ∼ 1 × 10^–6^ cm/s), those that did display higher permeation rates, especially **A1** and **B1** (*P*_app_ ∼ 10 × 10^–6^ cm/s), are exceptionally permeable for a “beyond-Rule-of-5” (bRo5) macrocycle with four HBDs and MW ∼1000. For example, the potential efficacy of MCPs against hitherto intractable targets has been underscored by the cholesterol-lowering drug MK-0616, an oral MCP inhibitor of the interaction between PCSK9 and low-density lipoprotein.^70,71^ Due to its exquisite potency (*K*_i_ = 5 pM), MK-0616 has good clinical efficacy despite relatively poor oral bioavailability, which, because of the drug’s low membrane permeability, could only be achieved by using permeability enhancers.^72^

The highly potent and orally bioavailable LUNA18, an MCP derived from mRNA display that is in the same size range as the compounds described herein, is in clinical development against the challenging PPI between oncogenic KRAS and SOS. The optimization of the initial lead compound derived from an mRNA display library preserved the original basic scaffold while improving its membrane permeability 20-fold, from 0.02 × 10^–6^ cm/s to 0.4 × 10^–6^ cm/s in Caco-2 cells.^11^ This report highlights the untapped potential of the chemical space defined by MCPs in the 11-mer size range while also demonstrating that high potency and oral exposure can be optimized using traditional medicinal chemistry approaches even when starting with a scaffold whose permeability is quite low by traditional small molecule standards. Our results are also consistent with a recent report by Bhardwaj et al., who used computational approaches to design a variety of backbone-*N*-methylated MCPs with good permeabilities across a range of ring sizes (6–12 mers).^73^ Crystal structures of their most permeable scaffolds also showed diverse backbone geometries capable of forming complex IMHB networks. Given the enormous chemical space defined by MCPs in this size range when both backbone and side chain diversity are taken into account, there is little doubt that the potential for lead discovery against challenging intracellular targets is vast and remains largely unexplored. The relatively high permeabilities observed among the 96 backbone variants described in Figures 3 and 4 were not simply a consequence of the use of the Fouché peptide as the initial starting point, since a d-Pro to d-MeAla substitution led to a new series whose high permeability was achieved by accessing a novel, low-dielectric saddle-shaped conformation. Taken together, these results underscore the extent to which permeability can be achieved in diverse backbones, either by the preservation of a low-dielectric conformation or by accessing entirely new low-dielectric conformational states that facilitate the sequestration of polar backbone atoms in other ways. As high throughput drug discovery tools such as mRNA display continue to move toward larger, more complex scaffolds, both lead optimization and initial library design will benefit from continued efforts to illuminate the interplay between scaffold geometry and membrane permeability.

## Experimental Section

Detailed methods for synthesis, assays (permeability, solubility), analytical experiments (NMR, CD), and computational efforts (McMD, CYANA) are described thoroughly in the Supporting Information.

## Supporting Information Available

Detailed experimental procedures including supplemental figures and tables (PDF)
Complete permeability data and SMILES strings for all synthesized compounds (XLSX)
