# Supplementary material for: Membrane Permeability in a Large Macrocyclic Peptide Driven by a Saddle-Shaped Conformation
Source: J Am Chem Soc. 2024 Feb 8;146(7):4582–91. doi: 10.1021/jacs.3c10949 (PMC10885153; doi:10.1021/jacs.3c10949)
Supplement: Supplementary file 1 — ja3c10949_si_001.pdf [file ja3c10949_si_001.pdf]

Supporting Information

Membrane permeability in a large macrocyclic peptide driven by a saddle-shaped conformation

Authors and affiliations

Justin H. Faris[1,8], Emel Adaligil[2], Nataliya Popovych[3], Satoshi Ono[4], Mifune Takahashi[5], Huy Nguyen[6], Emile Plise[5], Jaru Taechalertpaisarn[1], Hsiau-Wei Lee[1], Michael F. T. Koehler[7], Christian N. Cunningham[2], and R. Scott Lokey[1]*

[1]Department of Chemistry and Biochemistry, University of California, Santa Cruz, California 95064, United States

[2]Department of Peptide Therapeutics, Genentech, South San Francisco, California 94080, United States

[3]Department of Early Discovery Biochemistry, Genentech, South San Francisco, California 94080, United States

[4]Innovative Research Division, Mitsubishi Tanabe Pharma Corporation, Kanagawa 227-0033, Japan

[5]Department of Drug Metabolism and Pharmacokinetics, Genentech, South San Francisco, California 94080, United States

[6]Department of Analytical Research, Genentech, South San Francisco, California 94080, United States

[7]Department of Medicinal Chemistry, Genentech, South San Francisco, California 94080, United States

[8]Department of Discovery Chemistry, Revolution Medicines, Inc., Redwood City, California 94063, United States (Present Address)

*Corresponding author: slokey@ucsc.edu

Table of Contents

Abbreviations......5
General Materials......5
Supplementary Tables & Figures......6
Table S1......6
Table S2......7
Table S3......8
Table S4......9
Table S5......10
Figure S1......11
Figure S2......12
Figure S3......13
Figure S4......14
Figure S5......15
Figure S6......15
Figure S7......16
Figure S8......17
Figure S9......17
Figure S10......18
Figure S11......18
Figure S12......19
Figure S13......19
Figure S14......20
Figure S15......21
Figure S16......22
Figure S17......23
Synthetic Procedures......24
Automated Synthesis......26
Manual synthesis......26
Synthesis of linear precursors......26
Peptoid addition......27
Cyclization......27
Purification......27
Fmoc-protection of deuterated AA's......27

N-methylation of AA's **28**

Analytical Procedures ***28***

NMR analysis **28**

UPLC-MS analysis **28**

PAMPA Mixture conditions 29
PAMPA Individual conditions 29
Purity Assessment conditions 29

PAMPA **30**

Primary protocol 30
Alternative PAMPA Assay 30

gMDCK Permeability Assay **31**

Kinetic Solubility PBS Protocol **31**

Circular Dichroism **32**

McMD protocol **32**

Individual compound validation of mixtures by MS ***33***

Liposcan **33**

Group A1 33
Group A2 33
Group B1 34
Group B2 34
Group C1 35
Group C2 35

Stereoscan **36**

Group ALL 36
Group ALD 36
Group ADL 37
Group ADD 37
Group A2L 38
Group A2D 38
Group BLL 39
Group BLD 39
Group BDL 40
Group BDD 40
Group B2L 41
Group B2D 41

Solution Structure Analysis of B1 .... ***42***

1D/2D NMR Spectra **42**

NMR Assignment of B1 .... **54**

Solution Structure Calculation using CYANA **55**

McMD data ***55***

Molecular Shape Analysis **55**

Principal Component Analysis **56**
Hydrogen-bond Plots **57**
Additional CD spectra ***59***
LCMS traces and NMR spectra of pure compounds ***60***
Decapeptide 1 .... **60**
Liposcan **61**
LA02 61
LA04 62
LA07 63
LA09 64
LB02 65
LB04 66
LB07 67
LB09 68
LC02 69
LC04 70
LC07 71
LC09 72
Stereoscan **73**
A1 73
A1-1L 74
A1-3D 76
A1-4Dpro 77
A1-6L 79
A1-7L 80
B1 82
B1-1L 83
B1-3D 85
B1-4Dpro 86
B1-6L 88
B1-7L 89
References ***91***

Abbreviations

DCM: Dichloromethane (CH_2Cl_2), DMF: N,N-dimethylformamide, EtOAc: ethyl acetate, HATU: 1-[Bis(dimethylamino)methylene]-1H-1,2,3-triazolo[4,5-b]pyridinium 3-oxide hexafluorophosphate, DIPEA: N,N-diisopropylethylamine, HOAt: 1-Hydroxy-7-azabenzotriazole, COMU: (1-Cyano-2-ethoxy-2-oxoethylideneaminooxy)dimethylamino-morpholinocarbenium hexafluorophosphate, PyBOP: benzotriazol-1-yloxytripyrrolidinophosphonium hexafluorophosphate, DIC: N,N-diisopropylcarbodiimide, DBU: 1,8-diazabicyclo[5.4.0]undec-7-ene, TFA: trifluoroacetic acid, Trt: trityl, Fmoc: 9-fluorenylmethyloxycarbonyl, ACN: acetonitrile, HPLC-MS: high-performance liquid chromatography – mass spectrometry, PAMPA: parallel artificial membrane permeability assay, DMSO: dimethyl sulfoxide, UPLC-MS: ultra-performance liquid chromatography – mass spectrometry, PBS: phosphate buffer solution (pH 7.4), SPE: solid-phase extraction, NMR: nuclear magnetic resonance, HFIP: 1,1,1,3,3,3-hexafluoroisopropanol, mRNA: messenger ribonucleic acid, McMD: multicanonical molecular dynamics, PPI: protein-protein interaction, Ro5: Rule of 5, bRo5: beyond Rule of 5, DNA: deoxyribonucleic acid, tRNA: transfer ribonucleic acid, MW: molecular weight, SPPS: solid-phase peptide synthesis, AlogP: calculated octanol/water partition coefficient, MDCK: Madin-Darby canine kidney cells

General Materials

Most solvents were purchased from Fischer Scientific (HPLC grade; except ethyl acetate, 1,4-dioxane, and DMF which were purchased at ACS grade). Ethanol (200 proof) was purchased from Gold Shield. Cyclohexane (HPLC grade) was purchased from Sigma-Aldrich. Dry solvents were obtained by soaking overnight over 4 Å molecular sieves (Fisher Scientific). UPLC solvents, including formic acid, were purchased from Fisher Scientific (Optima grade). Most Fmoc amino acids were purchased from combi-blocks except Fmoc-Leu (L from Novabiochem, D from Oakwood) and Fmoc-L-Pro (Sigma-Aldrich). Deuterated amino acids were purchased from Cambridge Isotope Laboratories. HATU and COMU were purchased from combi-blocks. HOAt was purchased from aapptec. DIC, Fmoc-OSu, triethylsilane, isobutylamine, TFA, and HFIP were all purchased from Oakwood. DIPEA and bromoacetic acid were purchased from Thermo Scientific. Piperidine was purchased from Spectrum. Ethylamine was purchased as a 10% solution in THF from TCI. Propylamine was purchased from Acros. (+/-) camphorsulfonic acid, paraformaldehyde, n-dodecane, and lecithin (90%, soybean) were purchased from Alfa-Aesar. 10x PBS (pH 7.4) was purchased from Fisher Scientific and diluted for use. 37% HCl, and sodium bicarbonate were purchased from Fisher Scientific. Deuterated chloroform was purchased in ampoules from Thermo Scientific. Magnesium sulfate was purchased from Sigma-Aldrich. Preloaded H-Leu-2-CTC-PS resin was purchased from Rapp Polymere (H0370753316) and received at a loading value of 0.65 mmol/g. Argon gas was sourced from ProSpec (Praxair).

Supplementary Tables & Figures

Table S1

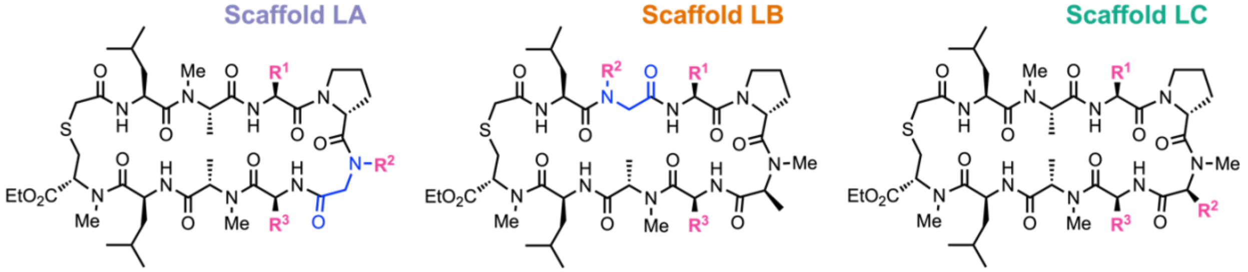

Table S1: Resynthesized Thioether Liposcan Compounds

Cpd.	R1	R2	R3	AlogP	MW	AQ solubility (μM)	PAMPA P_{app} ($x10^{-6}$ cm/s) (mixture)	PAMPA P_{app} ($x10^{-6}$ cm/s) (individual)	PAMPA P_{app} ($x10^{-6}$ cm/s) (individual)*	MDCK P_{app} ($x10^{-6}$ cm/s) (A to B)
LA02	Leu	Et	Abu	1.44	980	200	4.9 ± 0.2	4.1 ± 0.4	11.7 ± 2.7	4.5
LA04	Leu	Pr	Abu	1.97	994	200	4.0 ± 0.1	3.9 ± 0.2	5.2 ± 1.0	4.0
LA07	Nva	Pr	Leu	2.42	1008	122	3.0 ± 0.1	1.3 ± 0.4	4.8 ± 0.8	8.4
LA09	Nva	Et	Cha	2.90	1034	10	0.1 ± 0.0	0.2 ± 0.1	2.1 ± 0.2	5.1
LB02	Leu	Et	Abu	1.44	980	126	1.0 ± 0.2	1.1 ± 0.2	7.5 ± 0.3	1.8
LB04	Leu	Pr	Abu	1.97	994	105	1.6 ± 0.3	1.9 ± 0.3	--	2.6
LB07	Nva	Pr	Leu	2.42	1008	132	3.1 ± 0.4	2.2 ± 0.8	--	3.2
LB09	Nva	Et	Cha	2.90	1034	19	1.1 ± 0.6	1.4 ± 0.4	4.4 ± 0.8	4.4
LC02	Leu	Et	Abu	2.10	994	200	5.0 ± 0.1	2.6 ± 0.2	11.8 ± 4.4	7.4
LC04	Leu	Pr	Abu	2.56	1008	183	3.5 ± 0.0	3.3 ± 0.2	8.4 ± 2.1	6.5
LC07	Nva	Pr	Leu	3.01	1022	109	2.1 ± 0.2	1.1 ± 0.4	4.5 ± 0.8	3.9
LC09	Nva	Et	Cha	3.55	1048	10	0.3 ± 0.1	0.3 ± 0.1	1.4 ± 0.3	1.8

*Alternative protocol used. See Supporting Information for details.

Table S2

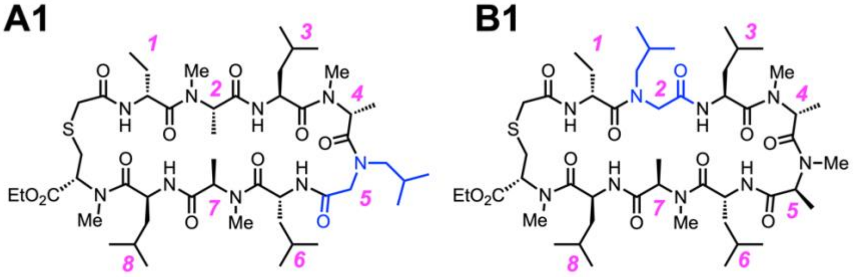

Table S2: Resynthesized Thioether Stereoscan Compounds

Cpd.	Stereochemistry at position 1	3	6	7	Identity at position 4	2	5	AlogP	MW	PAMPA P_{app} ($x10^{-6}$ cm/s) (mixture)	PAMPA P_{app} ($x10^{-6}$ cm/s) (individual)	MDCK P_{app} ($x10^{-6}$ cm/s) (A to B)
**A1**	D	L	D	D	D-MeAla	L-MeAla	N-iBuGly	2.17	995	7.9 ± 0.5	10.0 ± 0.5	9.02
**A1-1L**	L	L	D	D	D-MeAla	L-MeAla	N-iBuGly	2.17	995	1.1 ± 0.0	1.2 ± 0.1	0.93
**A1-3D**	D	D	D	D	D-AlaMe	L-MeAla	N-iBuGly	2.17	995	1.5 ± 0.1	2.8 ± 0.1	1.25
**A1-4dP**	D	L	D	D	D-Pro	L-MeAla	N-iBuGly	2.28	1007	0.1 ± 0.0	0.4 ± 0.1	0.02
**A1-6L**	D	L	L	D	D-MeAla	L-MeAla	N-iBuGly	2.17	995	0.7 ± 0.0	1.4 ± 0.1	0.86
**A1-7L**	D	L	D	L	D-MeAla	L-MeAla	N-iBuGly	2.17	995	6.8 ± 0.2	10.4 ± 0.5	6.41
**B1**	D	L	D	D	D-MeAla	N-iBuGly	L-MeAla	2.17	995	6.1 ± 0.2*	10.1 ± 2.1	1.27
**B1-1L**	L	L	D	D	D-MeAla	N-iBuGly	L-MeAla	2.17	995	0.6 ± 0.0*	0.5 ± 0.0	0.03
**B1-3D**	D	D	D	D	D-AlaMe	N-iBuGly	L-MeAla	2.17	1007	2.5 ± 0.7*	1.5 ± 0.1	0.48
**B1-4dP**	D	L	D	D	D-Pro	N-iBuGly	L-MeAla	2.28	995	0.1 ± 0.0*	0.2 ± 0.0	0.03
**B1-6L**	D	L	L	D	D-MeAla	N-iBuGly	L-MeAla	2.17	995	0.5 ± 0.1*	0.7 ± 0.0	0.39
**B1-7L**	D	L	D	L	D-MeAla	N-iBuGly	L-MeAla	2.17	995	5.2 ± 0.2*	5.7 ± 0.1	1.21

*B1 compounds in mixture anlaysis contain N-PrGly peptoid instead of N-iBuGly.

Table S3

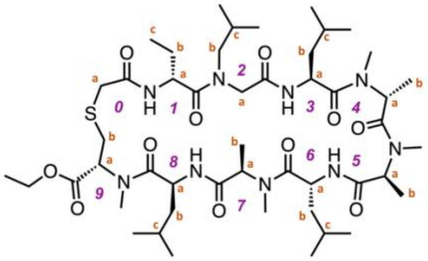

Table S3: Experimental NOE distances of **B1** calculated from the EASY-ROESY spectrum in chloroform.

No.	Correlation (A-B)	δ^1Ha	δ^1Hb	r_{ab} (Å)	upper limit (Å)	lower limit (Å)
1	1NH-6NH	8.54	7.34	3.25	3.60	2.60
2	1NH-6Hb1	8.54	1.74	3.15	4.50	3.50
3	1NH-0Ha1	8.54	3.79	3.43	5.50	3.80
4	1NH-0Ha2	8.54	2.82	2.33	3.00	2.00
5	1NH-1Hb2	8.54	1.07	3.01	3.50	2.50
6	1NH-1Hb1	8.54	1.98	2.59	3.00	2.00
7	8NH-6Ha	7.63	5.10	3.18	4.50	3.50
8	8NH-8Hb2	7.63	1.41	2.60	3.50	2.50
9	8NH-7Ha	7.63	5.28	2.22	2.50	1.80
10	6NH-6Hb1	7.34	1.74	2.76	4.00	3.00
11	6NH-6Hb2	7.34	1.66	2.72	4.00	3.00
12	6NH-5Hb	7.34	1.34	2.28	2.50	1.80
13	6NH-5Ha	7.34	4.63	2.95	3.50	2.50
14	3NH-0Ha1	6.88	3.79	4.17	7.00	4.60
15	3NH-2Hc	6.88	1.92	4.20	6.00	4.60
16	3NH-2Ha1	6.88	3.42	3.58	4.00	3.00
17	3NH-3Hb1	6.88	1.48	3.22	4.00	3.00
18	3NH-3Hb2	6.88	1.58	2.75	3.00	2.00
19	3NH-2Hb1	6.88	4.18	3.06	3.50	2.50
20	3NH-3Ha	6.88	5.03	2.96	3.30	2.30
21*	3NH-2Hd	6.88	1.04	3.69	7.00	4.10
22	3NH-2Hb2	6.88	2.86	2.93	3.00	2.00
23	9Ha-4Hb	5.87	1.02	3.13	4.00	3.00
24	9Ha-4Ha	5.87	6.03	2.70	4.00	3.00
25	3Ha-5Hb	5.03	1.34	3.16	5.00	3.50
26	4Ha-0Ha1	6.03	3.79	3.22	3.50	2.50
27	4Ha-5Ha	6.03	4.63	2.07	2.50	1.80
28	9Ha-0Ha1	5.87	3.79	2.28	2.50	1.80
29	7Ha-6Ha	5.28	5.1	1.86	2.50	1.80
30	6Ha-7Hb	5.10	1.39	3.26	5.00	3.60
31	1Ha-1Hc	4.59	1.07	2.77	3.50	2.50
32	1Ha-2Hb1	4.59	4.18	2.16	3.00	2.00
33	2Ha1-2Hc	3.42	1.92	2.67	4.50	3.00
34	9Hb1-0Ha1	3.60	3.79	3.57	4.50	3.50
35	9Hb2-0Ha1	2.46	3.79	3.56	6.00	3.90
36	4Ha-2Hb2	6.03	2.86	3.00	3.50	2.50
37	8Ha-7Nme	4.89	2.79	5.00	6.00	4.00
38	5Ha-2Hb2	4.63	2.86	4.75	5.50	4.00
39	5Ha-0HA1	4.63	3.79	5.00	6.00	4.00
Ref.	1Hb1-Hb2	1.98	1.72	1.78		

*Left out of McMD RMSD calculations due to conformational flexibilty of the sidechain

Table S4

Table S4: The 10 conformations of **B1** from the McMD ensemble in chloroform with the lowest RMSD to the calculated NOE distances from the EASY-ROESY spectrum in chloroform.*

NOE No.	Interproton distances from McMD ensemble in chloroform by conformer number										NOE r_{ab} (Å)
	**953**	**540**	**539**	**160**	**886**	**812**	**888**	**885**	**381**	**221**	
**1**	4.17	3.29	2.72	3.23	3.19	2.92	3.71	3.21	4.02	3.60	*3.25*
**2**	4.29	4.34	2.55	3.79	4.33	4.21	4.89	4.63	4.86	4.28	*3.15*
**3,4**	2.90	3.01	2.78	2.74	2.90	2.82	3.16	3.15	2.80	3.05	*2.88*
**5,6**	3.22	2.79	3.04	3.12	3.16	3.42	3.10	3.08	2.53	2.94	*2.80*
**7**	4.59	3.77	3.69	3.39	4.28	3.59	4.26	4.01	4.06	4.40	*3.18*
**8**	2.39	3.08	2.74	2.79	3.42	3.05	2.94	2.99	3.28	3.18	*2.60*
**9**	2.63	3.68	3.08	3.54	3.57	3.25	3.65	3.46	3.65	3.50	*2.22*
**10,11**	2.51	3.24	2.36	2.68	2.96	2.97	3.17	3.28	3.22	3.00	*2.74*
**12**	2.78	3.01	3.30	2.82	2.99	3.37	3.69	3.71	3.25	3.08	*2.28*
**13**	2.94	2.53	2.27	2.79	2.57	2.50	2.19	2.33	2.46	2.59	*2.95*
**14**	4.32	4.30	4.66	4.25	4.93	5.76	3.75	4.28	3.85	3.86	*4.17*
**15**	4.88	4.75	2.80	3.50	4.75	3.03	4.59	4.31	4.59	4.57	*4.20*
**16**	3.35	3.39	3.39	3.41	3.28	3.16	3.33	3.18	3.39	3.29	*3.58*
**17,18**	3.10	2.95	3.28	3.12	3.44	3.24	2.90	2.81	3.30	3.06	*2.99*
**19,22**	2.86	2.71	4.03	4.31	2.62	3.87	2.69	2.58	2.98	2.64	*3.00*
**20**	3.00	2.95	2.96	2.96	2.97	2.93	2.94	2.72	3.00	2.99	*2.96*
**23**	3.39	4.30	3.90	3.23	5.35	3.78	5.27	5.64	5.99	5.99	*3.13*
**24**	2.81	3.88	2.98	2.84	4.84	3.59	5.69	5.84	5.80	5.87	*2.70*
**25**	3.72	4.58	3.81	4.07	3.68	4.27	5.18	5.67	4.91	5.35	*3.16*
**26**	2.90	3.65	2.79	3.08	3.75	4.40	4.83	4.74	4.47	4.85	*3.22*
**27**	2.01	2.24	1.98	1.92	2.12	2.22	1.95	2.24	2.50	2.18	*2.07*
**28**	2.60	2.74	2.26	2.46	3.09	3.79	2.52	2.48	2.80	2.57	*2.28*
**29**	4.36	4.52	4.69	4.75	4.81	4.54	4.79	4.58	4.52	4.57	*1.86*
**30**	5.65	5.44	5.35	5.29	5.48	5.31	5.37	5.44	5.54	5.42	*3.26*
**31**	2.92	2.94	3.00	3.18	3.18	3.21	3.01	2.98	2.93	2.79	*2.77*
**32**	2.71	2.86	2.66	2.91	2.86	2.73	2.77	2.68	2.68	2.71	*2.16*
**33**	3.98	4.36	3.29	2.96	4.05	3.15	2.86	2.68	2.76	2.98	*2.67*
**34,35**	3.53	3.43	3.44	3.51	3.56	3.48	3.59	3.40	3.72	3.52	*3.57*
**36**	6.85	7.44	7.75	8.11	6.87	8.00	7.17	7.17	7.52	7.42	*5.00*
**37**	5.01	5.89	6.17	6.05	5.88	6.24	5.68	5.67	5.95	6.09	*5.00*
**38**	6.59	6.44	6.98	7.02	6.13	7.09	6.22	6.10	6.10	6.30	*4.75*
**39**	3.66	4.41	3.36	3.38	4.55	5.26	5.47	5.59	5.15	5.54	*5.00*
**RMSD**	**0.95**	**1.06**	**1.09**	**1.09**	**1.13**	**1.17**	**1.26**	**1.28**	**1.29**	**1.30**	

*Interproton distances for each NOE are shown (Å) as well as overall RMSD values ($Å^2$, bottom row).

Table S5

Table S5: The 10 conformations of **B1** from the McMD ensemble in cyclohexane with the lowest RMSD to the calculated NOE distances from the EASY-ROESY spectrum in chloroform.*

NOE No.	Interproton distances from McMD ensemble in cyclohexane by conformer number										NOE r_{ab} (Å)
	286	285	630	274	927	631	928	162	275	629	
**1**	3.90	3.45	3.12	3.39	3.24	3.21	3.46	3.35	3.81	2.72	*3.25*
**2**	4.79	3.88	4.18	4.45	4.75	4.33	4.28	4.58	5.08	3.65	*3.15*
**3,4**	3.20	3.04	3.01	3.19	3.29	2.97	3.24	2.78	3.31	2.97	*2.88*
**5,6**	3.31	3.17	2.94	2.86	3.08	3.14	3.01	3.35	3.15	2.95	*2.80*
**7**	3.45	3.38	3.42	3.24	2.52	2.87	3.51	3.49	3.09	3.13	*3.18*
**8**	2.65	2.90	2.67	2.67	2.57	2.43	2.71	2.63	2.60	2.66	*2.60*
**9**	2.04	2.20	1.98	2.31	2.46	2.30	2.23	1.98	2.35	2.02	*2.22*
**10,11**	2.68	3.07	3.01	2.50	2.81	2.61	2.52	2.77	2.61	2.83	*2.74*
**12**	2.99	3.06	3.05	2.96	3.12	2.92	2.72	3.08	3.12	2.97	*2.28*
**13**	2.47	2.47	2.45	2.69	2.17	2.64	2.76	2.26	2.63	2.58	*2.95*
**14**	4.18	4.06	3.98	4.04	4.39	4.16	3.95	3.93	4.38	3.89	*4.17*
**15**	4.21	5.09	5.20	5.17	4.77	4.78	4.84	4.88	4.96	4.64	*4.20*
**16**	3.32	3.51	3.42	3.48	3.36	3.29	3.51	3.34	3.43	3.20	*3.58*
**17,18**	3.00	3.05	3.53	3.08	3.01	3.21	3.10	3.18	3.05	3.25	*2.99*
**19,22**	2.52	3.06	3.40	3.14	2.91	3.10	2.95	3.60	3.09	2.98	*3.00*
**20**	2.98	2.94	2.89	2.95	3.00	3.05	3.05	3.00	3.00	3.01	*2.96*
**23**	4.03	3.63	4.29	3.65	3.94	4.50	4.16	4.12	4.09	5.49	*3.13*
**24**	3.11	2.74	2.80	2.75	3.46	3.39	2.98	3.70	2.60	3.94	*2.70*
**25**	4.10	3.52	3.44	3.74	4.08	3.95	4.27	4.33	3.79	4.40	*3.16*
**26**	3.16	3.36	3.63	3.19	4.02	3.66	3.06	3.57	4.70	4.04	*3.22*
**27**	2.06	1.94	2.47	1.94	1.92	1.90	2.11	1.94	2.04	2.10	*2.07*
**28**	2.49	2.77	2.66	2.29	2.75	2.31	2.40	2.60	3.43	2.35	*2.28*
**29**	2.02	1.98	1.99	1.95	1.79	1.93	1.86	1.95	1.98	1.93	*1.86*
**30**	3.81	4.05	3.73	4.02	4.16	4.19	3.56	3.52	4.19	3.96	*3.26*
**31**	3.18	3.22	3.14	3.20	3.21	3.13	2.93	3.30	3.43	3.04	*2.77*
**32**	2.81	2.80	2.77	2.78	2.70	2.72	2.74	2.65	2.87	2.87	*2.16*
**33**	2.79	3.91	3.00	4.08	4.05	2.82	4.41	2.65	4.09	2.76	*2.67*
**34,35**	3.36	3.67	3.57	3.58	3.21	3.49	3.50	3.32	3.10	3.57	*3.57*
**36**	6.77	7.01	7.52	7.36	7.14	7.48	7.49	7.98	7.49	7.79	*5.00*
**37**	4.70	5.36	5.34	4.90	5.28	5.54	5.37	5.38	5.35	5.54	*5.00*
**38**	6.14	6.54	6.73	6.75	6.59	6.72	6.45	6.89	6.66	6.80	*4.75*
**39**	3.75	3.30	3.98	3.42	4.49	3.34	3.02	3.85	5.04	3.43	*5.00*
**RMSD**	**0.66**	**0.71**	**0.75**	**0.77**	**0.78**	**0.80**	**0.81**	**0.87**	**0.88**	**0.92**	

*Interproton distances for each NOE are shown (Å) as well as overall RMSD values (Å^2, bottom row).

Figure S1

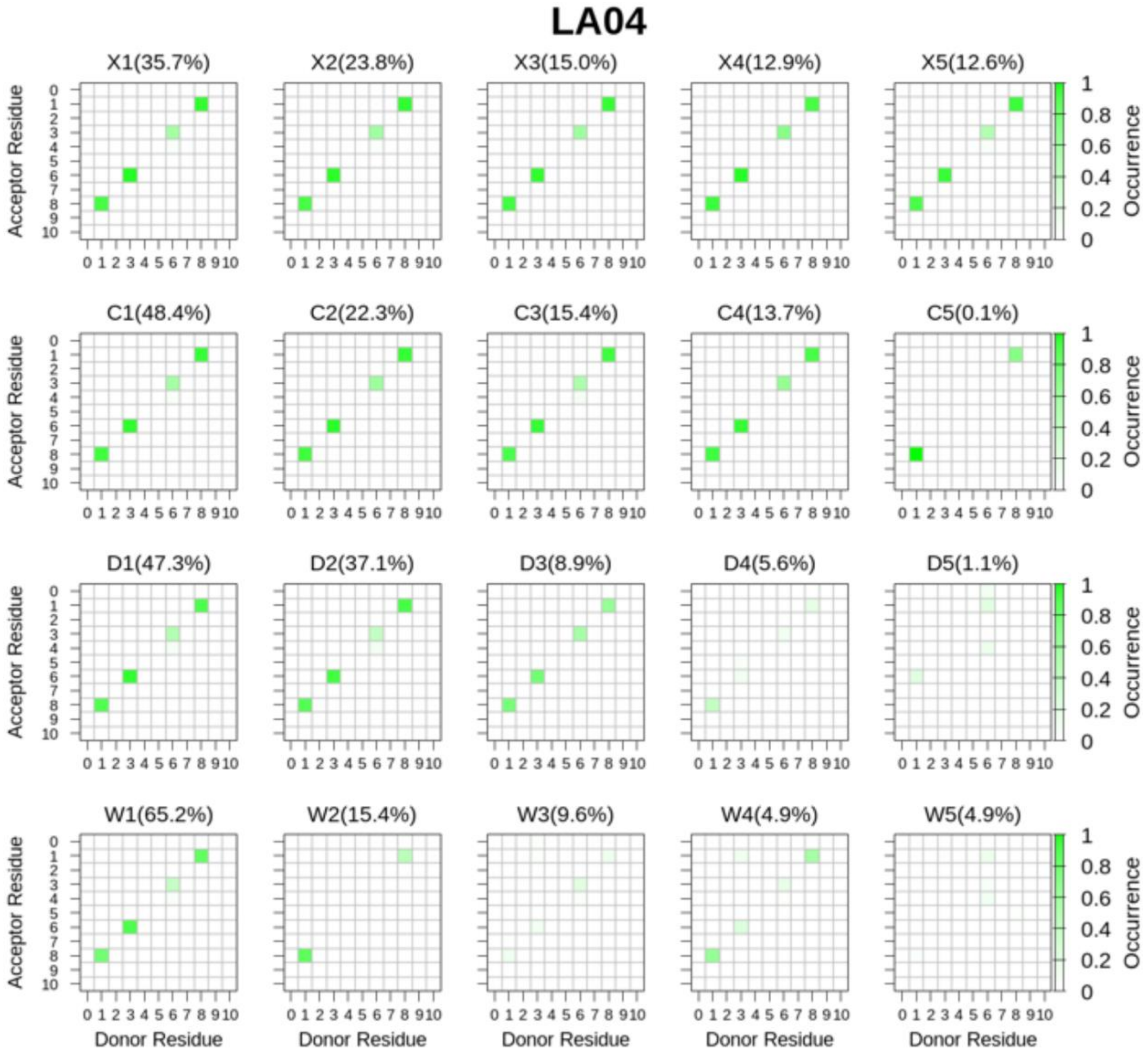

Figure S1: McMD hydrogen-bond plots for **LA04** (X = cyclohexane, C = chloroform, D = DMSO, W = water). The percentages reflect the portion of the 1000-member conformational ensemble that each plot represents.

Figure S2

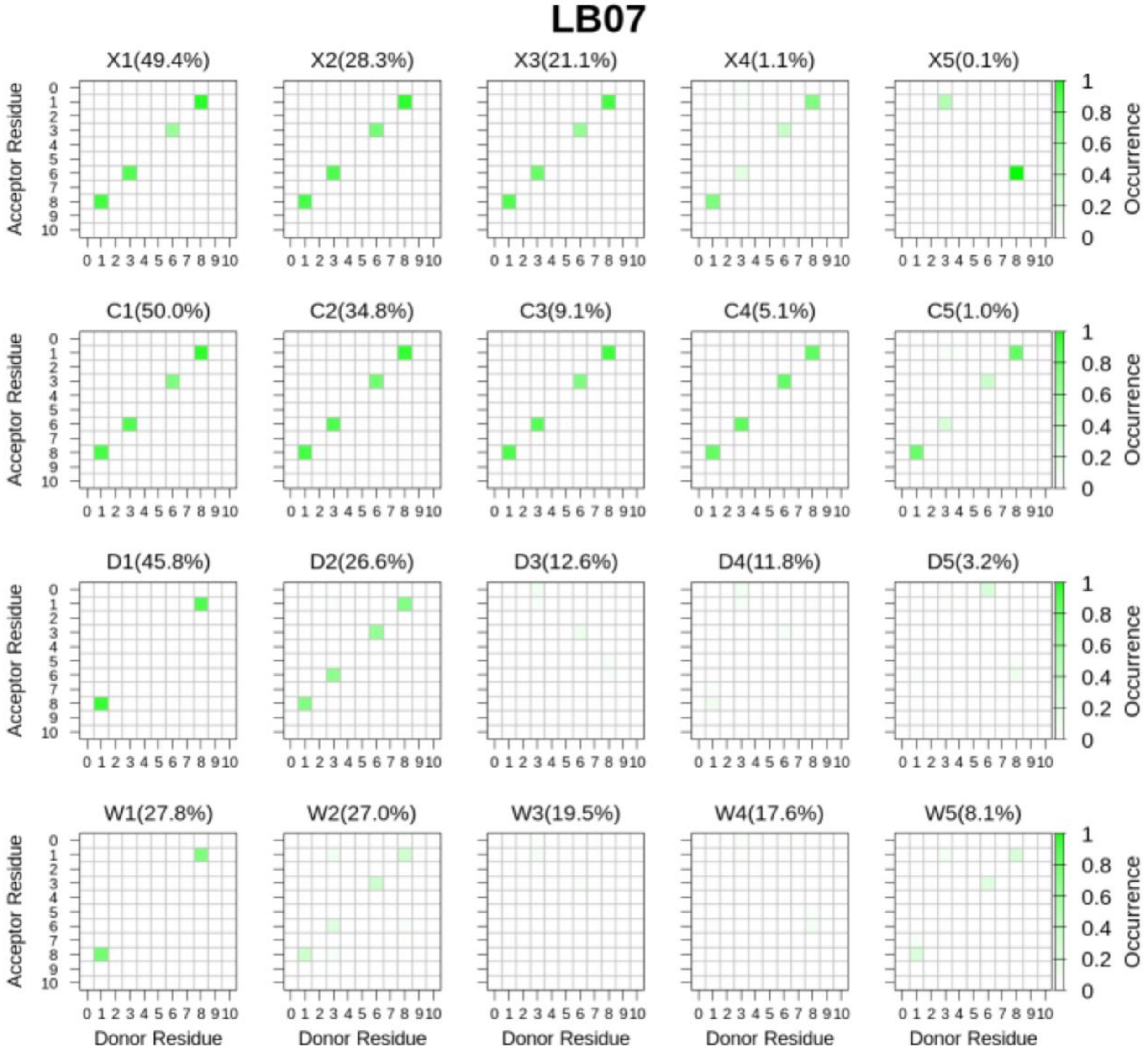

Figure S2: McMD hydrogen-bond plots for **LB07** (X = cyclohexane, C = chloroform, D = DMSO, W = water). The percentages reflect the portion of the 1000-member conformational ensemble that each plot represents.

Figure S3

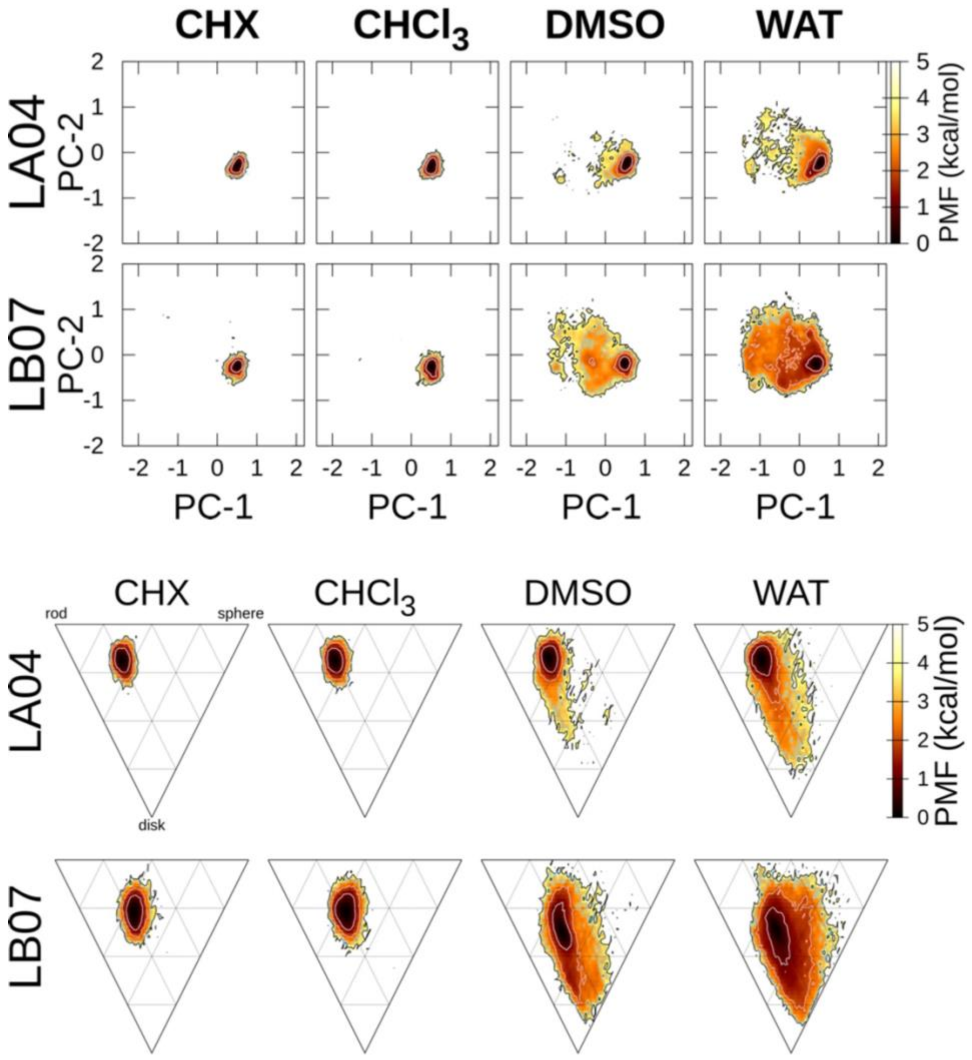

Figure S3: Free-energy landscapes of **LA04** and **LB07** (top). Molecular shape analysis of **LA04** and **LB07** (bottom). (CHX = cyclohexane, $CHCl_3$ = chloroform, WAT = water)

Figure S4

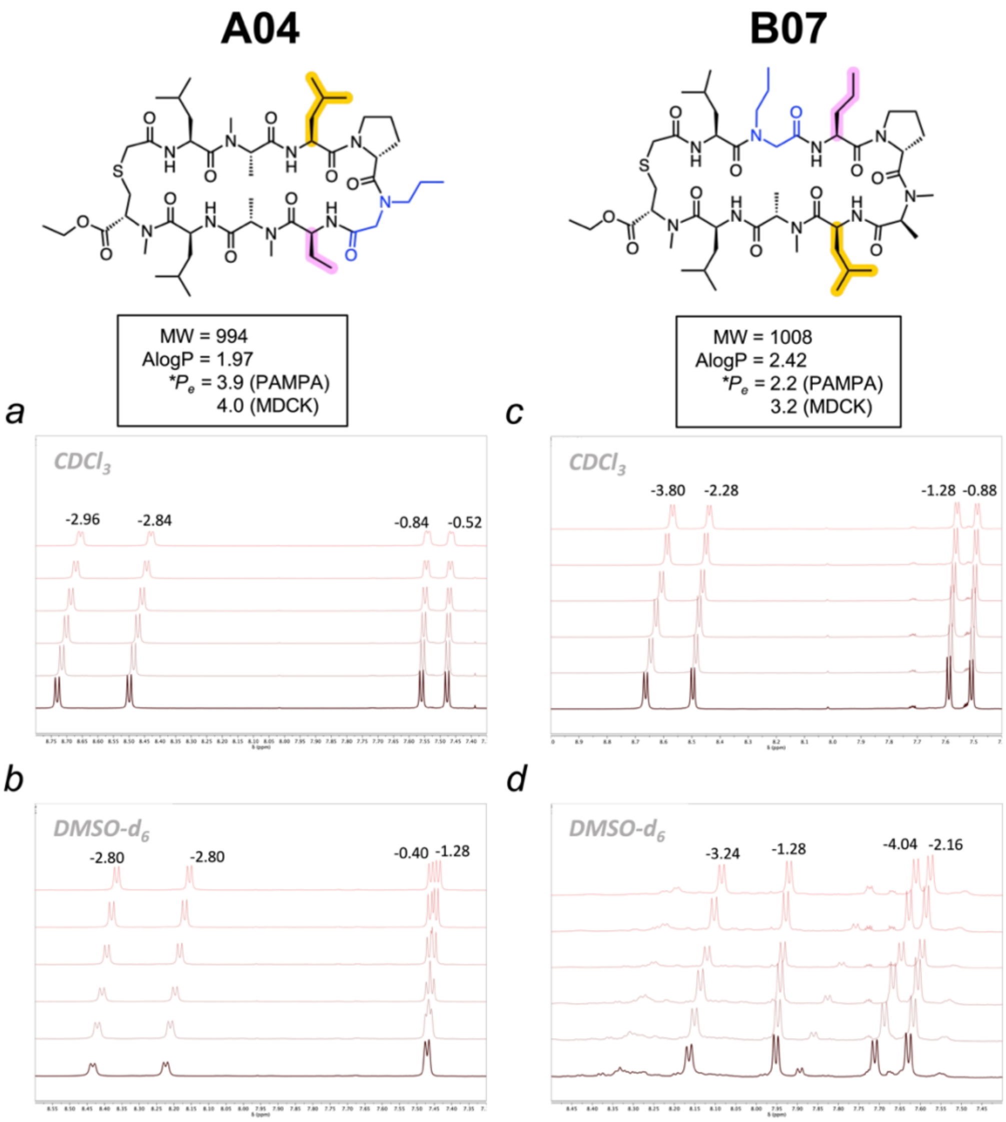

Figure S4: Amide temperature coefficient NMR analysis of **LA04** and **LB07** in $CDCl_3$ (a, c) and DMSO-d_6 (b, d). Numbers shown above each peak are the temperature coefficient values (ppb/K) of the amide hydrogens. *Permeability values (PAMPA and MDCK) are expressed as (value) x 10^{-6} cm/s. Temperatures raise from the bottom up in 5 degree increments respectively from 298K – 323 K

Figure S5

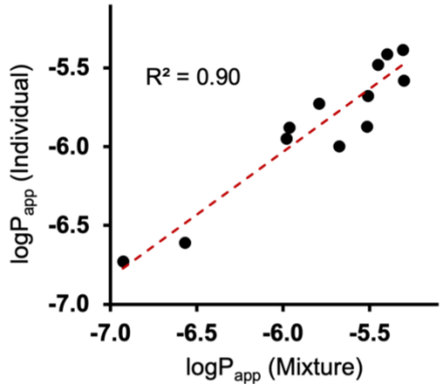

Figure S5: Scatter plot relating the liposcan permeability values of the mixture measurement to the individual measurement.

Figure S6

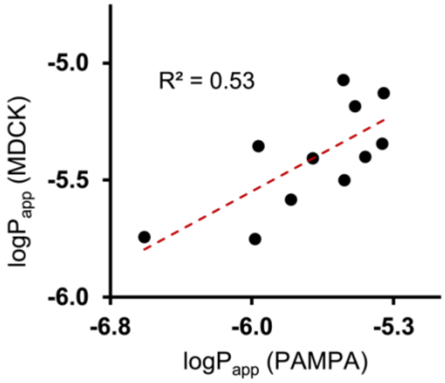

Figure S6: Scatter plot showing the correlation between PAMPA and MDCK permeability measurements of the resynthesized liposcan compounds.

Figure S7

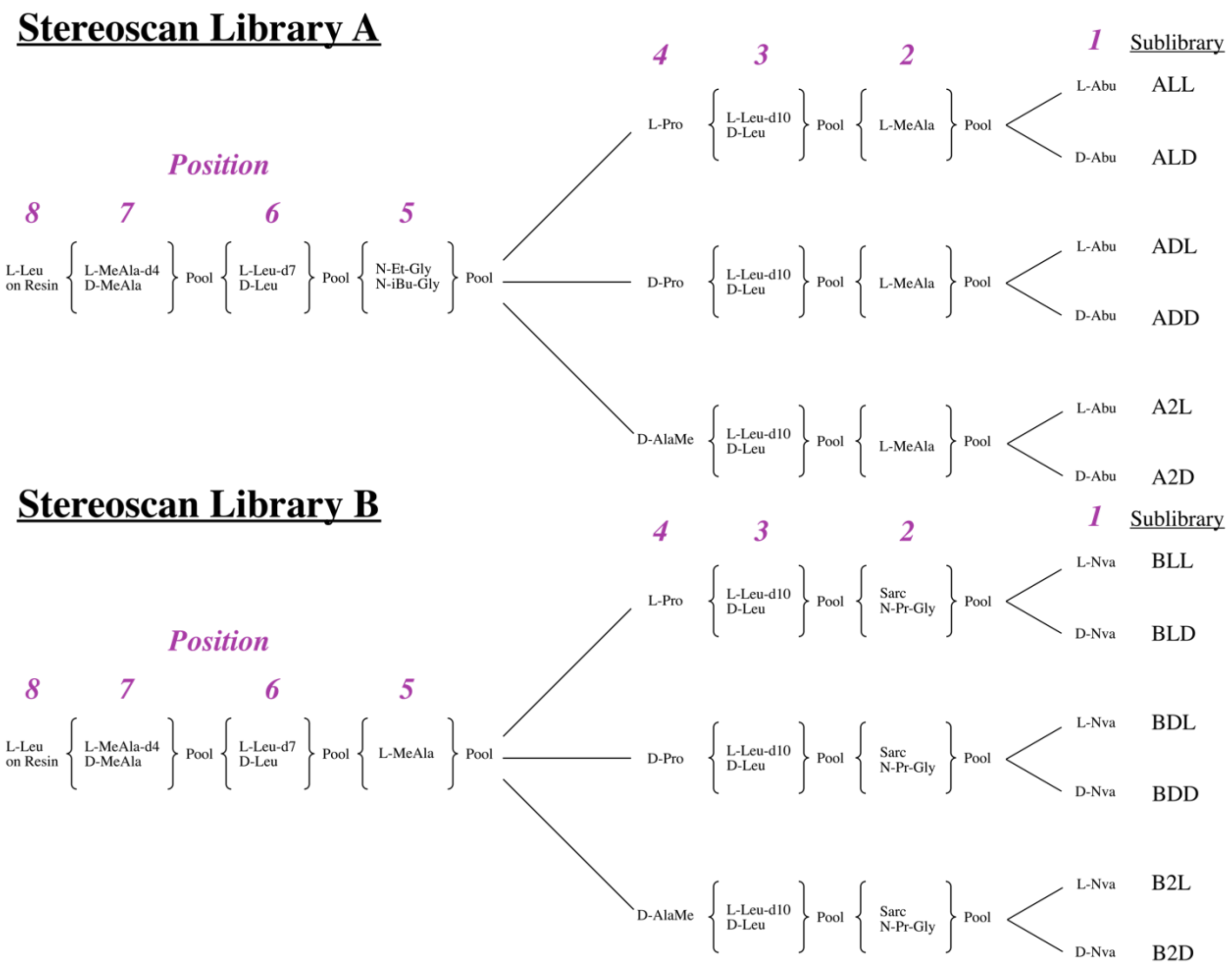

Figure S7: Diagram of the synthetic splitting scheme for stereoscan libraries A (top) and B (bottom). Experimental sub-library groups are denoted to the right. Strategic splitting at position 1 and 4 along with the utilization of deuterated L-amino acids were utilized at position 3, 6, and 7 to yield a unique mass for each compound when pooled together in each sub-library.

Figure S8

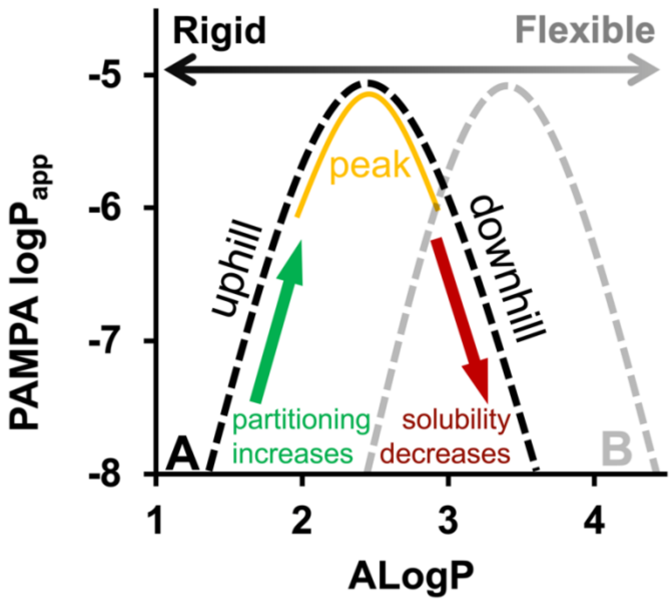

Figure S8: Diagram explaining the relationship between lipophilicity (ALogP), PAMPA permeability, and scaffold character (flexible vs. rigid). Data set A and B represent two separate but similar scaffolds.

Figure S9

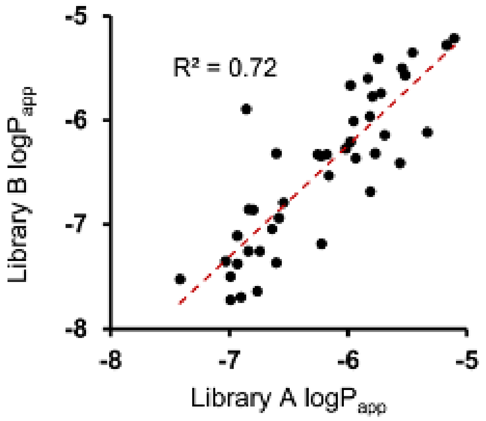

Figure S9: Scatter plot showing the relationship between matched pairs of compounds between library A and library B of the stereo-scan. In this plot only the compounds in the "high" AlogP class were shown. Compounds below the limit of detection were omitted.

Figure S10

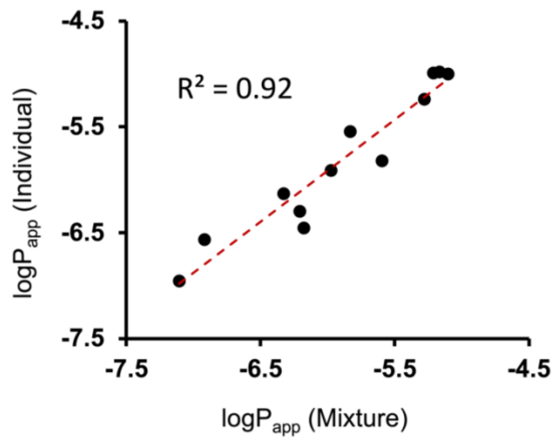

Figure S10: Scatter plot relating the PAMPA permeability values of bulk measurement to individual measurement of resynthesized stereoscan compounds.

Figure S11

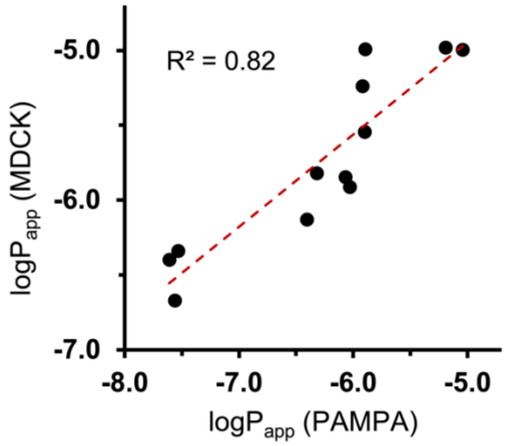

Figure S11: Scatter plot showing the correlation between PAMPA and MDCK permeability measurements of the resynthesized stereoscan compounds.

Figure S12

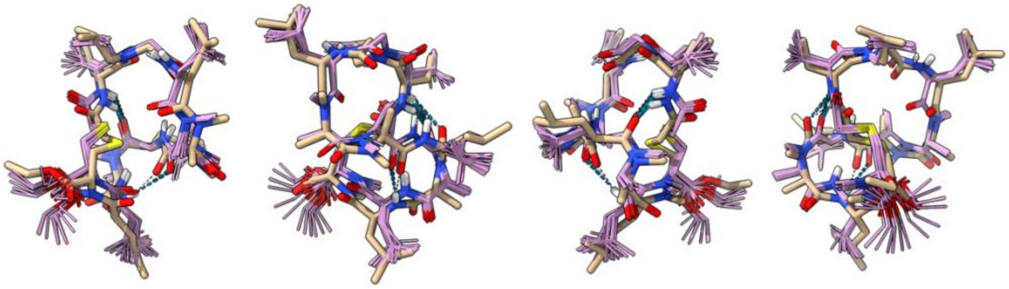

Figure S12: Ensemble of the 20 lowest energy structures (purple) calculated by CYANA of **B1** overlaid with the energy minimized structure in chloroform. The four pictures are the same 21 overlaid structures shown from different angles.

Figure S13

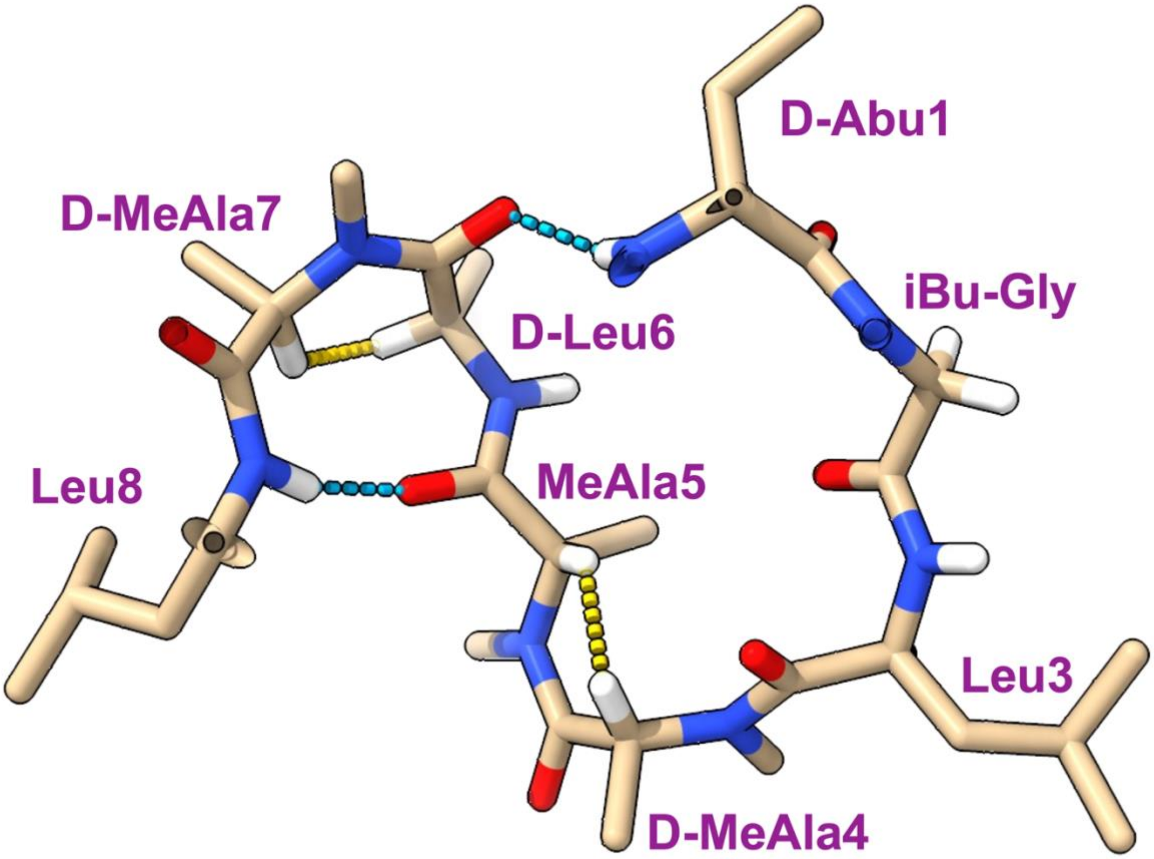

Figure S13: Highlight of the cis-amide bonds in the chloroform structure of **B1** and the NOE interactions (yellow dashes) which helped identify them. Hydrogen-bonds are in blue dashes.

Figure S14

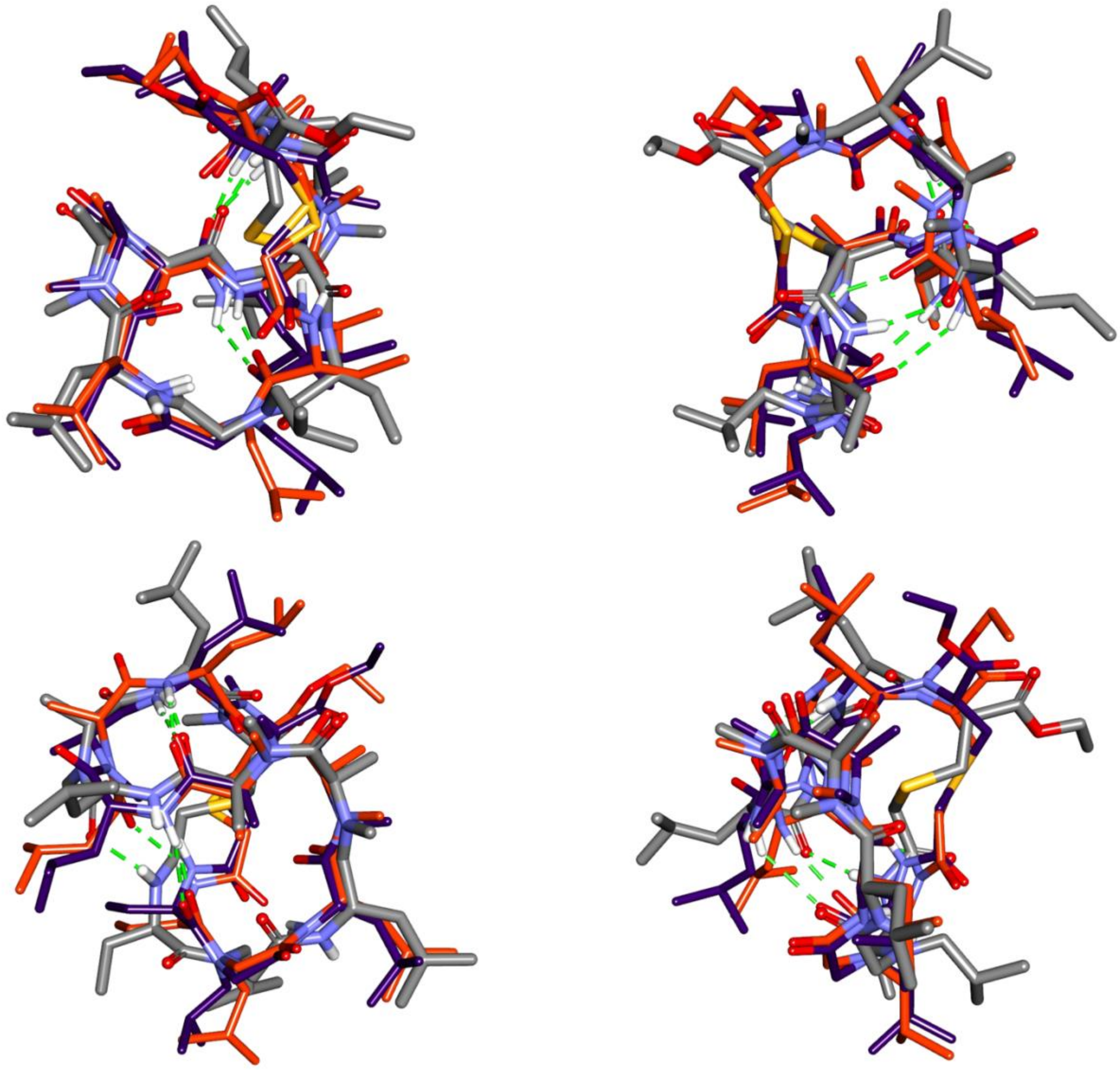

Figure S14: The top conformer of **B1** from the chloroform (purple) and cyclohexane (orange) McMD ensembles overlaid with the predicted NMR structure energy-minimized in chloroform (grey). Hydrogen-bonds are shown as green-dashes. The four pictures are the same 3 overlaid structures shown from different angles.

Figure S15

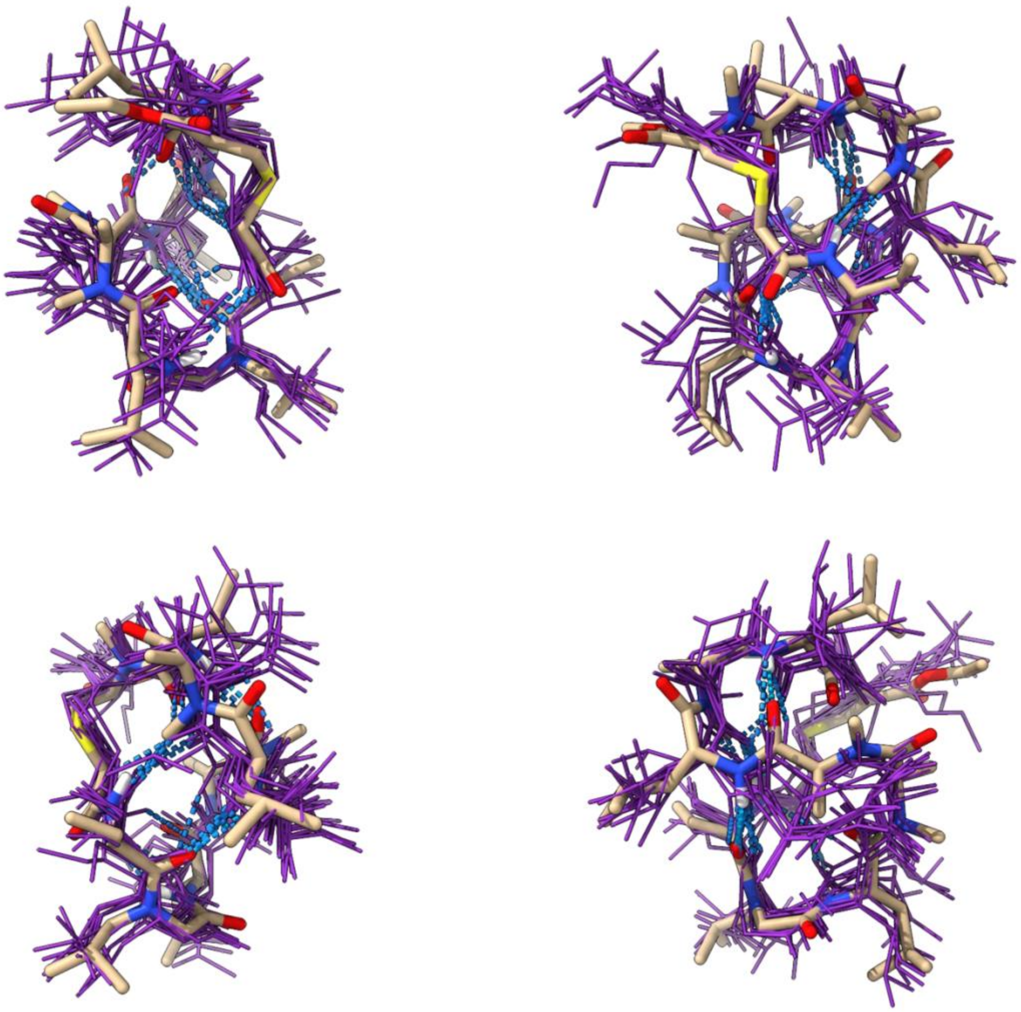

Figure S15: The 10 conformations of **B1** (purple) from the McMD ensemble in chloroform with the lowest RMSD to the calculated NOE distances from the EASY-ROESY spectrum in chloroform. The conformer with the lowest RMSD is colored by atom. Hydrogen-bonds are shown in blue dashes. The four pictures are the same 3 overlaid structures shown from different angles.

Figure S16

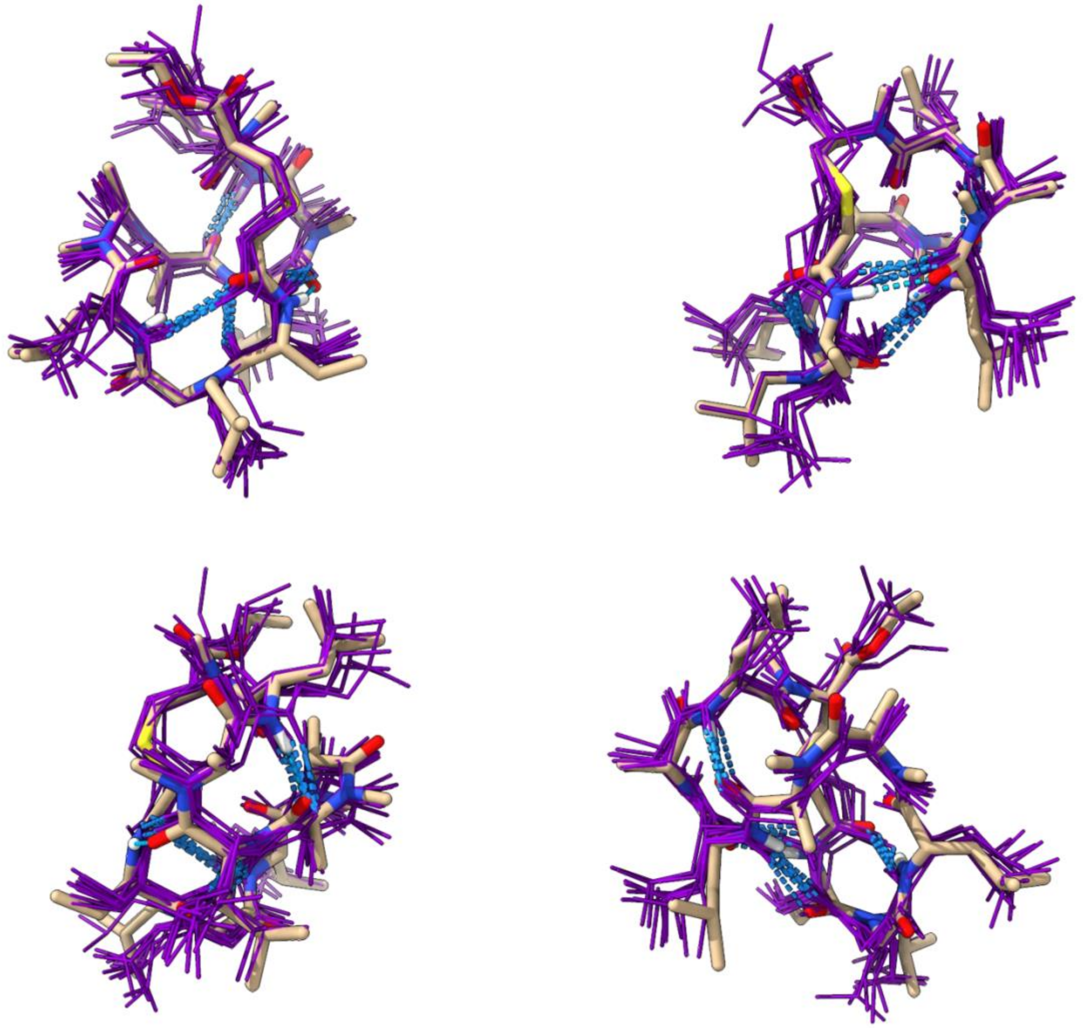

Figure S16: The 10 conformations of B1 (purple) from the McMD ensemble in cyclohexane with the lowest RMSD to the calculated NOE distances from the EASY-ROESY spectrum in chloroform. The conformer with the lowest RMSD is colored by atom. Hydrogen-bonds are shown in blue dashes. The four pictures are the same 3 overlaid structures shown from different angles.

Figure S17

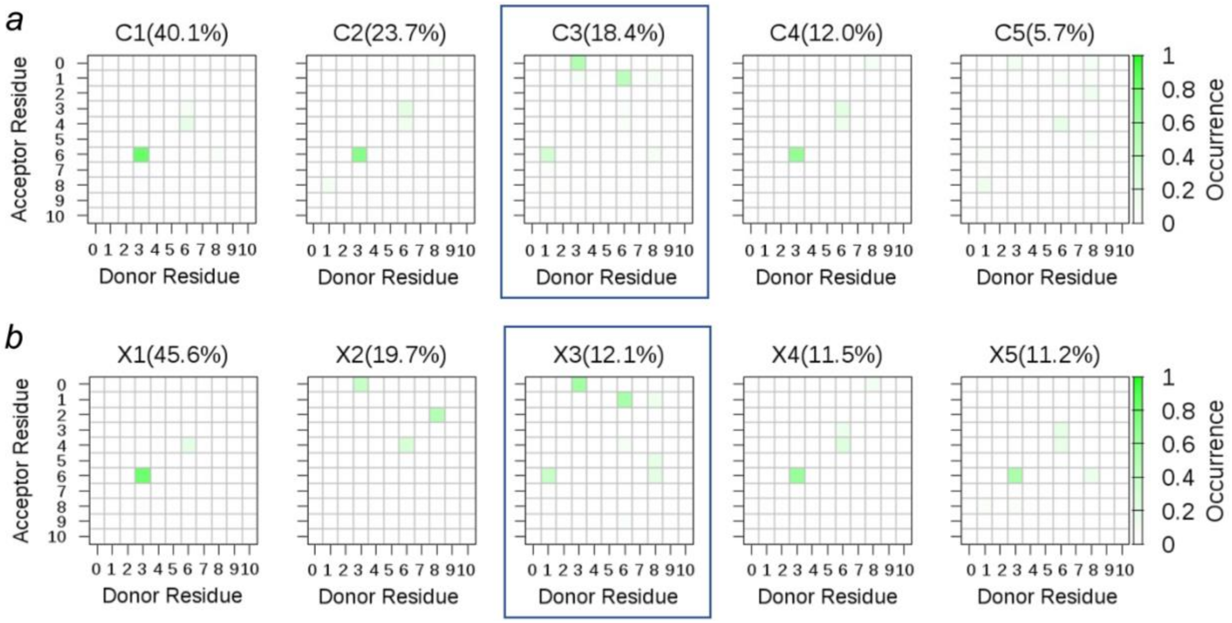

Figure S17: Intermolecular hydrogen-bond maps for **B1** in chloroform and cyclohexane. The lipophilic conformational clusters are boxed in blue. The percentages reflect the portion of the 1000-member conformational ensemble that each plot represents.

Synthetic Procedures

Synthesis of Fmoc-MeCys-OEt

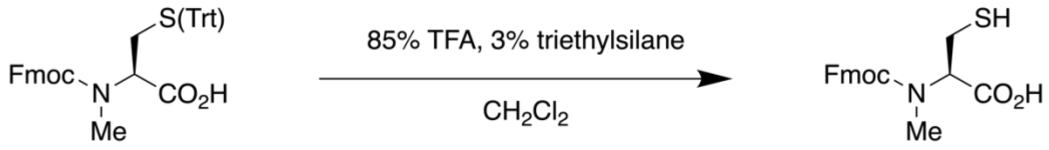

To a 250 mL round-bottomed flask equipped with a stir bar was added Fmoc-MeCys(Trt)-OH (5.92 g, 9.87 mmol) and DCM (24 mL) and allowed to stir at room temperature, open to the air. After complete dissolution, TFA (170 mL) was added, and the solution was capped with a rubber septum before exchanging the atmosphere in the flask with argon. Triethylsilane (6 mL) was added to the solution via syringe and stirring continued at room temperature, under argon for 4 hours at which point the septa was removed and the solvent evaporated overnight under a positive flow of nitrogen. The resulting viscous oil was re-dissolved in DCM and a minimal amount of silica added. The solvent was carefully removed in vacuo, and the remaining silica was loaded into a pre-packed Biotage® SNAP KP-Sil (100 g) cartridge. The product was purified by flash column chromatography [ethyl acetate (w/ 2% acetic acid)/hexane gradient, 30% -> 100% EtOAc over 20 CV] and placed under vacuum to yield a Fmoc-MeCys(SH)-OH as a viscous, slightly-yellow, clear oil (3.12 g, 88%). Purity was assessed by HPLC-MS prior to the next step.

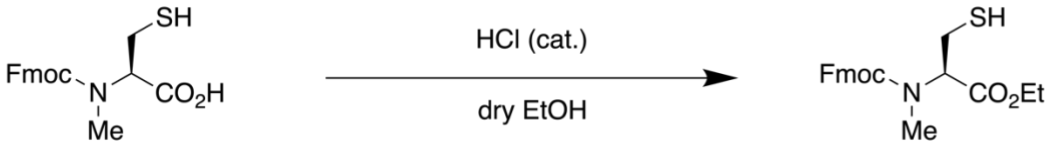

**Procedure modified from method Reported by Diaz-Rodriguez et. al[1, 2]

To a 250 mL round-bottomed flask equipped with a stir bar and charged with Fmoc-MeCys-OH (3.12 g, 8.72 mmol) was added absolute ethanol (~100 mL, pre-dried over 4Å molecular sieves). While stirring, argon was bubbled through the solution via a long syringe needle for 30-60 minutes. After removing the argon needle, 10-15 drops of 37% HCl were added dropwise to the stirring solution. The round-bottomed flask was then equipped with an air condenser, capped at the top with a rubber septum equipped with an argon filled balloon. The solution was then heated to 99 °C, covered in a foil jacket, and allowed to reflux overnight. The next day, the reaction was allowed to cool to room temperature and was then concentrated in vacuo and loaded onto silica. The product was purified by flash column chromatography [ethyl acetate (w/ 2% acetic acid)/hexane gradient, 10% -> 100% EtOAc over 20 CV] and placed under vacuum to yield Fmoc-MeCys-OEt as a viscous, transparent and yellow oil, with a characteristic skunk smell (1.74 g, 45% over two-steps).

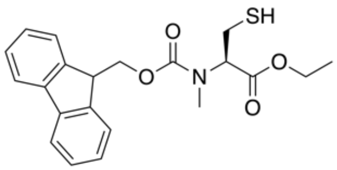

Exact Mass: 385.1348
Molecular Weight: 385.4780
[M+H]⁺ Found: 385.96

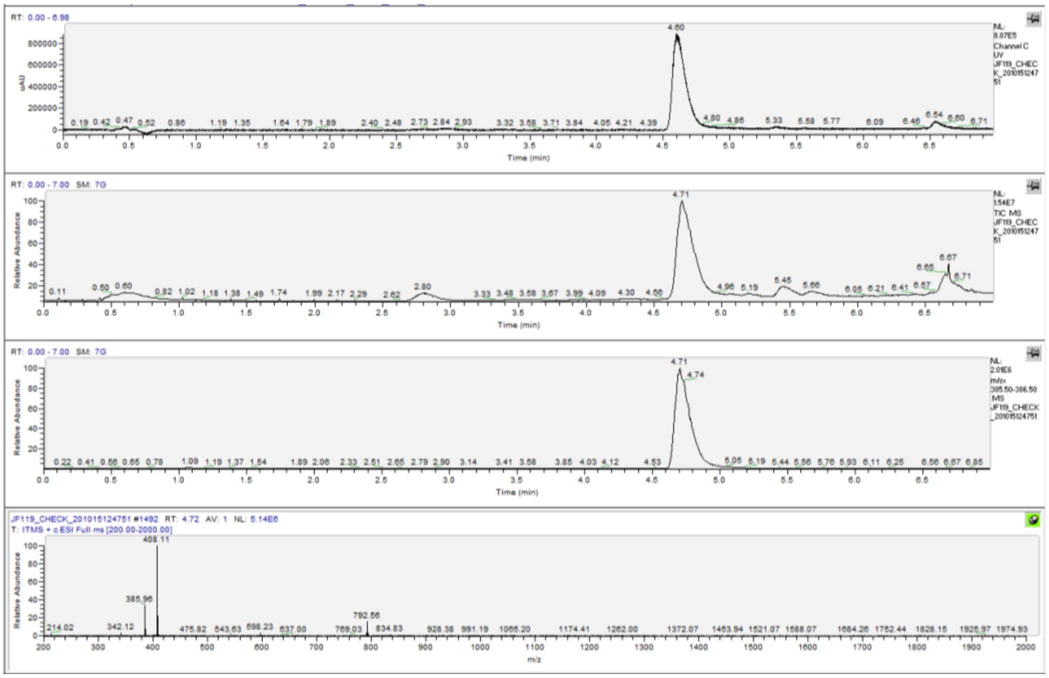

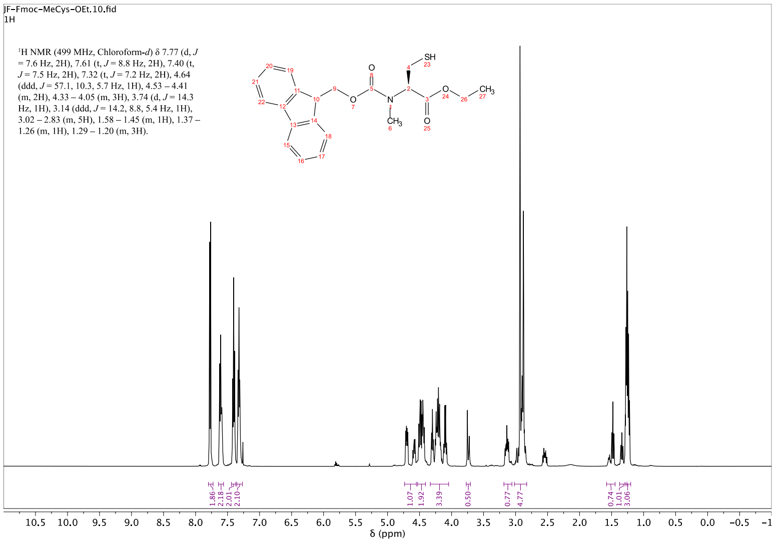

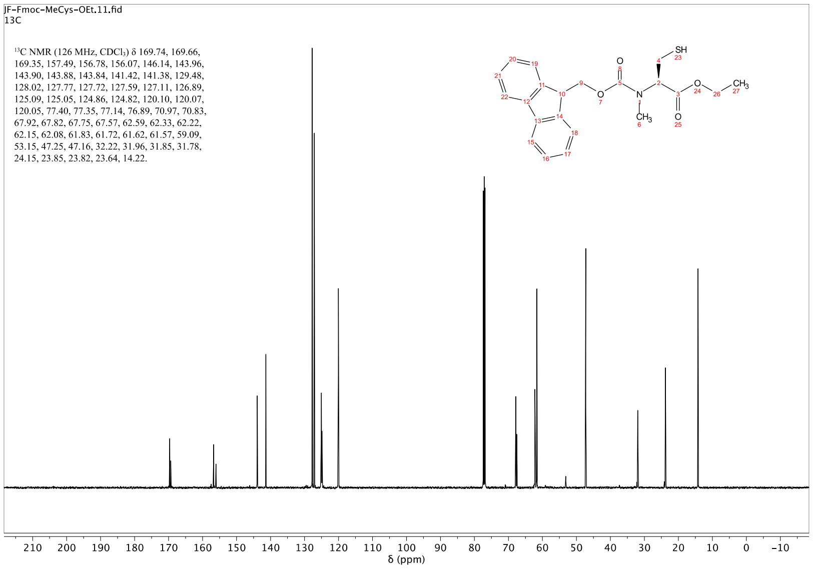

Automated Synthesis

Automated synthesis of linear precursors was accomplished using an automated peptide synthesizer (Prelude X, Protein Technologies). Commercially purchased pre-loaded 2-Chlorotrityl resin (Leucine loaded – 0.65 mmol/g) was used in 0.2 or 0.05 mmol portions. Fmoc deprotections were carried out with 2% DBU and 2% piperidine solution in DMF for 2x15 minutes at room temperature. Couplings were performed using Fmoc-protected amino acids (4 eq), HATU (3.8 eq), and DIPEA (6 eq) in DMF (0.1 M with respect to amino acid) for 1 hour at room temperature. A capping step was performed after each amide coupling with a 1:1 mixture of acetic anhydride and DIPEA in DMF. Each coupling, deprotection, and capping step was followed by a wash with DMF (4x), DCM (3x), and a final wash with DMF (2x). Only amino acid couplings were performed using the automated peptide synthesizer, all peptoid additions, cleavages, and cyclization were carried out manually.

Manual synthesis

Synthesis of linear precursors

Commercially purchased pre-loaded 2-Chlorotrityl resin (Leucine loaded – 0.65 mmol/g) was used in 0.2 mmol portions. Fmoc deprotections were carried out with 2% DBU and 2% piperidine solution in DMF for 15 min. Couplings were performed using Fmoc-protected amino acids (4 eq), HATU (3.8 eq), HOAt (3.8 eq) and DIPEA (6 eq) in DMF (0.1 M with respect to amino acid) for 1 hour at 50 °C or overnight at room temperature. Between coupling and deprotection steps, the resin was washed with DMF (3x), DCM (3x), and one final time with DMF. Coupling reactions were monitored by HPLC-MS and repeated until starting material was no longer observed. The penultimate residue (Fmoc-MeCys-OEt) was added by initial bromoacetylation (as described below in “peptoid addition”) and subsequent formation of the thioether bond. To the bromoacetylated sequence on-resin was added 1 resin volume of DMF and DIPEA (2.5 eq) followed by Fmoc-MeCys-OEt (1.5 eq, dissolved in minimal DMF) and allowed to react for 1.5 hour at 50 °C. The reaction was monitored by HPLC-MS. Prior to resin cleavage, the final Fmoc-deprotection was carried out with 20% piperidine in DMF for 15 min at room temperature. Complete linear peptides were cleaved off resin using 10% TFA in DCM for 1 hour (2x) with a DCM wash equivalent to 2 resin volumes in between repetitions. Solvent was removed under a flow of nitrogen followed by dissolution in acetone and evaporation again under nitrogen. The crude linear product was stored under vacuum until cyclization.

Peptoid addition

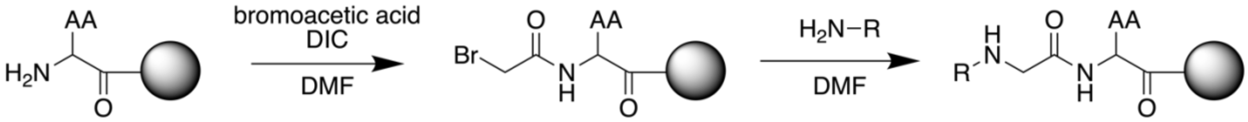

Peptoid residues were added in-sequence to the Fmoc-deprotected amino acid by initial bromoacetylation followed by amine substitution. Bromoacetic acid (1 M) was dissolved in DMF in a separate vessel and followed by addition of DIC (0.5 M). This solution was allowed to rock on a linear shaker for 20 minutes. To the pre-swelled Fmoc-deprotected linear sequence on-resin was added this mixture in sufficient volume to cover the resin and the suspension was allowed to rock at room temperature for 45 min. Upon completion the resin was washed with DMF (3x), DCM (3x), and a final time with DMF. To the resin was then added a 1M solution of the corresponding amine in sieve-dried DMF and allowed to rock overnight at room temperature. The reaction was monitored by HPLC-MS and the amine addition repeated if necessary.

Cyclization
Unpurified linear peptides were dissolved in 10 mL of argon purged ACN with 4 eq of DIPEA and added dropwise to a solution of argon purged ACN containing 2 eq of COMU, for a total concentration of 0.002 M respective to the linear peptide. Reactions were stirred for 12-24 hours under an argon atmosphere until complete cyclization was achieved as monitored by HPLC-MS. The reaction was concentrated in vacuo for purification via flash chromatography to afford the cyclized peptide as a white powder upon lyophilization.

Purification
All crude cyclized peptides were purified using a Biotage Isolera Prime automated chromatography system equipped with a SNAP Bio C18 25g column. The eluting gradient (water/acetonitrile – both containing 0.1% TFA) was modified for each peptide in respect to the observed retention time via HPLC-MS. Libraries were purified by sub-library using Biotage Isolute 103 SPE cartridges with a catch a release method. The resin was swelled with 2CV of ACN and then equilibrated to 10% ACN/water with and additional 2CV wash. The crude sub-library was dissolved in a very minimal amount of DMF prior to loading onto the top of the column. The resin underwent a high-polarity wash (2CV, 10% ACN/water) to remove most impurities/byproducts. The compounds of interest were then eluted and collected with a low-polarity wash (2CV, 100% ACN). All purified compounds/sub-libraries were concentrated under a flow of nitrogen before being redissolved in ACN/H_2O (50:50) and lyophilized to a white/yellow-white powder.

Fmoc-protection of deuterated AA's

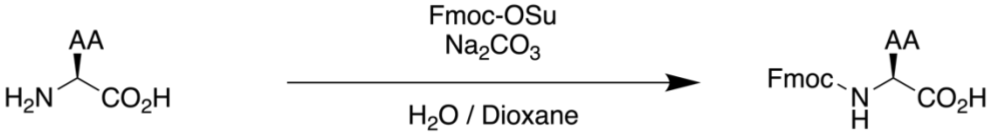

All deuterated amino acids utilized in this study were purchased as the free amino acid and subsequently Fmoc-protected at the N-terminus prior to incorporation within SPPS

To a 100mL round bottom flask charged with a stir bar was added the amino acid (2 mmol) followed by 5mL of aqueous sodium carbonate (0.5 M). The slurry was stirred until complete dissolution of the amino acid. The flask was then placed in an ice bath and allowed to cool while stirring continued. Fmoc-OSu (0.95 eq) was first dissolved in 1,4-dioxane (0.45M) and then added dropwise (~1 drop / 5 sec) to the amino acid solution while stirring over an ice bath was sustained. After complete addition of the Fmoc-OSu solution, the ice bath was removed, and the solution allowed to stir overnight. The solution was then transferred to separatory funnel and diluted with water. This solution was then extracted with diethyl ether (3x), reserving

the aqueous layer, which was acidified with 37% HCl to a pH < 2 (at which point precipitation of the product was observed). The acidified solution was then extracted with ethyl acetate (2-3x) and organic layers collected, combined, and dried over anhydrous magnesium sulfate prior to concentration in vacuo to afford the target Fmoc-protected amino acid as a white powder which was subsequently used without further purification. This reaction procedure was successfully scaled linearly when necessary.

N-methylation of AA's

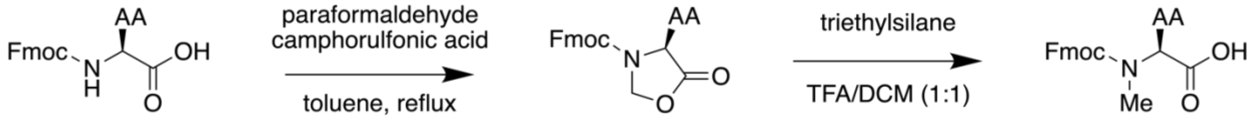

**N-methylated amino acids which were not purchased commercially were prepared in-house via the method below[3] **

To a 250mL round bottom flask charged with a stir bar was added the Fmoc-AA-OH and an equal mass amount of paraformaldehyde. Toluene (75 mL / 5 g AA) was added to the flask and the resulting slurry stirred over a heating plate set to 100 °C. Once the solution reached 100 °C the camphorsulfonic acid (3eq) was added in a single addition and the round bottom flask was then equipped with an air condenser. The solution was kept at 100 °C and allowed to stir for at least 4 hours before removing the air condenser and allowing the solution to concentrate by evaporative loss (~15 mL / 5 g AA). The solution was then allowed to cool and then diluted with ethyl acetate and transferred to a separatory funnel. The organic layer was washed with saturated sodium bicarbonate (2x) and brine (NaCl, 1x), dried over anhydrous magnesium sulfate and concentrated in vacuo to a viscous oil. The oil was resuspended in a minimal amount of DCM (~8 mL / 5 g AA) and stirred at room temperature while an equal volume of TFA was added to the round bottom flask. The flask was capped with a rubber septum and the solution kept stirring at room temperature for 5 minutes; at which point triethylsilane (3eq) was added via syringe. The solution was kept stirring at room temperature overnight. The solution was then concentrated under a flow of air before being resuspended in ethyl acetate and transferred to a separatory funnel. The organic layer was washed with water (2x) and brine (NaCl, 1x), dried over anhydrous magnesium sulfate, and concentrated in vacuo to yield a viscous oil. The crude product was recrystallized from hexanes/ethyl acetate and the crystals isolated, washed with cold hexanes and dried under vacuum to afford the target N-methylated Fmoc-AA as a white crystalline solid. This reaction was scaled and run comfortably at 1g, 5g, and 10g scales.

Analytical Procedures

NMR analysis

General NMR purity spectra were recorded on a Bruker Avance III HD 500 MHz equipped with a Smart Probe in $CDCl_3$. Deeper structural analysis was performed on a Bruker Avance III HD 800 MHz equipped with a TCI cryoprobe in $CDCl_3$. NMR spectra were processed and analyzed with MestReNova software (version 12.0.0-20080)

UPLC-MS analysis

All UPLC-MS analysis was performed on a Thermo Scientific Ultimate 3000 UPLC system equipped with a Thermo Scientific OrbiTrap Velos Pro mass spectrometer. PAMPA mixture analysis was used performed with a Thermo Hypersil GOLD C18 (30 mm x 2.1 mm, 1.9 μm particle size) column. Purity analysis was performed on a Thermo Scientific Acclaim RSLC 120 C18 (2.1 mm x 250 mm, 2.2 mm, 120 Å) column. All samples were run eluting in water/acetonitrile with 0.1% formic acid. Purity assessment was quantified by UV absorbance at 200 nm.

PAMPA Mixture conditions

Flow rate: 1.0 mL/min
Temperature: 50 ºC
Gradient: 0-0.5min (05%ACN), 0.5-7min (20-100%ACN), 7-9min (100%ACN), 9-10min (05%ACN)
Source ionization: off
MS mode: FTMS
Resolution: 30000
Polarity: (+)
Data type: centroid
MS^n: Collision-induced dissociation
Normalized collision energy: 35.0
Isolation width: 1.0 m/z
Activation Q: 0.250
Activation time: 10.00 ms
UV: off

PAMPA Individual conditions

Flow rate: 0.4 mL/min
Temperature: 50 ºC
Gradient: 0-2min (10%ACN), 2-22min (30-100%ACN), 22-27min (100%ACN), 27-30min (10%ACN)
Source ionization: off
MS mode: FTMS
Resolution: 30000
Polarity: (+)
Data type: centroid
MS^n: Collision-induced dissociation
Normalized collision energy: 35.0
Isolation width: 1.0 m/z
Activation Q: 0.250
Activation time: 10.00 ms
UV: off

Purity Assessment conditions

Flow rate: 0.4 mL/min
Temperature: 50 ºC
Gradient: 0-20min (05-100%ACN), 20-25min (100%ACN), 25-30min (05%ACN)
Source ionization: off
MS mode: FTMS
Resolution: 30000
Polarity: (+)
Data type: centroid
MS^n: Collision-induced dissociation
Normalized collision energy: 35.0
Isolation width: 1.0 m/z
Activation Q: 0.250
Activation time: 10.00 ms
Scan Range: 200-2000 m/z
UV: Monitoring at 200, 220, 260, and 280 nm

PAMPA

Primary protocol

Protocol is identical for both mixture and pure compound analysis.

The utilized PAMPA procedure and subsequent data analysis protocol has been previously described in detail by our lab.[4]

Prior to analysis, the mixtures of interest were prepared as DMSO stocks at a concentration of 250 µM per individual compound. Two of the following: Carbamazepine (CBZ), propranolol (Prop), and 1NMe3[5] were included as internal standards and prepared as DMSO stocks at a concentration of 2000 µM. The PAMPA assay was carried out with a 96-well donor plate with 0.45 µ hydrophobic Immobilon-P membrane supports (Millipore MAIPNTR10) and a 96-well Teflon acceptor plate (Millipore MSSACCEPTOR). The PAMPA protocol then proceeded as follows:

1. Donor well solutions were prepared to a volume of 1000 µL with the analytes as a solution in 1X PBS pH 7.4 and 5% DMSO. To a 3 mL Eppendorf tube was added 950 µL of 1X PBS pH 7.4, 40 µL of the mixture of interest (250 µM/cmpd in DMSO), and 5 µL each of both CBZ and 1NMe3 (2000 µM in DMSO). The solutions were vortexed and set aside.
    a. Assay concentrations
        i. Analyte – 10 µM per compound
        ii. Carbamazepine / 1NMe3 / Propranolol – 10 µM each (only 2 of the 3 were chosen)
        iii. 5% DMSO
2. A 1% solution of soy lecithin in dodecane was freshly prepared in a 3 mL Eppendorf tube and sonicated (5 min) to ensure full dissolution of the lecithin. The donor membrane plate was turned upside down and bottom of the membranes exposed. 5 µL of the 1% soy lecithin solution in dodecane was then placed on the bottom of the membrane, allowing the drop to spread across the full area of the membrane. Any membranes with visible damage were not used in the assay. The membranes were allowed to equilibrate for 5 min before loading.
3. Each well within the Teflon block acceptor plate that was to be used in the assay was then loaded with 300 µL of 1X PBS pH 7.4 and 5% DMSO.
4. Working without break, 150 µL of each donor well solution was transferred from the to the 96-well donor plate. Each mixture of interest was run in quadruplicate, using a new well for each replicate.
5. As soon as the 96-well donor plate was finished being loaded, it was gently placed into the 96-well Teflon acceptor plate, taking care not to trap any air bubbles beneath the membrane.
6. The donor/acceptor plates were covered gently with the lid to minimize evaporation and transferred gently as a system to a sealed chamber and left to sit overnight at room temperature. The chamber was kept from drying by placing a wet paper towel within to prevent evaporation.
7. After ~19-20 hours the donor plate was removed from the acceptor plate. The exact time was marked to the second.
8. 50 µL of each well (donor and acceptor) were transferred to a 96-well plate and diluted with 50 µL of methanol prior to heat sealing and quantification by UPLC-MS.

Alternative PAMPA Assay

A modified PAMPA was used for compound permeability measurements and was performed by Pharmaron. Briefly, alkyne stock solution (1 mM in DMSO) was diluted (50 µM final in PBS, pH 7.4). The lipid solution (1.8% w/v egg lecithin in dodecane, 5 µL spotting volume) was added to each acceptor well of the multiscreen-IP filter plate. 300 µL PBS was added to all wells of the acceptor plate and diluted alkyne stock (300 µL) was added to the wells of the donor plate in triplicate. The plate was assembled and incubated for 16 h at 37 °C. An aliquot of donor well sample (2.5 µL) was diluted in PBS (47.5 µL) and an aliquot of the

acceptor well (50 µL) was transferred to a 96-well analysis plate. Internal standard (100 nM alprazolam, 200 nM caffeine, 200 nM diclofenac in 100% MeOH) was added to all samples. Samples were vortexed and centrifuged (20 min, 3,220 × g), then analyzed by LC-MS/MS.

The effective permeability (Pe) was calculated as:

$$Log\ P_e = Log\ \left\{ C\times\left[-Ln\ \left(1-\frac{[drug]_{acceptor}}{[drug]_{equilibrium}}\right)\right]\right\}$$

$$C = VD \times VA / [(VD + VA) \times t \times A]$$

Where VD is the donor compartment volume (0.3 mL), VA is the acceptor compartment volume (0.3 mL), A is the filter area (0.24 cm^2), and t is the time (16 h).

gMDCK Permeability Assay

The gMDCKI cell line (overexpressed cell line of Madin-Darby Canine Kidney which acquired from the NIH) maintained in DMEM medium supplemented with 1% pen-strep, 5 µg/mL Plasmocin, and 10% FBS under standard culture conditions (37 ºC, 95% humidity, and 5% CO_2), were seeded (0.75 x 10^5 cells) on the HTS Transwell-96 permeable support (Corning) to form a confluent monolayer for two days.

All experimental steps were fully automated on Hamilton Vantage. Compound stock solution (1 mM in DMSO) was diluted (10 µM final in HBSS, pH 7.4) with Lucifer Yellow as leakage indicator (20 µM). After washing with HBSS buffer, the solution of test compounds was added to the apical side of the monolayers. The plate was incubated for 3 h at 37 ºC. T_0 samples (10 µL of the 10 µM compounds diluted solution with LY) were transferred to a 96-well plate, and then 40 µL of HBSS was added. After 3 hours, 50 µL basolateral samples in the receiving plate were transferred to a fresh 96-well plate and added 100 µL of IS solution (Propranolol). LY were read by plate reader and the samples were analyzed by LC/MS/MS.

The apparent permeability coefficient (P_{app}) is calculated from the permeation rate and compound concentration at t=0h and t=3h. Where:

$$P_{app} = \frac{dQ}{dt} x \frac{1}{A \times C_0}$$

dQ/dt: amount of product present in the basal (A-B) or apical (B-A) compartment as a function of time (nmol/s).
A: area of transwell (cm^2).
C_0: initial concentration of product applied in the apical (A-B) or basal (B-A) compartment (nmol/ml).

Kinetic Solubility PBS Protocol

4 µL of 10 mM compound in DMSO stock was added to 196 µL 1 X PBS pH 7.4 in a 96-well microplate to get a final concentration of 200 µM to shake for 24 hours at 1000 rpm. The sample was filtered sample using a 96-Multiscreen HTS filter plate (Millipore MSSLBPC50) and a positive pressure manifold. 100 µL

of the filtered sample was transferred into a microplate filled with 100 µL DMSO (1:1 dilution). Samples were quantified using a charged aerosol detector (CAD) attached to a UHPLC-UV-MS system (Agilent 1290 Infinity II, MSDiQ, ThermoFisher Corona Veo CAD 5081.0010) and with a set of calibration standards for CAD. Agilent Poroshell 120 EC-C18 column (1.9 µm, 50x2.1 mm) was utilized with mobile phases A (0.1% formic acid in water) and mobile phase B (methanol with 0.1% formic acid). A 4 minute gradient method was used with the following steps: 2-98% B over 2.5 min, hold at 98% for 0.5 min, 2% B for 1 min. Additionally the inverse gradient was utilized to maintain a 1:1 mixture of mobile phases A:B before introduction into the CAD.

Circular Dichroism

Spectra were measured from 180 to 320 nm using a CD spectrophotometer (J-1500 JASCO Inc., Easton, MD). Sample and solvent spectra were collected using a 1 mm path length quartz cuvette with a total of x sets of data measured for each of the samples. Data were collected every 0.1 nm with a 4 nm bandwidth using a digital integration time of 4 sec and a scan speed of 50 nm/min. A solvent blank was subtracted from each sample spectrum. Compounds were analyzed at 50 uM concentration.

McMD protocol

System Setup

The initial conformer was built using the Molecular Operating Environment (MOE)[6] protein builder, and LowModeMD implemented in MOE was used to search for the most stable conformer in vacuo. PACKMOL[7] was used to solvate the system, where the numbers of water, chloroform, and cyclohexane molecules were 2500, 500, and 300, respectively. The AMBER ff03 force field was used for amino acids[8] and ForceField_NCAA was used for N-methylated alanine and Abu.[9] The angle parameter c-c3-ss was adopted for MeCys residue from the general amber force field (GAFF).[10] For the atomic charges of peptoid residue and tail CO_2Et, RESP ESP charges from R.E.D. version III.52[11] were used. The initially solvated system was minimized by steepest descent, followed by 50,000 steps of NVT calculation. The system was then equilibrated by NPT calculation at atmospheric pressure by applying the Berendsen barostat for 500,000 steps. A positional restraint was imposed on $C\alpha$ atoms during equilibration. The resultant system was used as an initial structure for the following simulations.

Simulation details.

Virtual-system-coupled Trivial Trajectory Parallelization of Multicanonical Molecular Dynamics (TTP-V-McMD)[12, 13] simulations were adopted to effectively sample conformations of cyclic peptides. A total of 288 pre-TTP-V-McMD runs were initiated with random velocities for each atom at T = 300 K. Then the systems were heated to T = 1525 K with 5,000 steps, followed by 495,000 steps at T = 1525 K to randomize the initial structures. Flat potential energy distributions between T=280 K and 1525 K were obtained by iterating TTP-V-McMD simulations for ten times with eight virtual states. The cut-off distance for Coulombic and van der Waals interactions was 1.0 nm, and PME was used to calculate the long-range electrostatic interaction. The NVT ensemble was used for all TTP-V-McMD simulations using the velocity rescaling method (Bussi thermostat).[14] The LINCS algorithm was used to constrain the bonds with a hydrogen atom, enabling a time step of 2.0 fs. For each solvent, 1×10^7 steps × 288 production runs (aggregating 5.76 µs) were performed. The structure and potential energy were stored every 2 ps. The virtual states were exchanged in every 5,000 steps. All 2.88 million conformers were considered to determine the free energy landscape by a potential of mean force (PMF) calculation; $W = -k_BT \ln \rho$, where k_B is the Boltzmann constant, T = 300 K and ρ is the density of state. An in-house implemented TTP-V-McMD using GROMACS version 5.1.4 was used for the simulations.

Individual compound validation of mixtures by MS

Liposcan

Group A1

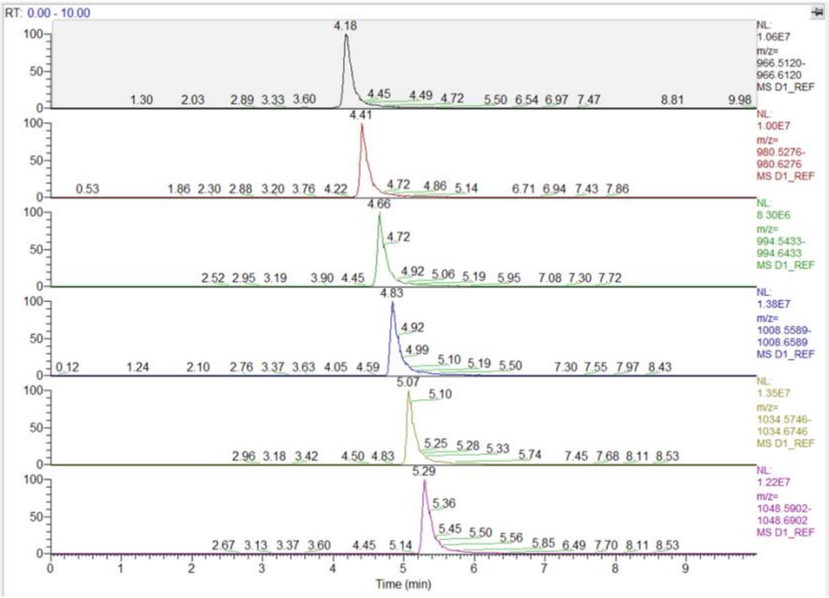

Group A2

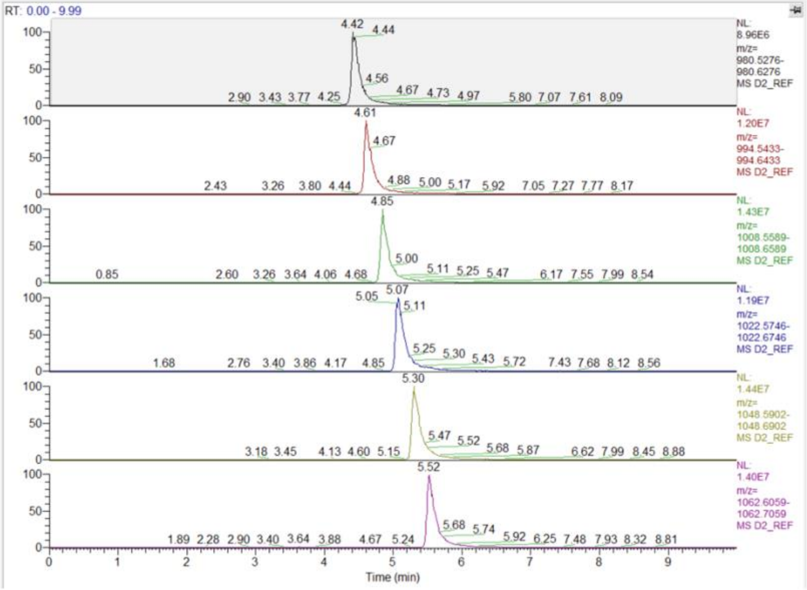

Group B1

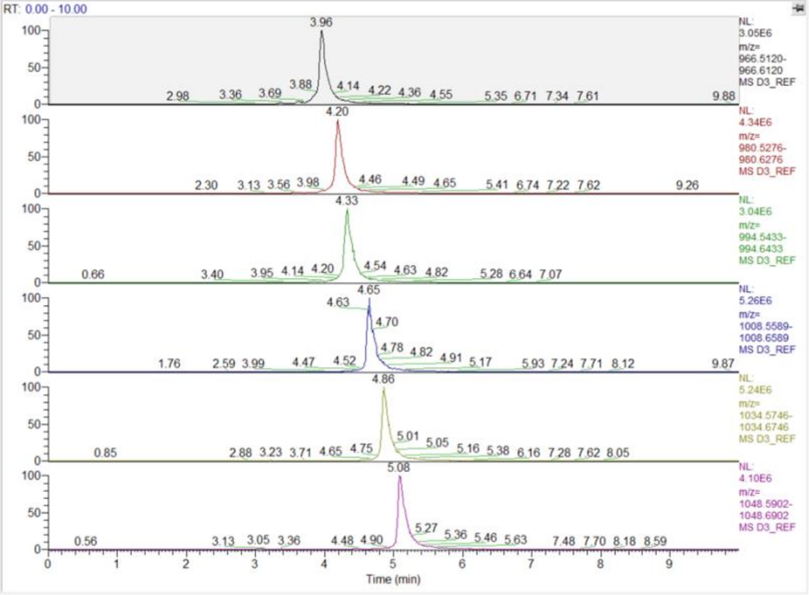

Group B2

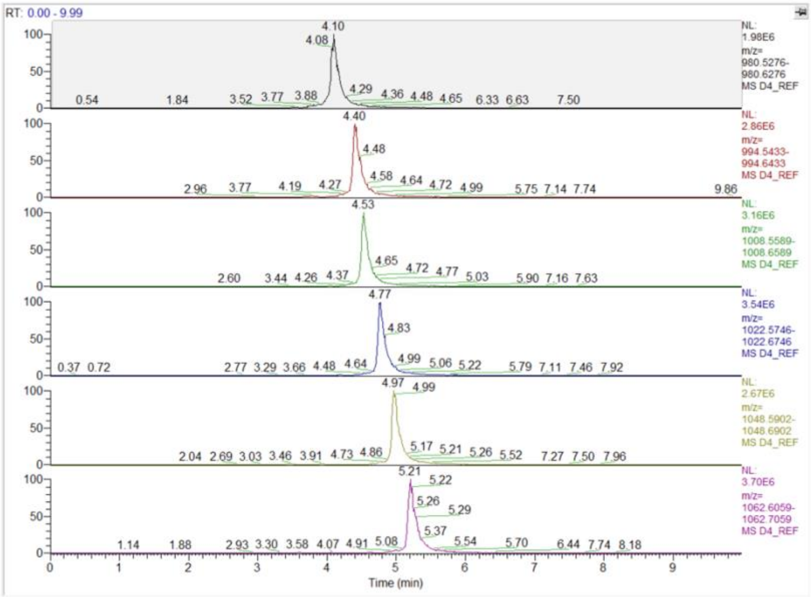

Group C1

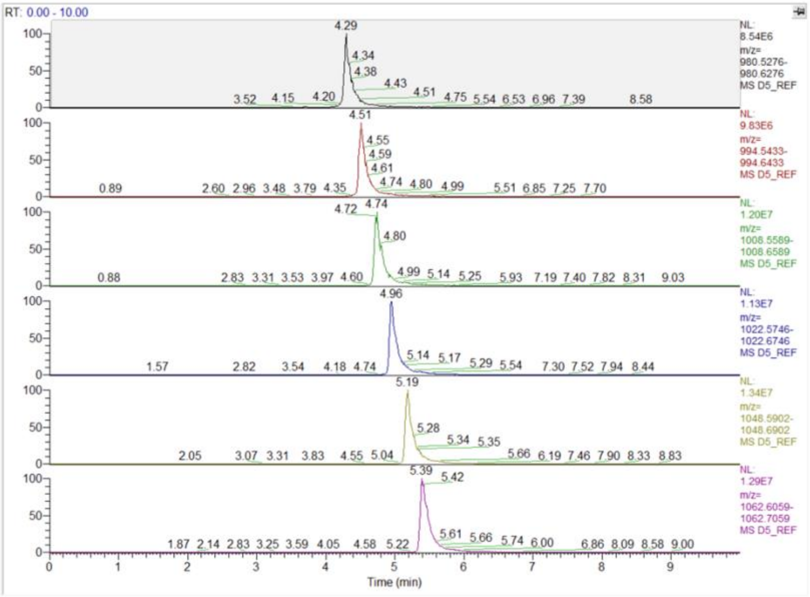

Group C2

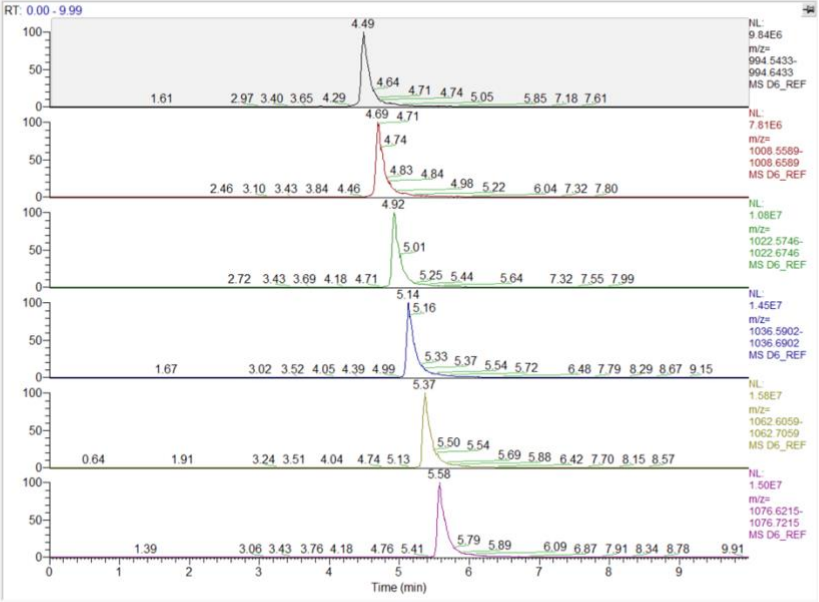

Stereoscan

Group ALL

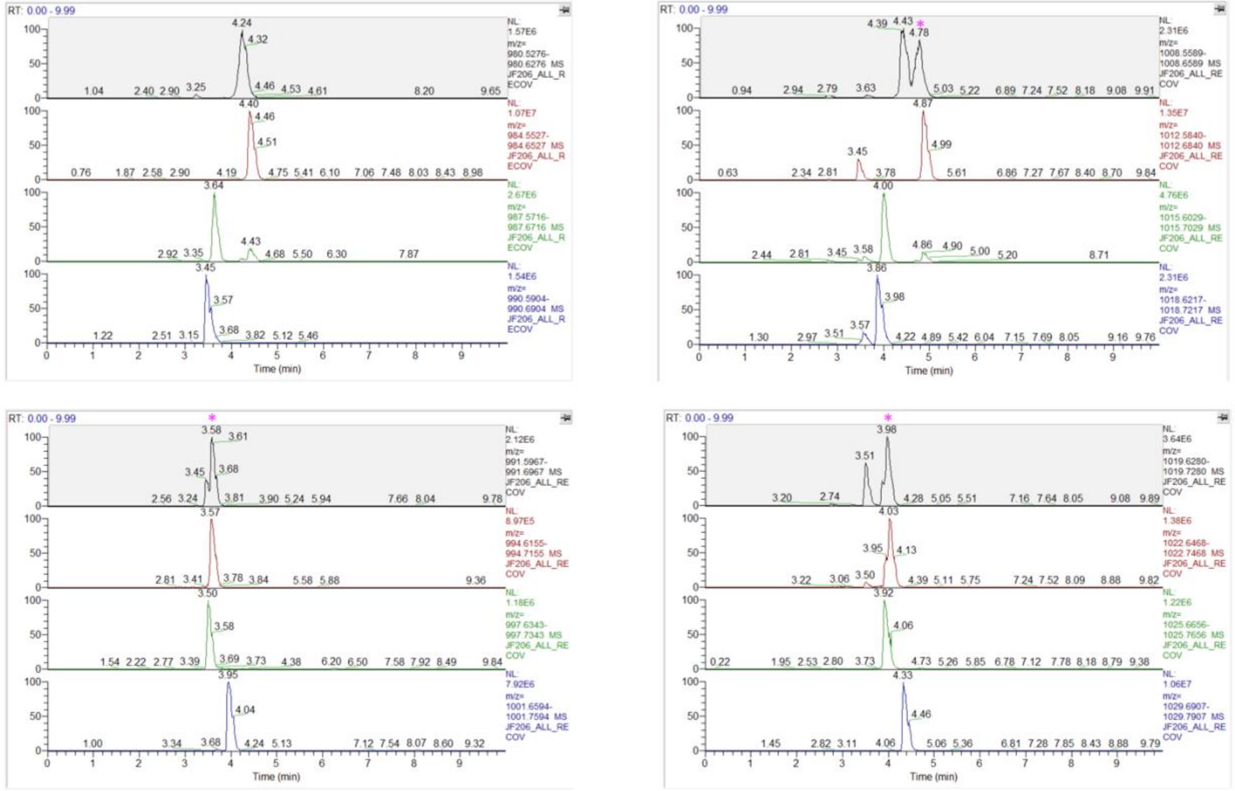

Group ALD

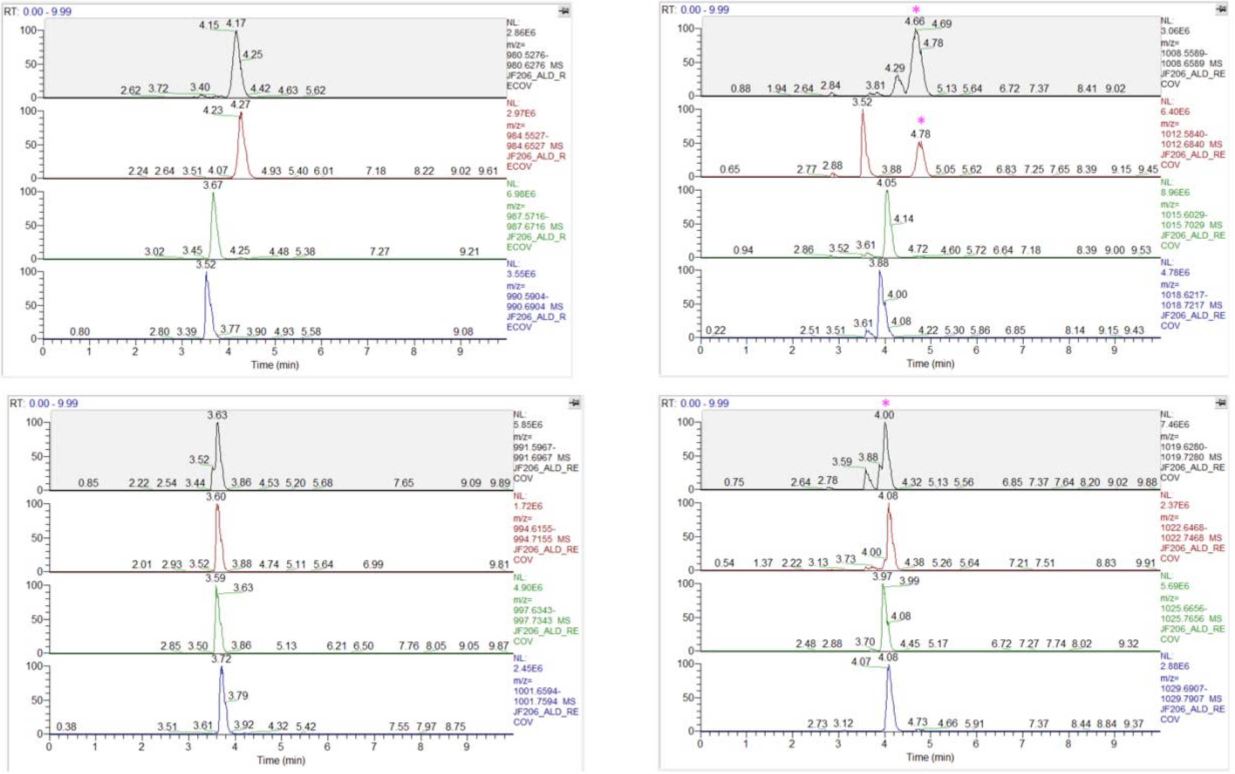

Group ADL

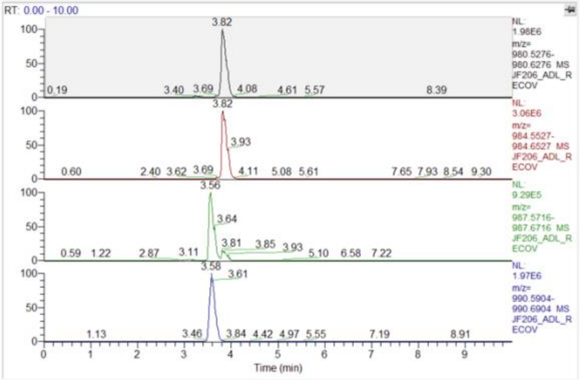

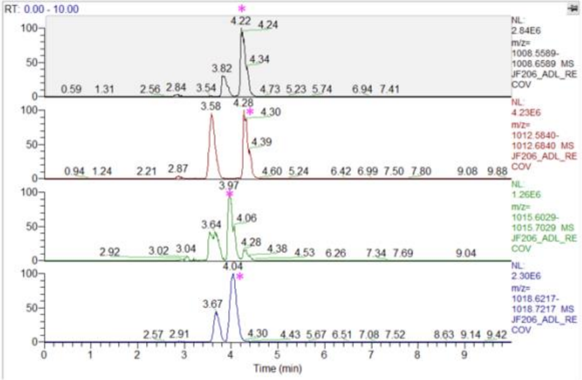

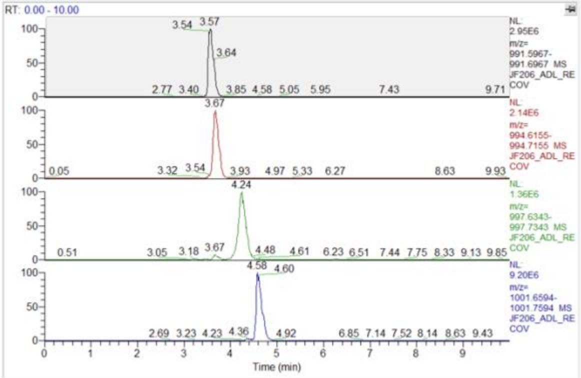

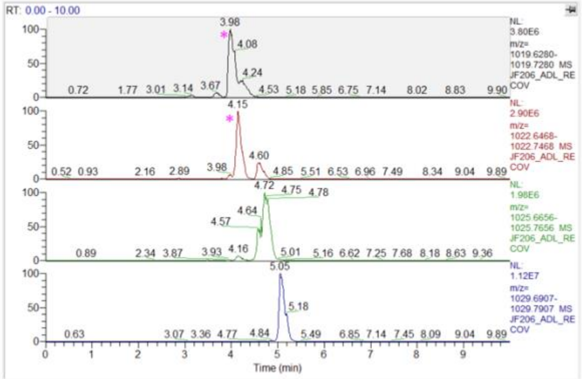

Group ADD

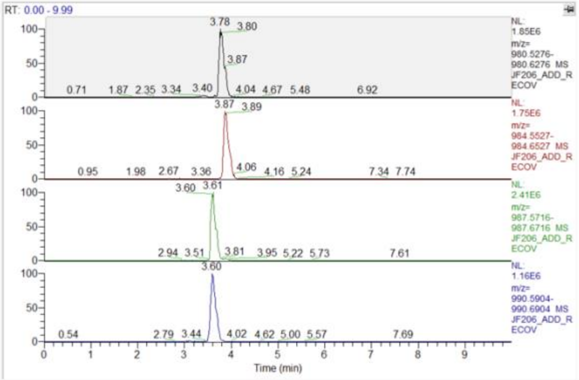

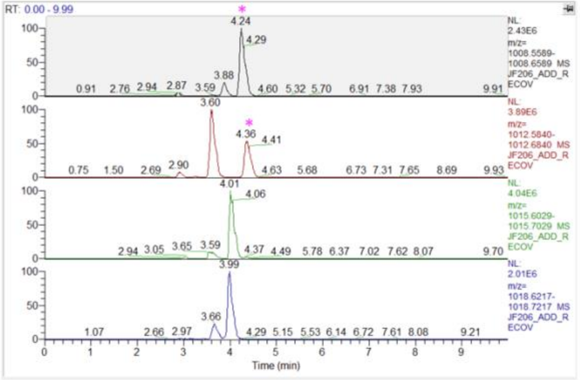

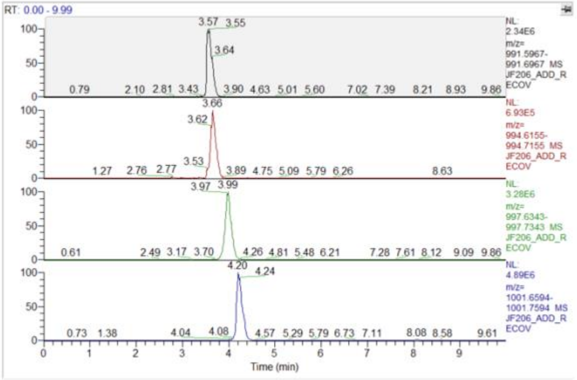

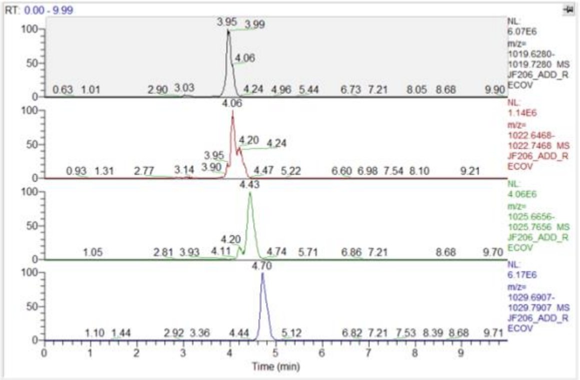

Group A2L

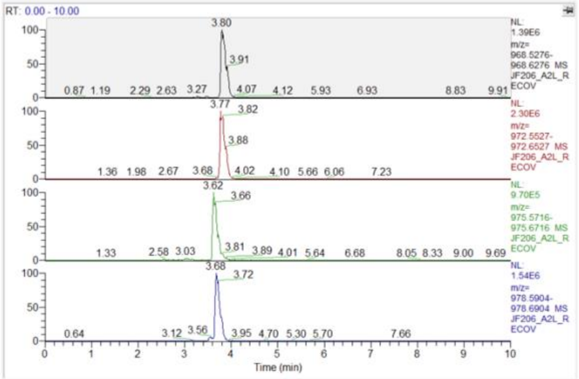

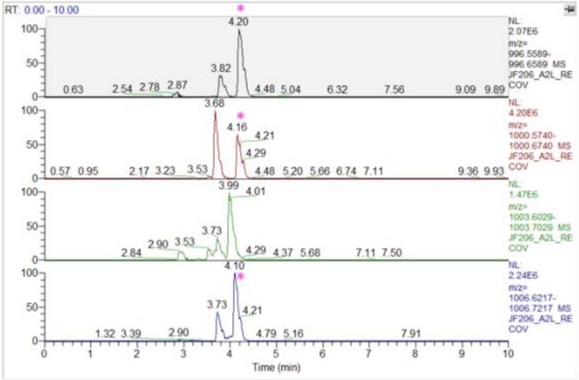

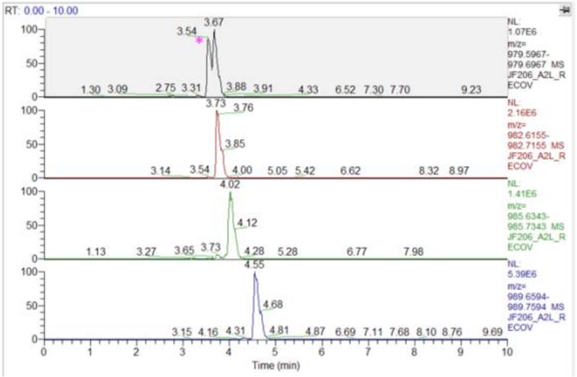

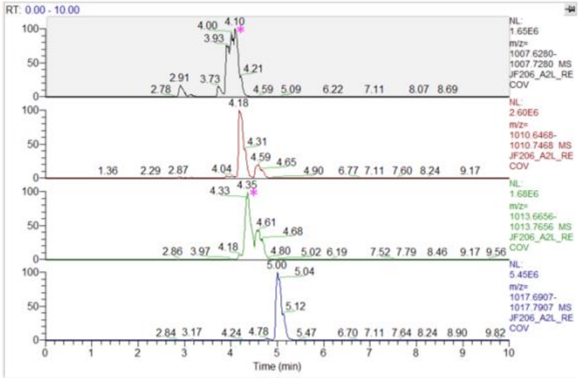

Group A2D

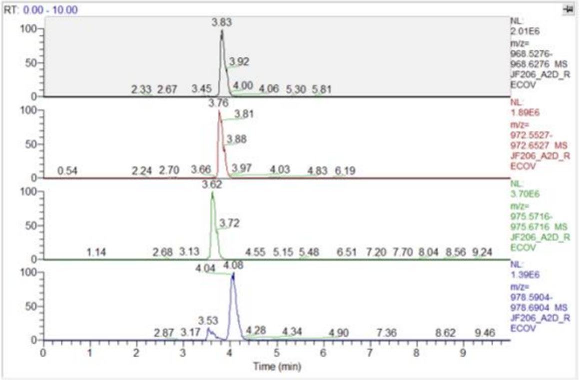

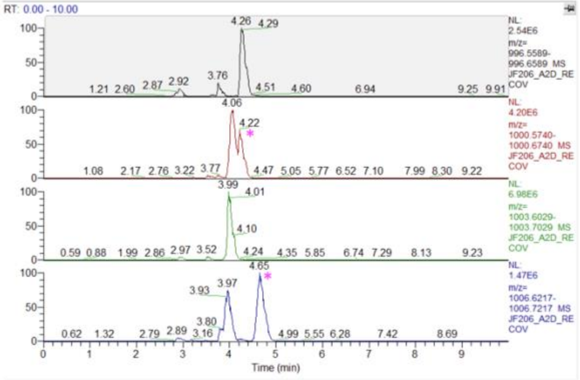

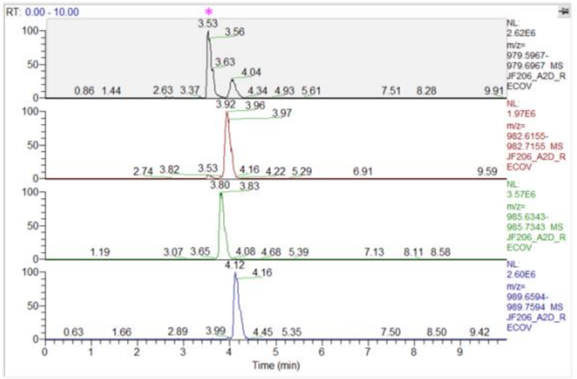

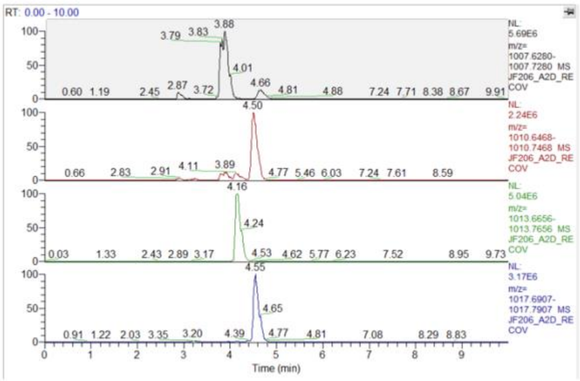

Group BLL

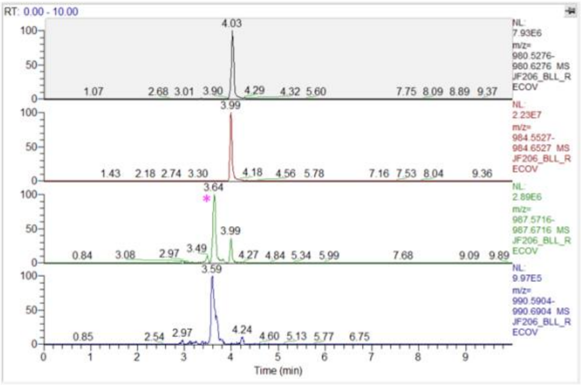

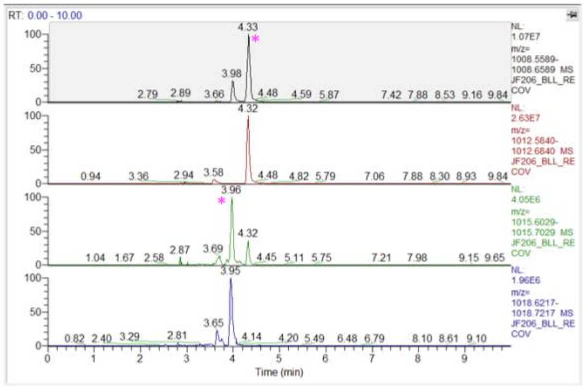

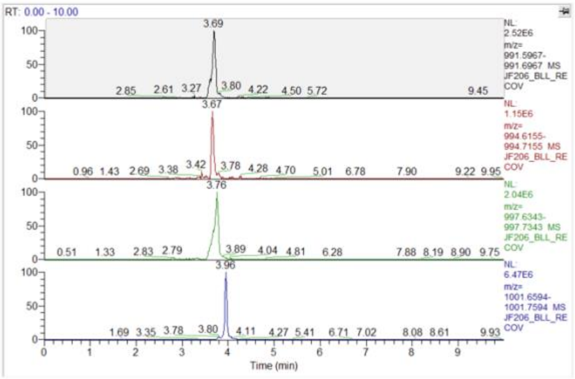

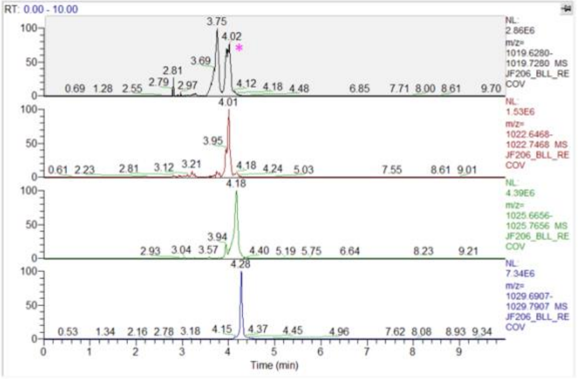

Group BLD

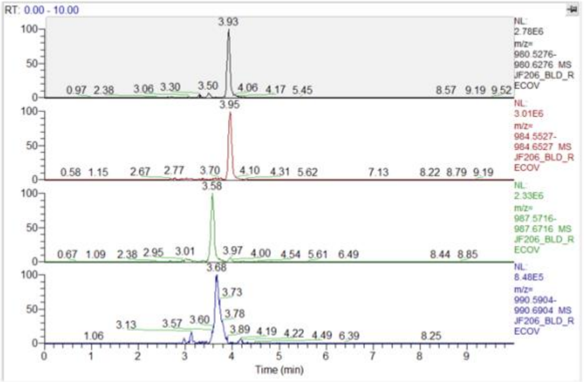

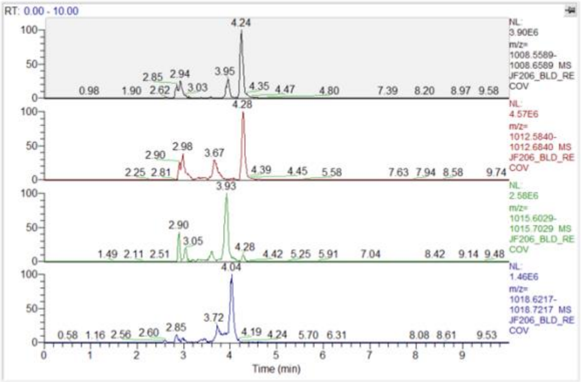

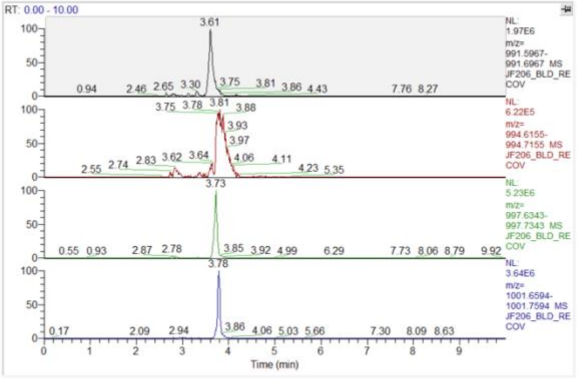

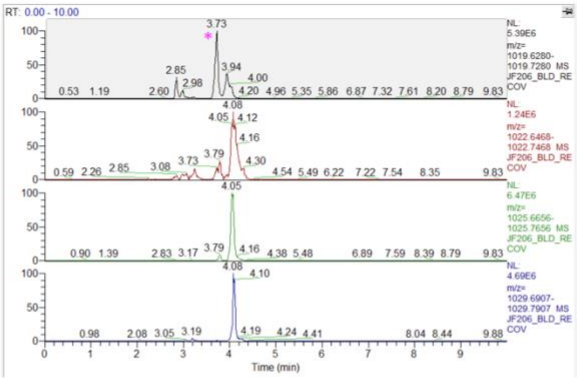

Group BDL

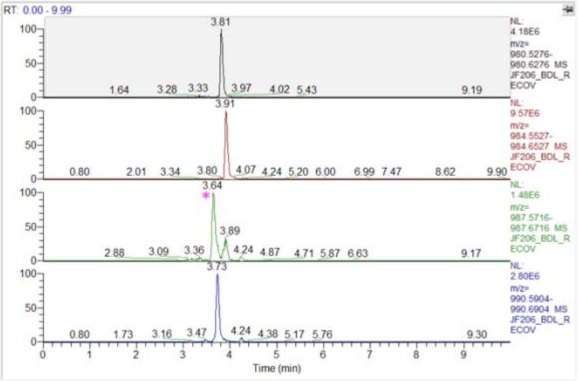

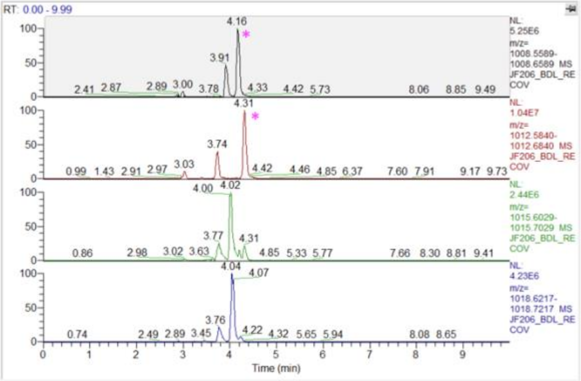

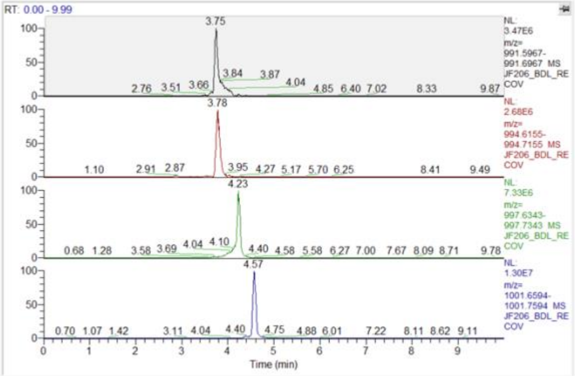

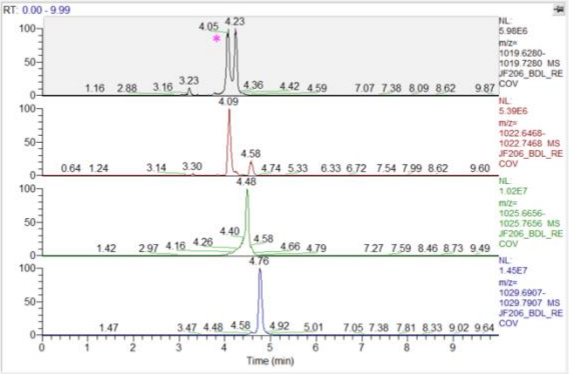

Group BDD

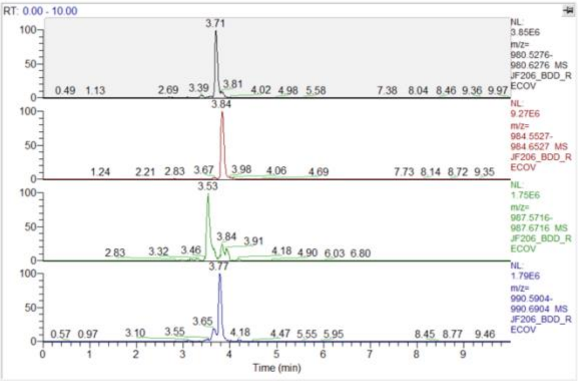

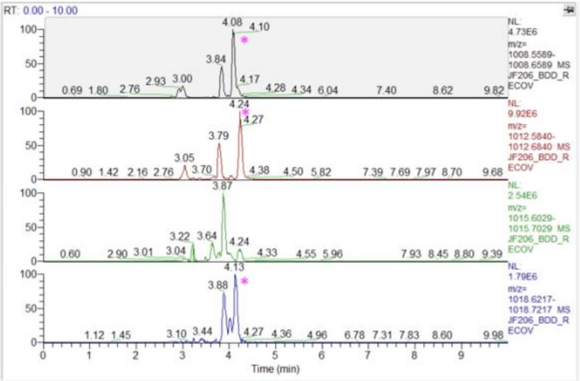

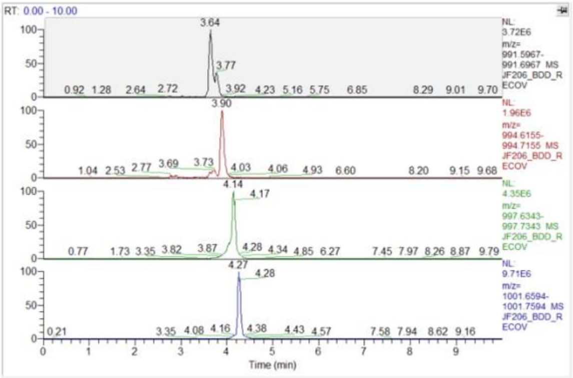

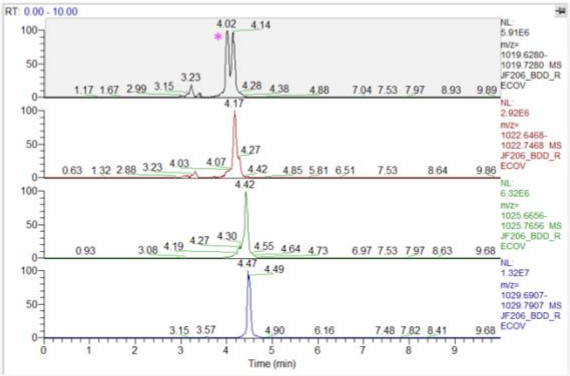

Group B2L

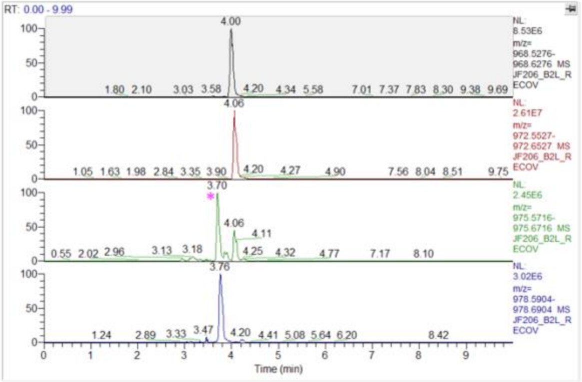

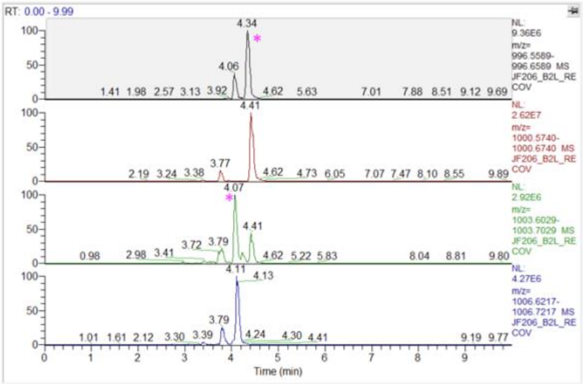

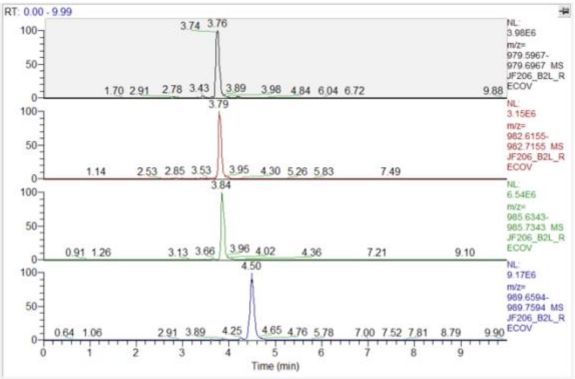

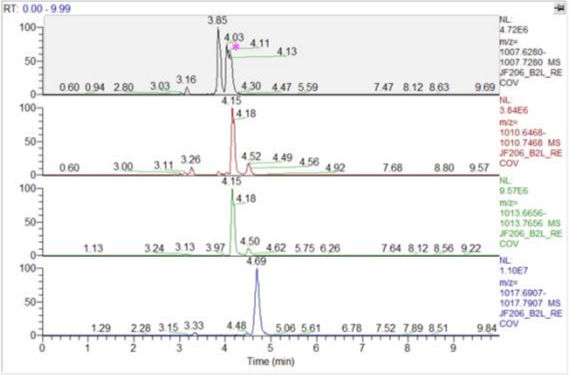

Group B2D

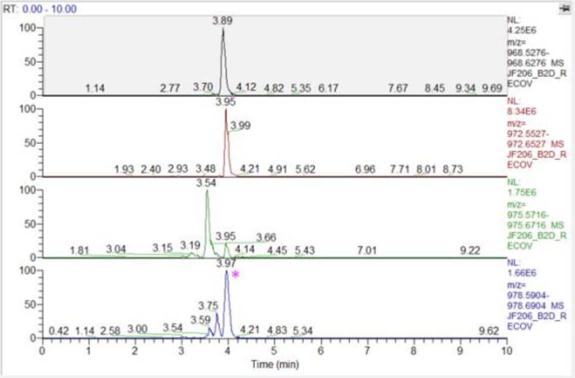

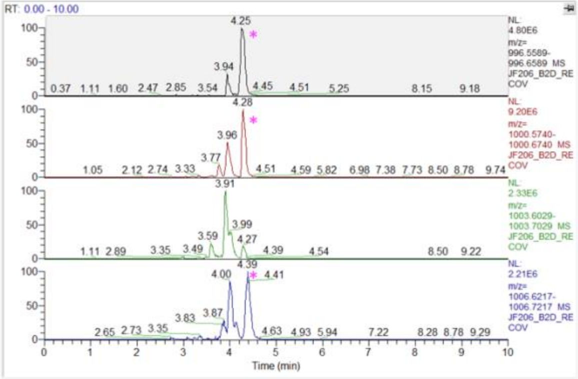

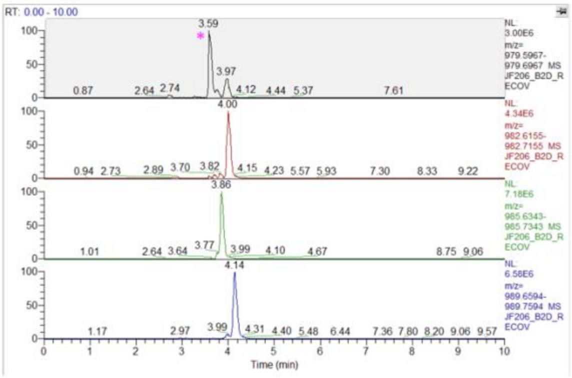

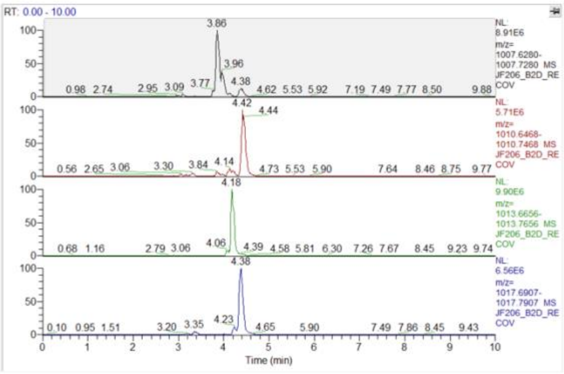

Solution Structure Analysis of **B1**

1D/2D NMR Spectra

^{1}H NMR (δ 9.0-5.5)

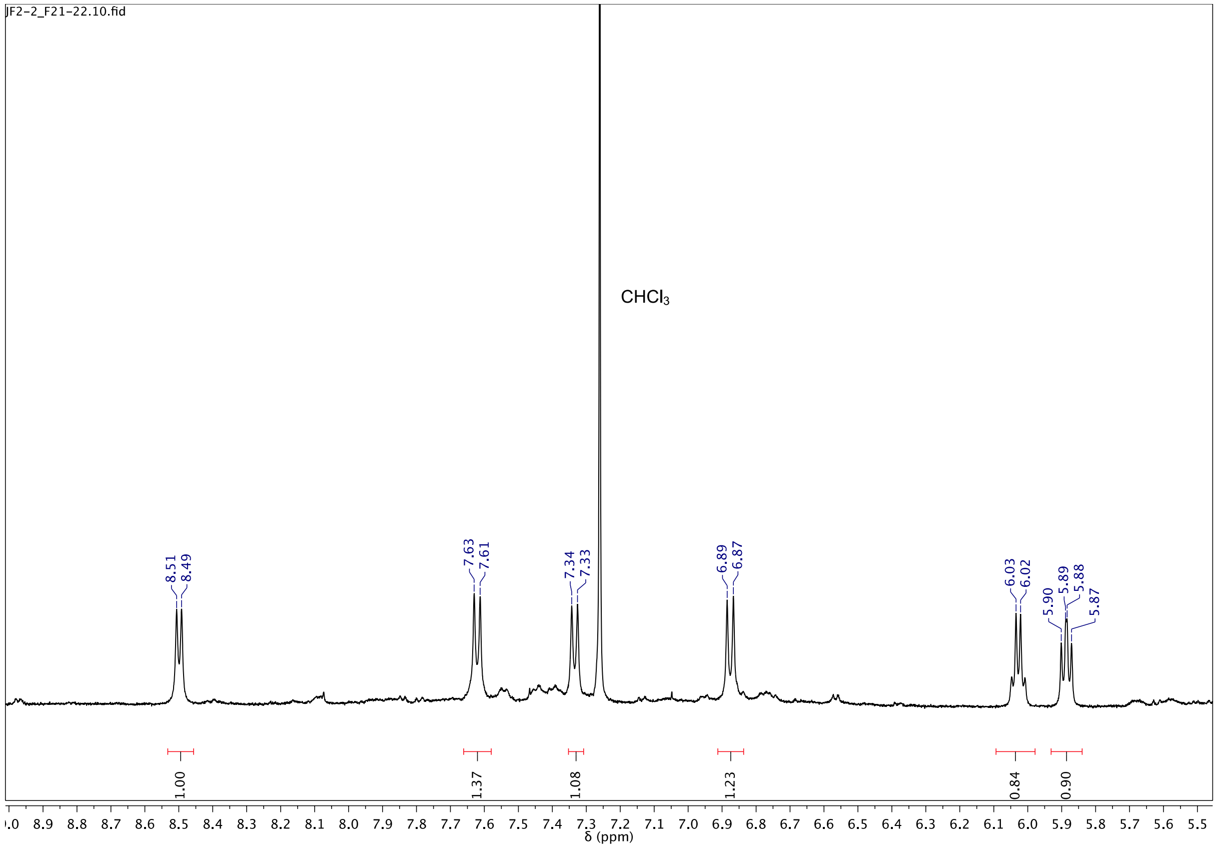

^1H NMR (δ 5.5-2.2)

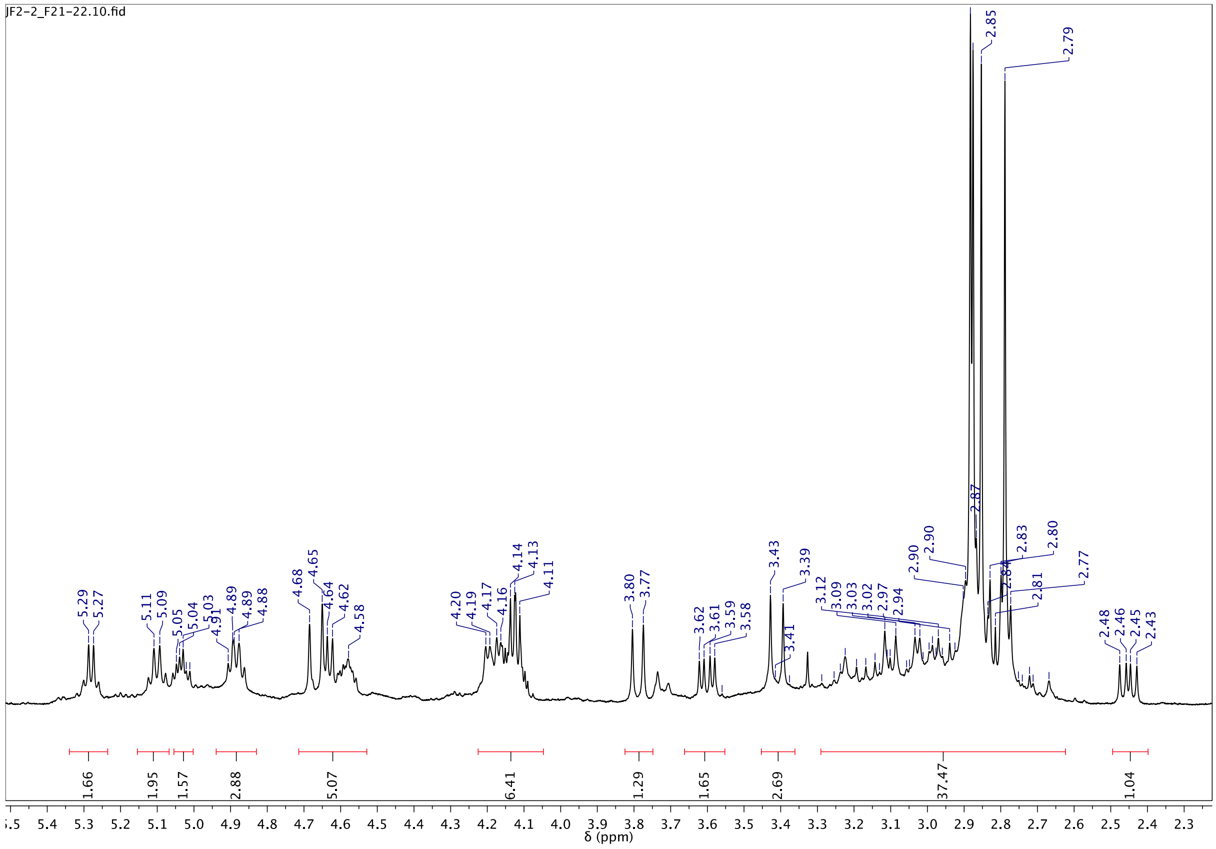

^{1}H NMR (δ 2.2-0.6)

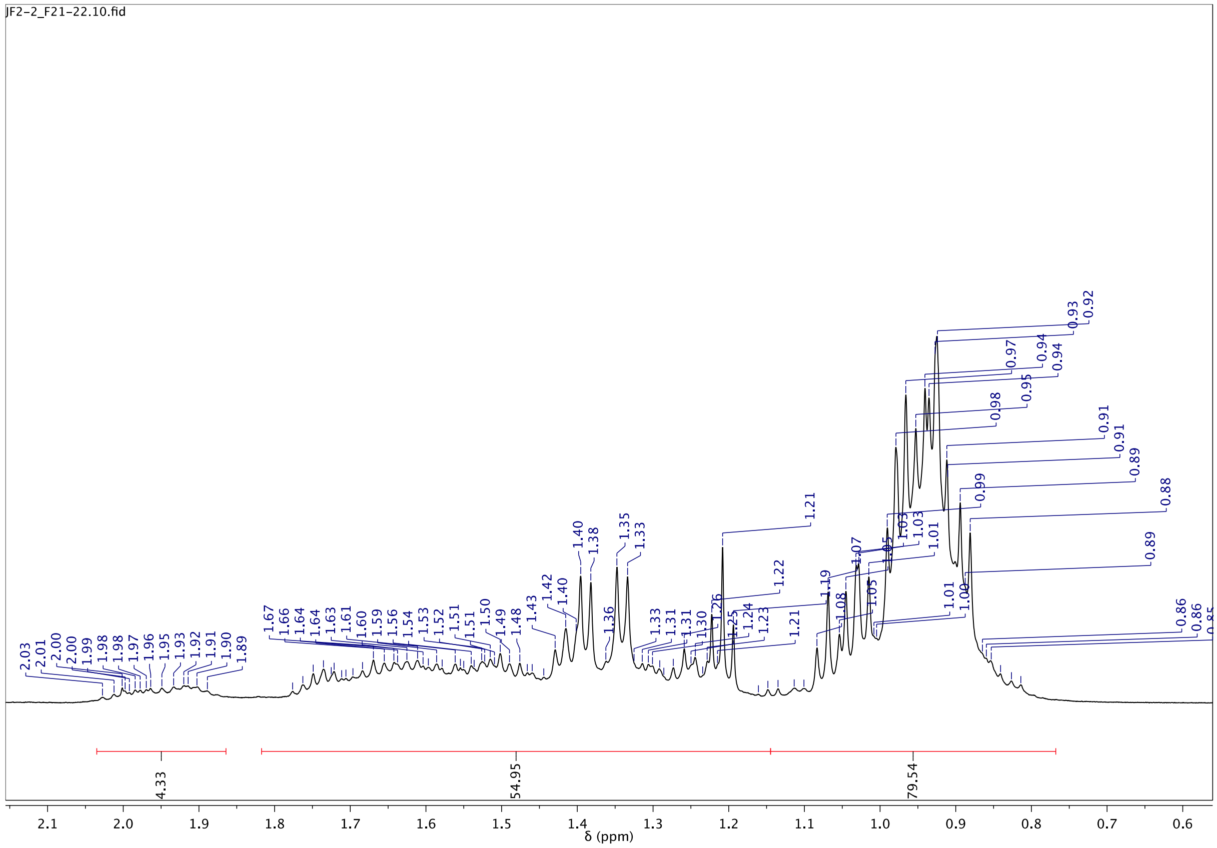

^{13}C NMR

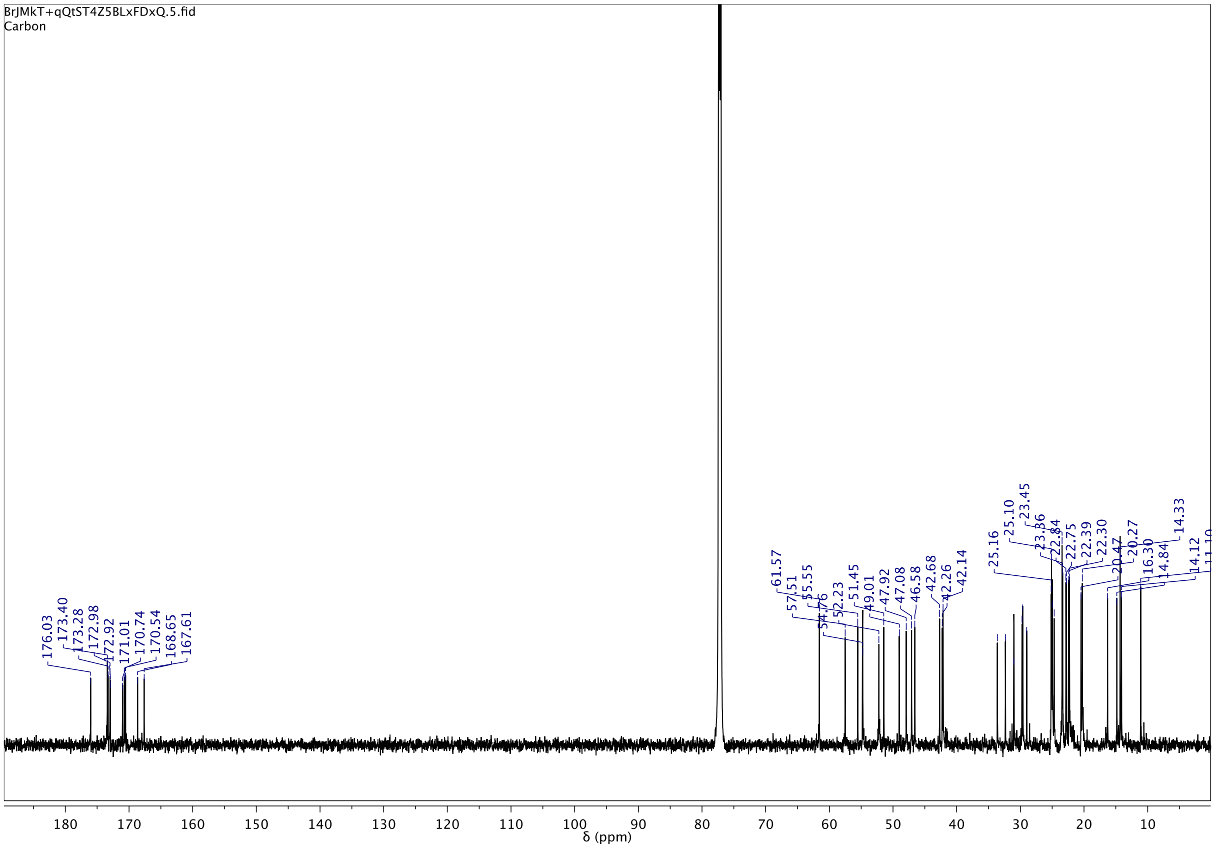

^{13}C NMR (201 MHz, $CDCl_3$) δ 176.03, 173.40, 173.28, 172.98, 172.92, 171.01, 170.74, 170.54, 168.65, 167.61, 61.57, 57.51, 55.55, 54.76, 52.23, 51.45, 49.01, 47.92, 47.08, 46.58, 42.68, 42.26, 42.14, 33.62, 32.35, 31.01, 29.74, 29.62, 29.00, 25.16, 25.10, 24.98, 24.69, 23.45, 23.36, 22.84, 22.75, 22.39, 22.30, 20.47, 20.27, 16.30, 14.84, 14.33, 14.12, 11.10.

^1H-^1H COSY

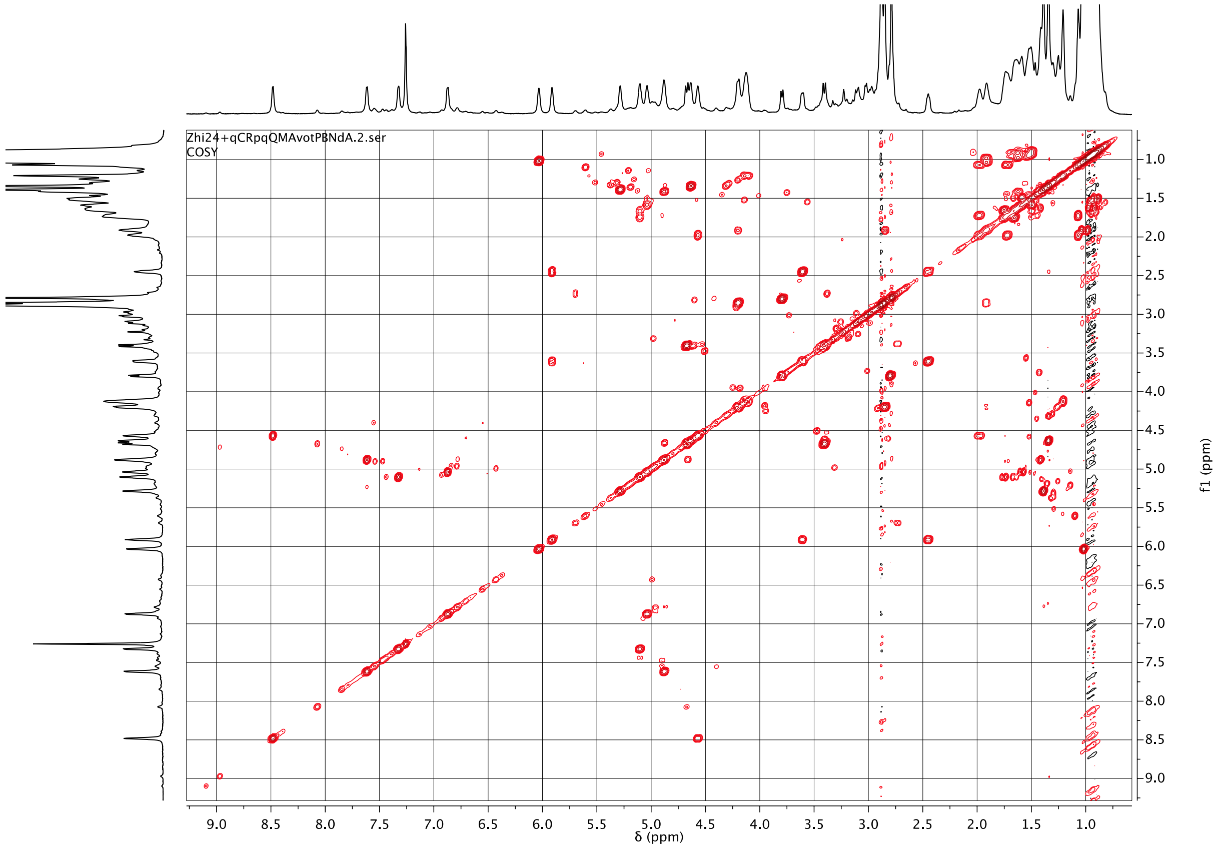

Zhi24+qCRpqQMAvotPBNdA.2.ser
COSY
9.0 8.5 8.0 7.5 7.0 6.5 6.0 5.5 5.0 4.5 4.0 3.5 3.0 2.5 2.0 1.5 1.0
δ (ppm)
f1 (ppm)

^{1}H-^{13}C HSQC

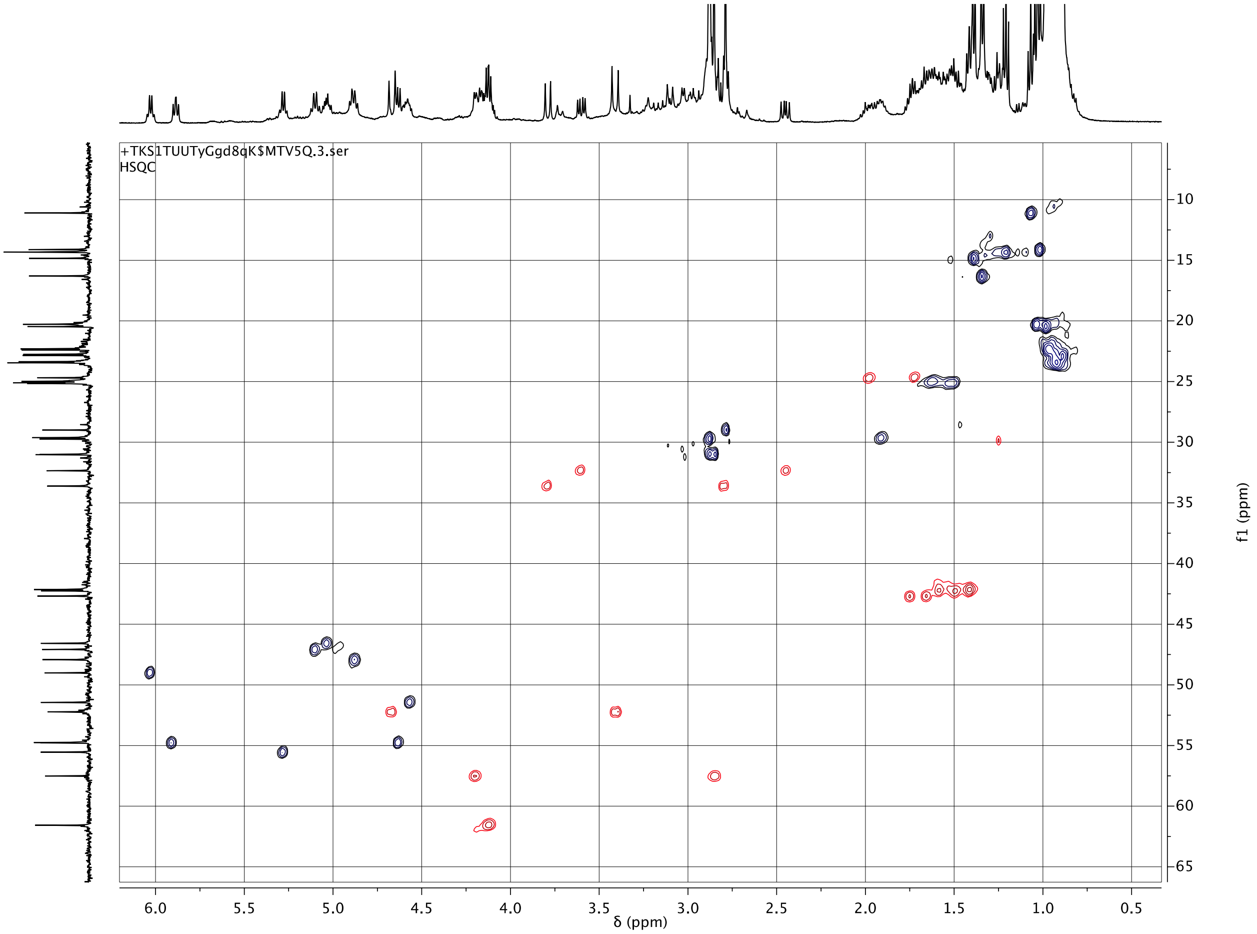

^{1}H-^{13}C HMBC

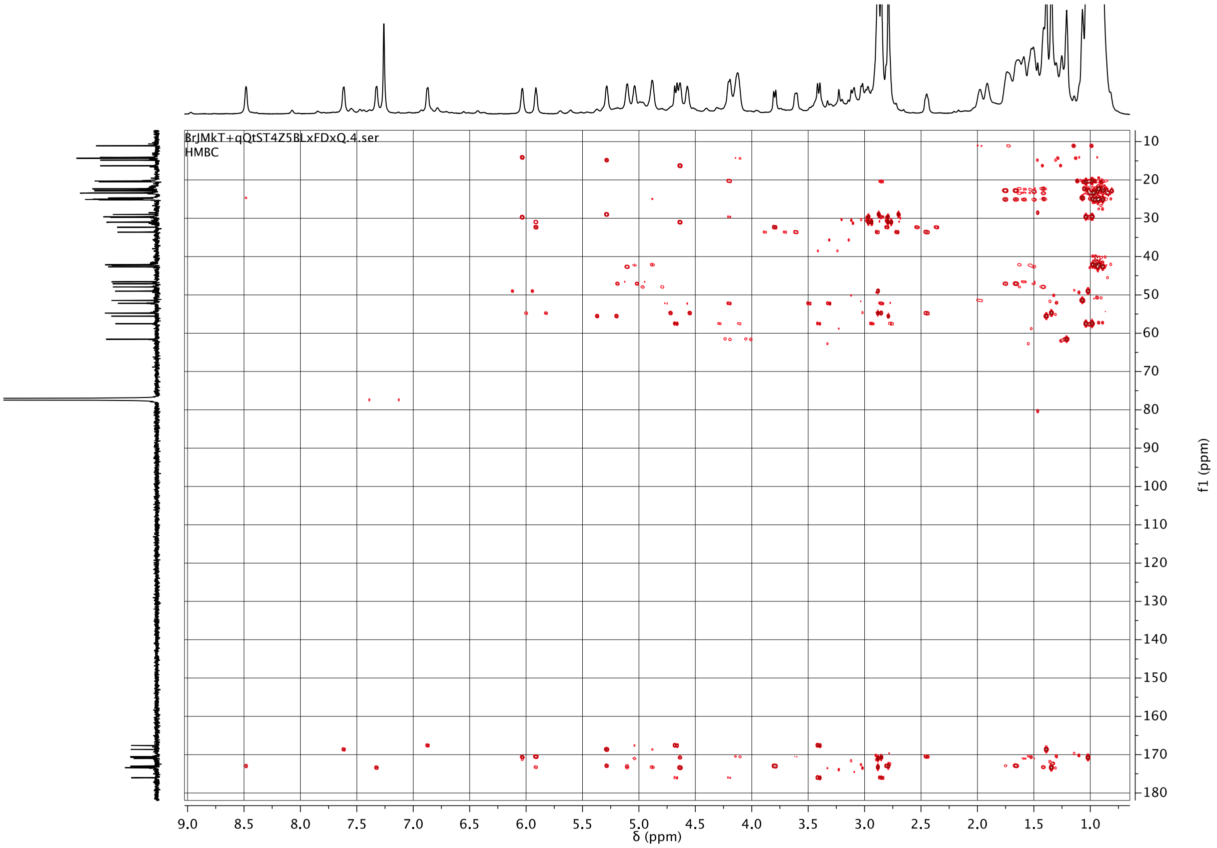
BrJMkT+qQtST4Z5BLxFDxQ.4.ser
HMBC
f1 (ppm)
δ (ppm)
9.0 8.5 8.0 7.5 7.0 6.5 6.0 5.5 5.0 4.5 4.0 3.5 3.0 2.5 2.0 1.5 1.0
10 20 30 40 50 60 70 80 90 100 110 120 130 140 150 160 170 180

^1H-^{13}C HSQC-TOCSY

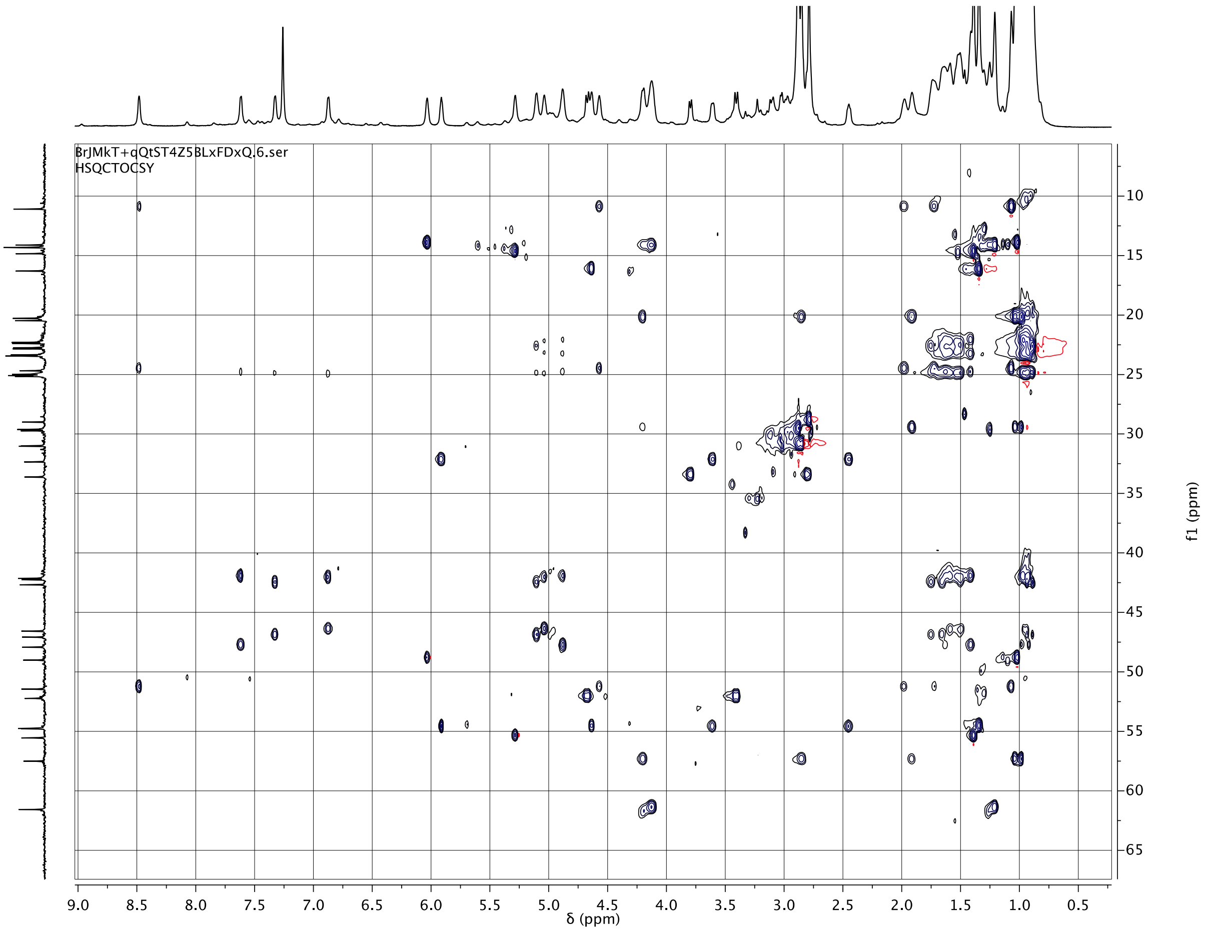

^{1}H-^{1}H TOCSY

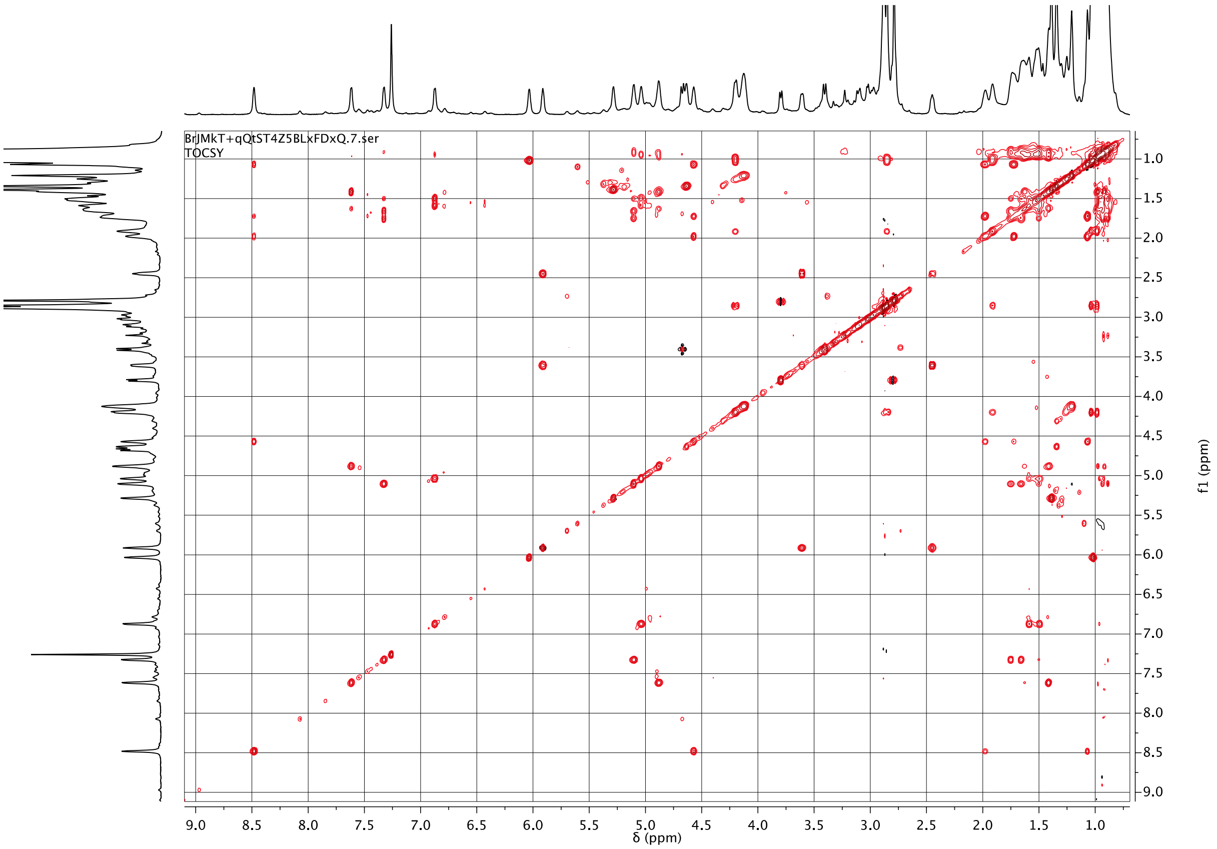

^1H-^1H NOESY (mixing time = 700 ms)

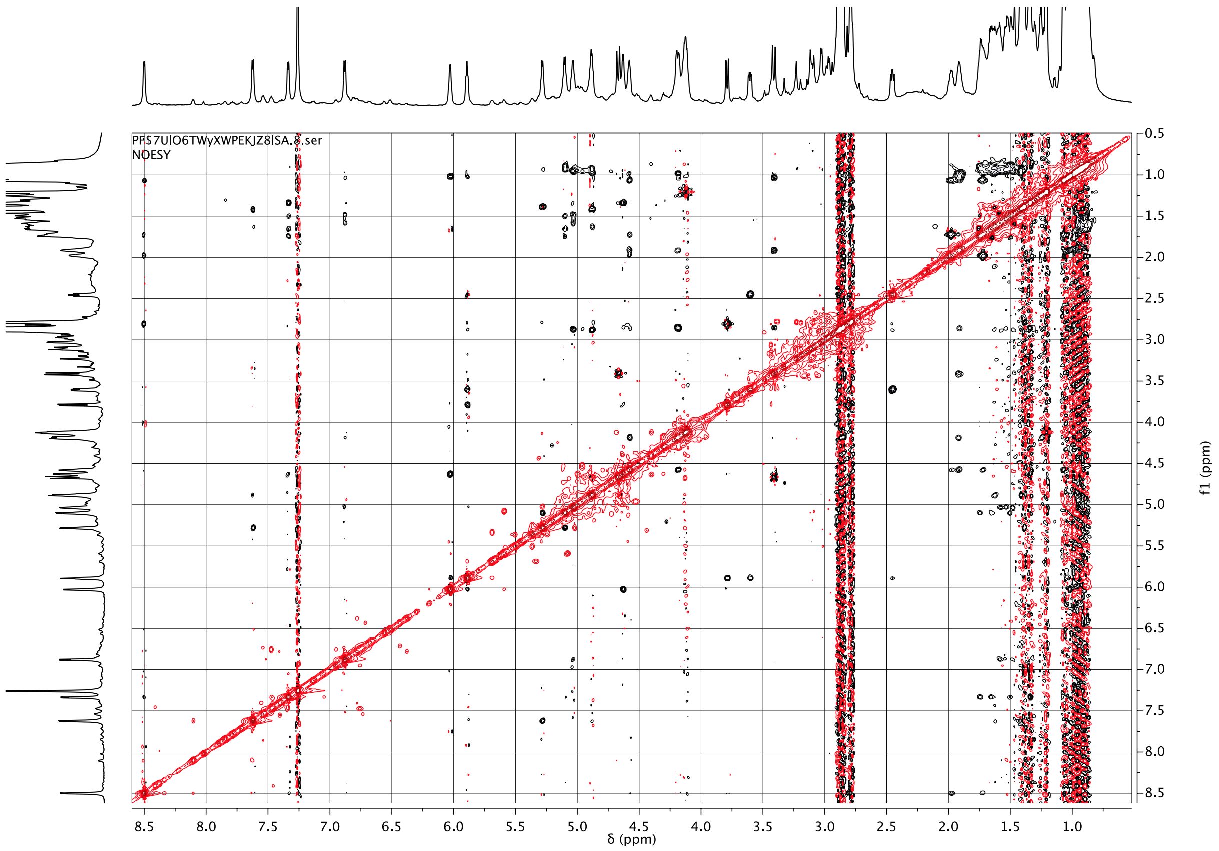

^1H-^1H ROESY

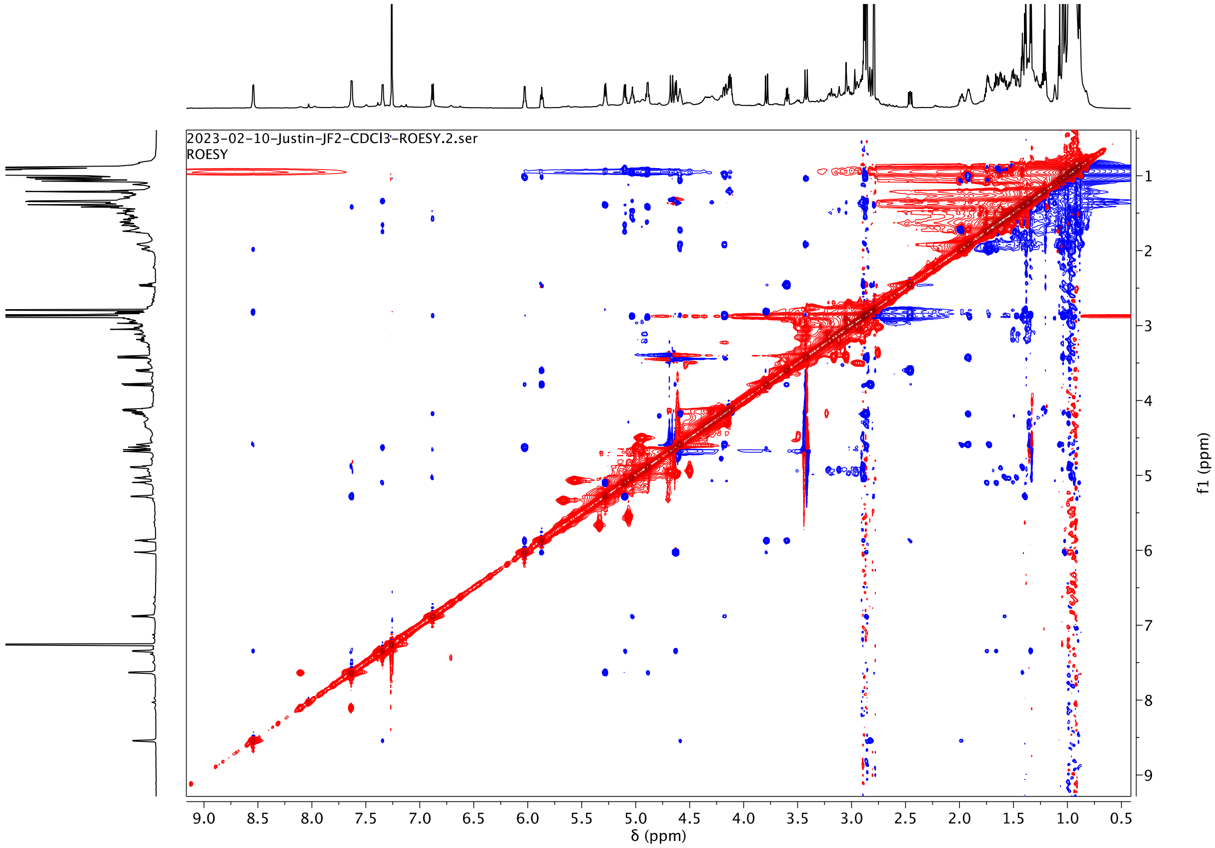
2023-02-10-Justin-JF2-CDCl3-ROESY.2.ser
ROESY
9.0
8.5
8.0
7.5
7.0
6.5
6.0
5.5
5.0
4.5
4.0
3.5
3.0
2.5
2.0
1.5
1.0
0.5
δ (ppm)
1
2
3
4
5
6
7
8
9
f1 (ppm)

^{1}H-^{1}H EASY-ROESY

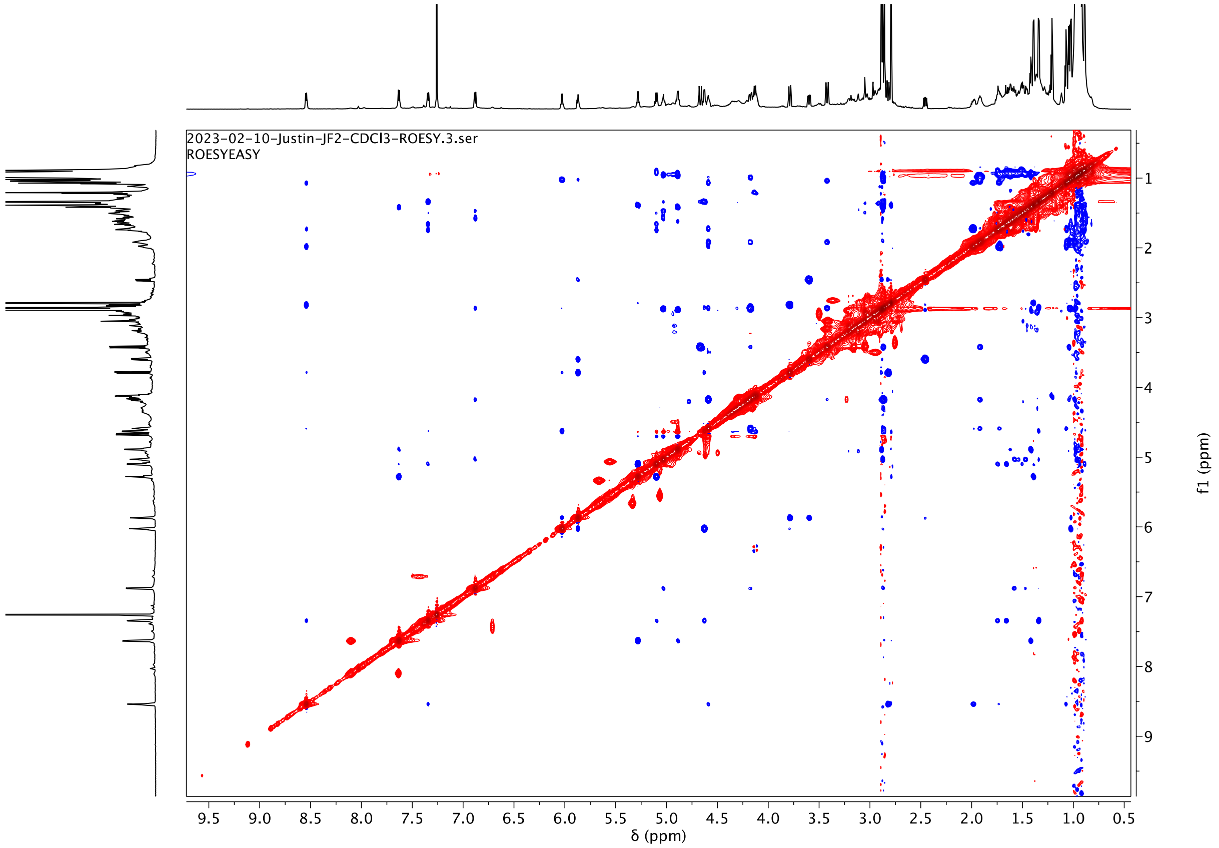

NMR Assignment of **B1**

Residue	Atom group	Splitting (J-coupling)	Proton (^{1}H) δ (ppm)	Carbon (^{13}C) δ (ppm)	Residue	Atom group	Splitting (J-coupling)	Proton (^{1}H) δ (ppm)	Carbon (^{13}C) δ (ppm)
**Acetyl**	methylene	d (14.9)	3.79	33.2	**L-MeAla5**	N-methyl	s	2.85	30.6
	methylene	m	2.79	33.2		α	q (7.1)	4.63	54.4
	Carbonyl-C	-	-	172.6		β	d (6.9)	1.34	15.9
**D-Abu1**	NH	d (7.3)	8.50	-		Carbonyl-C	-	-	173.0
	α	m	4.58	51.0	**D-Leu6**	NH	d (8.5)	7.34	-
	β	m	1.98	24.3		α	dd (15.6, 7.7)	5.10	46.7
	β	m	1.72	24.3		β	m	1.74	42.3
	γ	t (7.4)	1.07	11.1		β	m	1.66	42.3
	Carbonyl-C	-	-	175.6		γ	m	1.49	24.8
**iBu-Gly**	α (backbone)	d (17.3)	4.67	51.8		δ	m	0.94	22.8
	α (backbone)	d (17.3)	3.41	51.8		δ	m	0.89	22.8
	β (iBu)	m	4.19	57.1		Carbonyl-C	-	-	172.5
	β (iBu)	m	2.86	57.1	**D-MeAla7**	N-methyl	s	2.79	28.6
	γ (iBu)	m	1.92	29.2		α	q (6.8)	5.28	55.2
	δ (iBu)	m	0.99	20.5		β	d (6.7)	1.39	14.5
	δ (iBu)	m	1.04	20.3		Carbonyl-C	-	-	168.3
	Carbonyl-C	-	-	167.2	**L-Leu8**	NH	d (8.8)	7.62	-
**L-Leu3**	NH	d (9.3)	6.88	-		α	dd (15.4, 6.7)	4.89	47.6
	α	td (9.1, 4.8)	5.04	46.2		β	m	1.58	41.7
	β	m	1.58	41.9		β	m	1.41	41.7
	β	m	1.48	41.9		γ	m	1.63	24.6
	γ	m	1.53	24.8		δ	m	0.98	22.3
	δ	m	0.97	22.4		δ	m	0.97	23.4
	δ	m	0.94	23.4		Carbonyl-C	-	-	172.9
	Carbonyl-C	-	-	170.6	**L-MeCys9**	N-methyl	s	2.88	30.6
**D-MeAla4**	N-methyl	s	2.88	29.4		α	dd (8.7, 6.4)	5.89	54.4
	α	q (6.3)	6.03	48.6		β	dd (14.6, 6.5)	3.60	32.0
	β	m	1.02	13.7		β	dd (14.6, 8.7)	2.45	32.0
	Carbonyl-C	-	-	170.4		Carbonyl-C	-	-	170.2
					**Ethyl ester**	methylene	m	4.13	61.2
						methyl	t (7.5)	1.21	13.9

Amide NH Temperature Shifts ($CDCl_3$)

	Abu1	Leu8	Leu6	Leu3
Δδ/ΔT (ppb/K)	-2.48	-0.96	-1.26	-0.87

Temperature shift data was acquired in $CDCl_3$ and calculated from the chemical shift at the following temperature points in K: 300, 305, 310, 315, 320, and 323. The prior to acquisition of the spectra at each temperature a 10-minute equilibration period was observed.

Solution Structure Calculation using CYANA

Solution structure calculation of macrocycle **B1** was performed using the CYANA structure calculation algorithm (Combined Assignment and Dynamics Algorithm for NMR Applications) from NMR-derived distance restraints and J-couplings.[15] A set of 20 lowest energy structures were generated by CYANA 3.98.15 from 1000 structures based on NOE distances (**Table S3**) extracted from ROESY-NMR Spectroscopy and $J_{H\text{-}HA}$ couplings using standard simulated annealing CYANA protocol with 50,000 torsion angle dynamics steps per conformations.

The topology files of non-natural amino acids for CYANA calculations were generated using Spartan 20 (Spartan 20, Wavefunction, Inc) using ab-initio calculations with the Hartree-Fock 6-31G* basis set, and then 3D coordinates of each non-natural amino acids were converted into CYANA residue library format by using CYLIB 2.0[16] algorithm.

The CYANA calculated structure was further refined by energy minimization in MOE (Molecular Operating Environment (MOE), 2020.09; Chemical Computing Group ULC: 1010 Sherbooke St. West, Suite #910, Montreal, QC, Canada H3A 2R7, 2022.) by CHARMM27 force field with a Born implicit electrostatic model using dielectric constant of NMR solvents, ε=4.8 for Chloroform and ε=2.0 for cyclohexane) with maximum deviation from CYANA structure set to RMSD < 0.5 Å.

McMD data

Molecular Shape Analysis

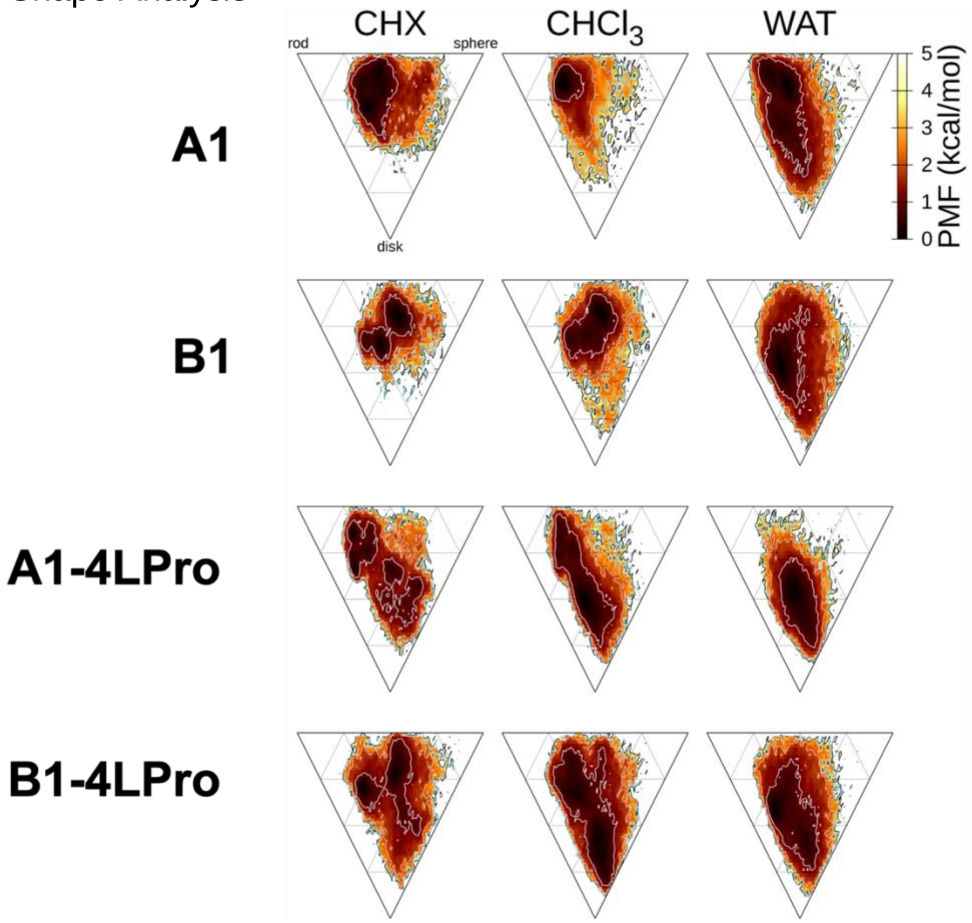

Principal Component Analysis

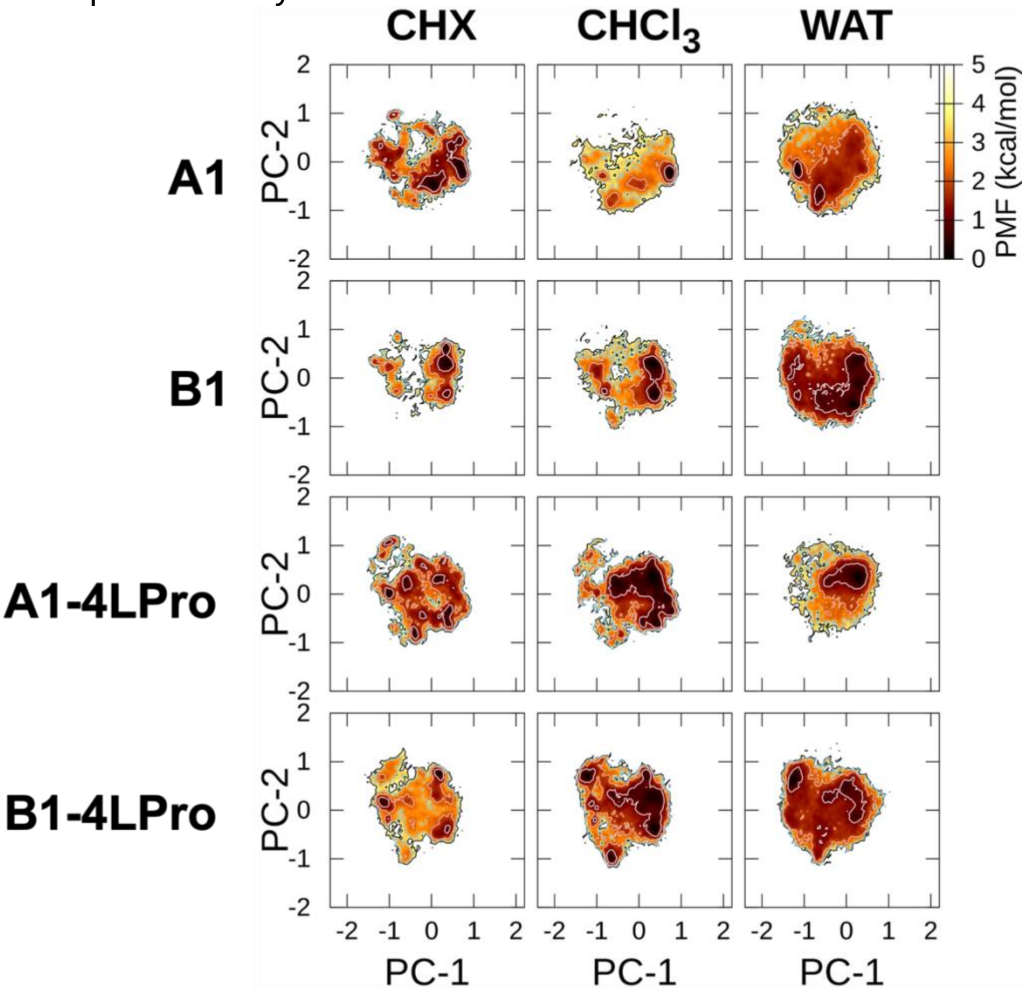

Hydrogen-bond Plots

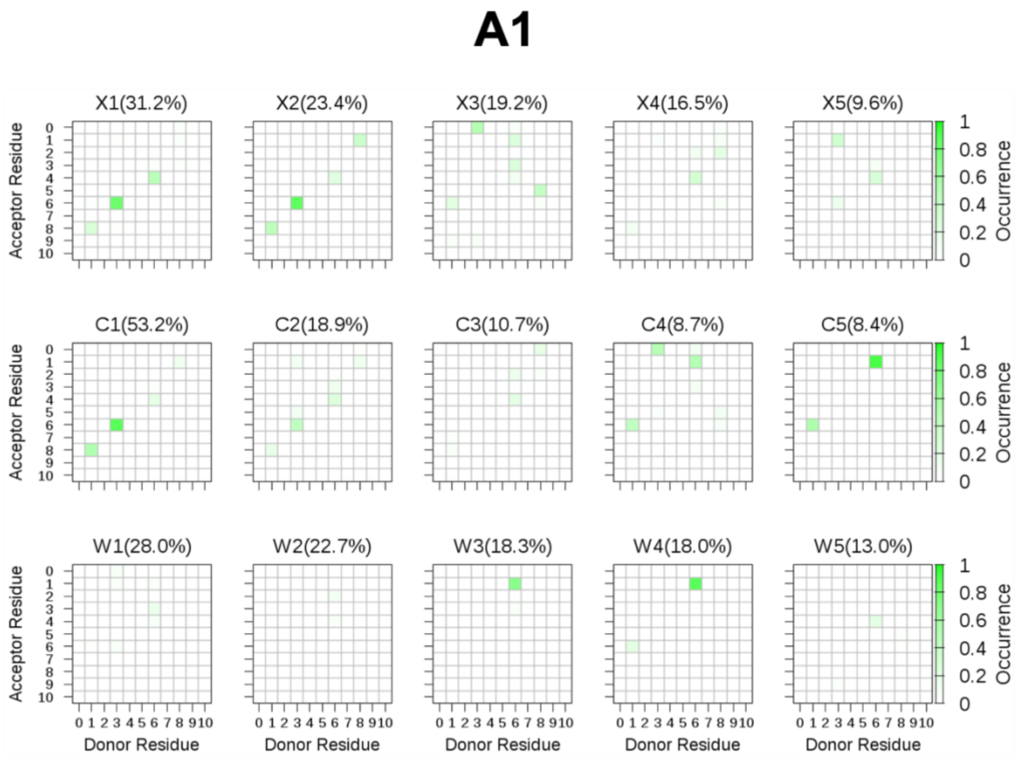

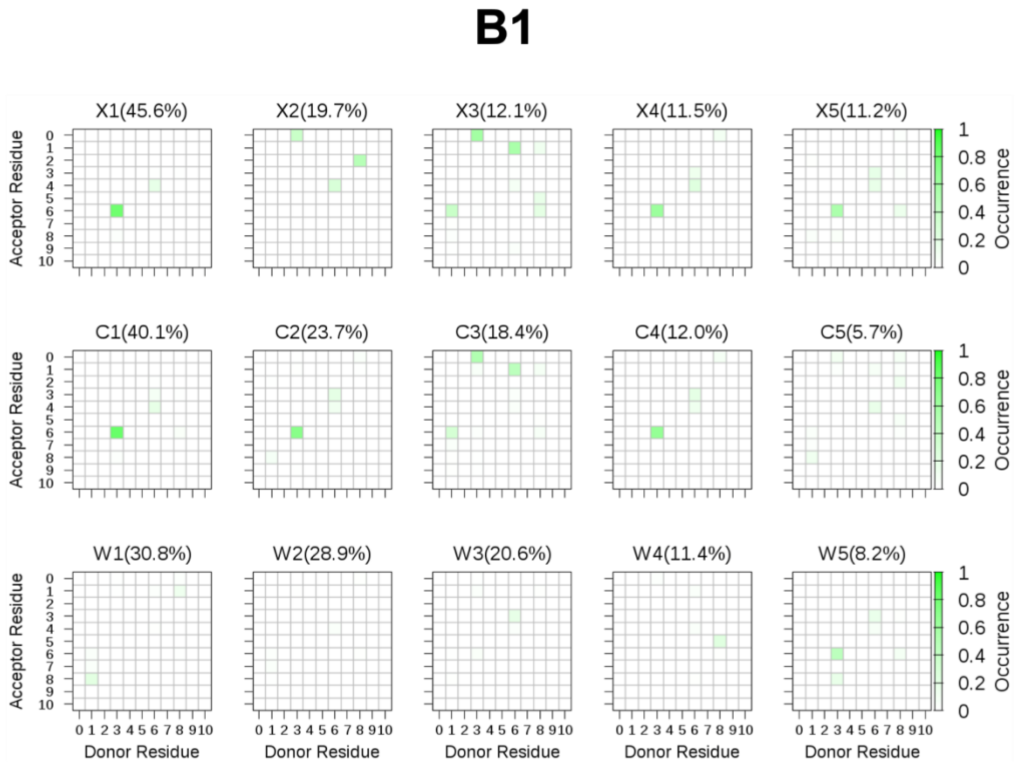

A1-4LPro

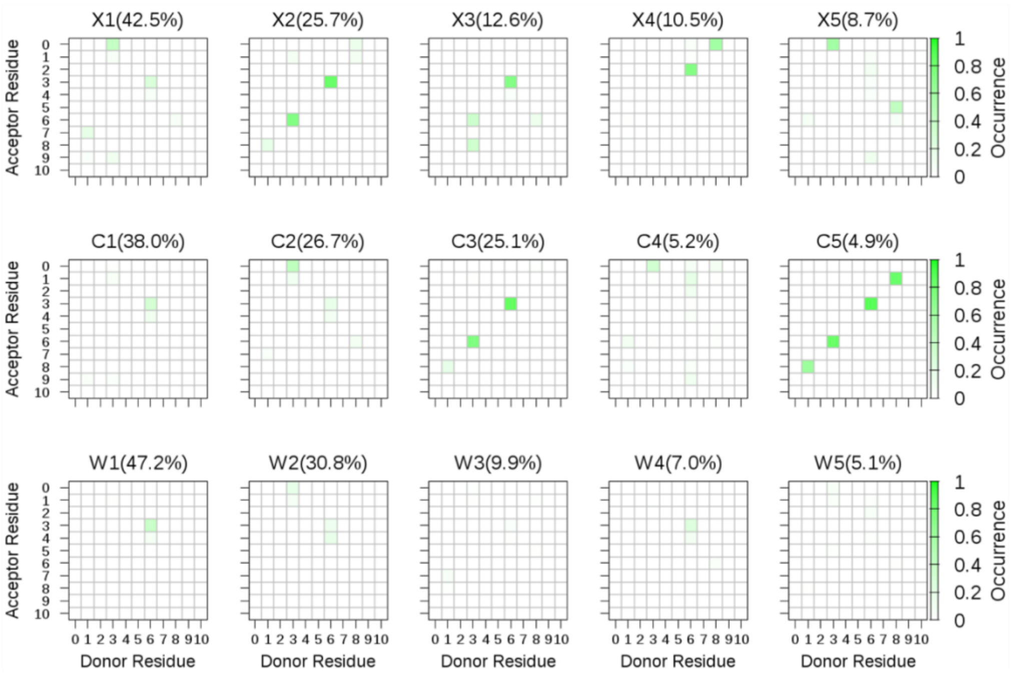

B1-4LPro

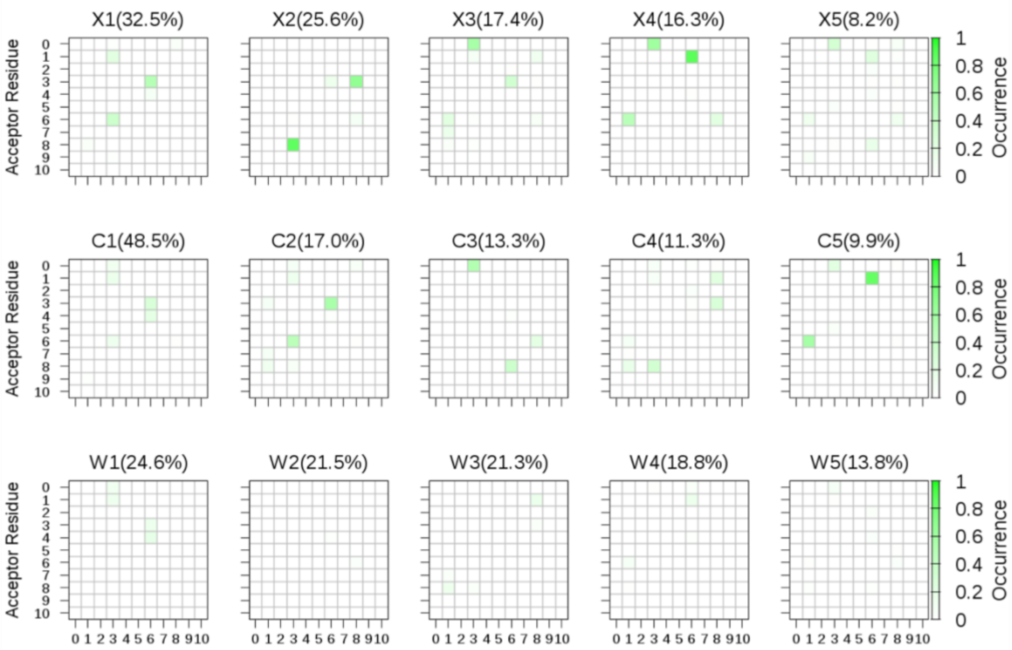

Additional CD spectra

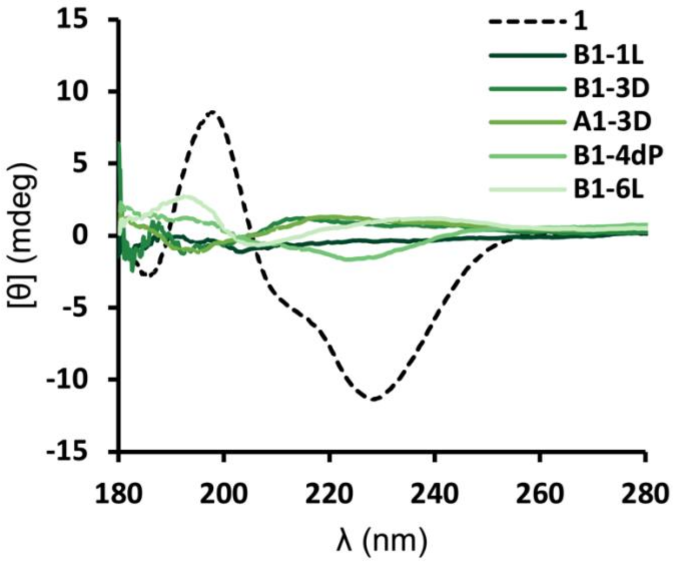

50 μM in cyclohexane

LCMS traces and NMR spectra of pure compounds

Decapeptide **1**

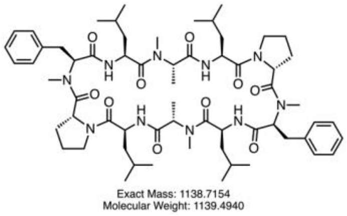

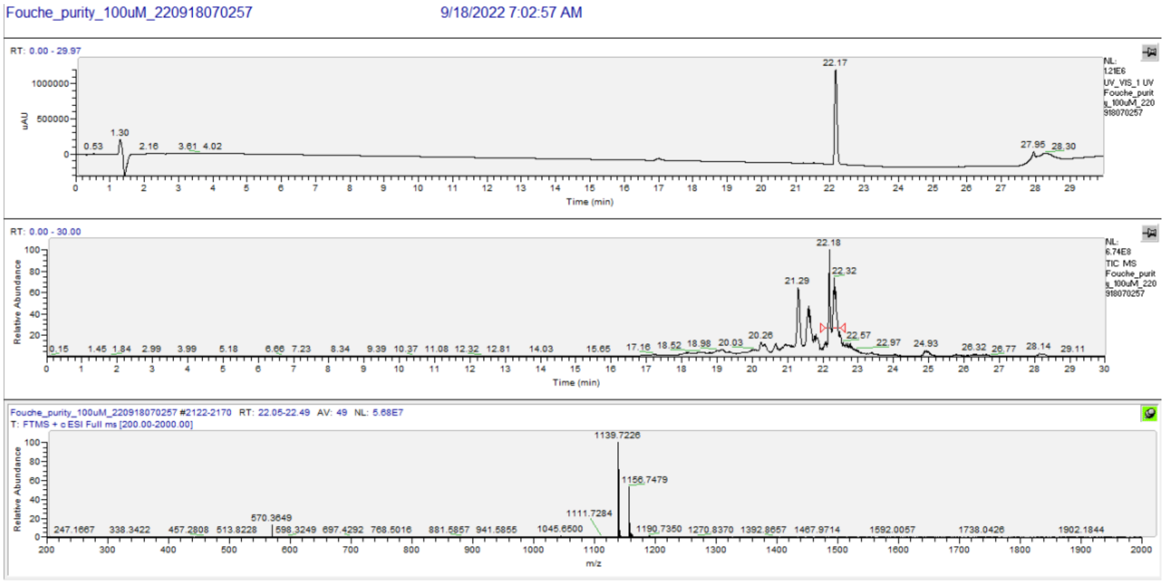

^{1}H NMR ($CDCl_3$)

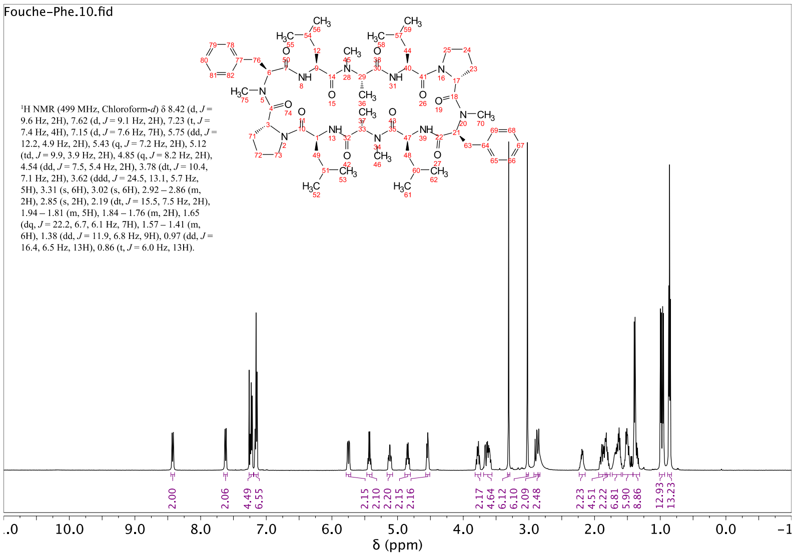

Liposcan

LA02

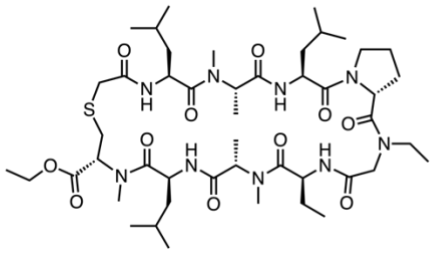

Exact Mass: 979.5776
Molecular Weight: 980.2770

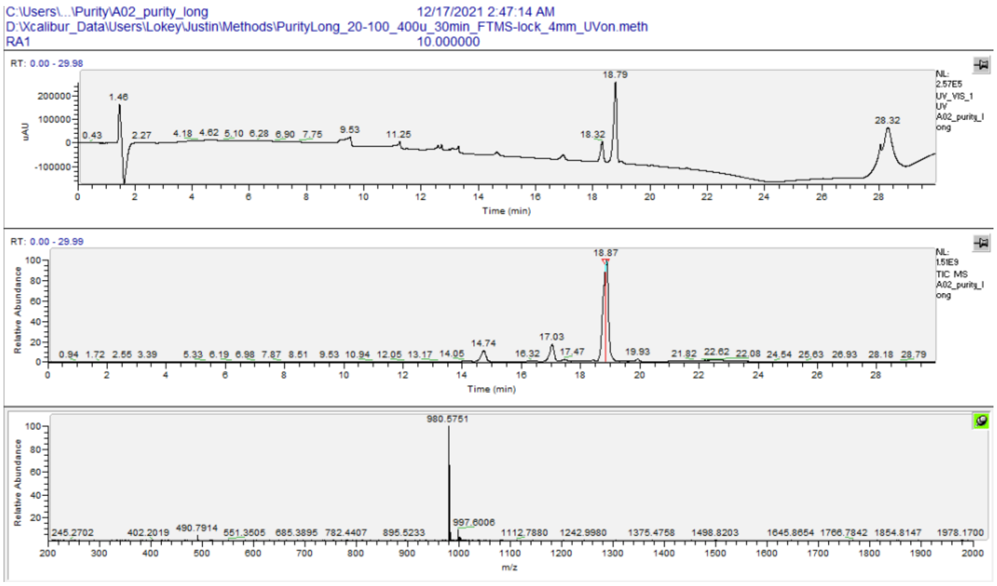

^{1}H NMR ($CDCl_3$)

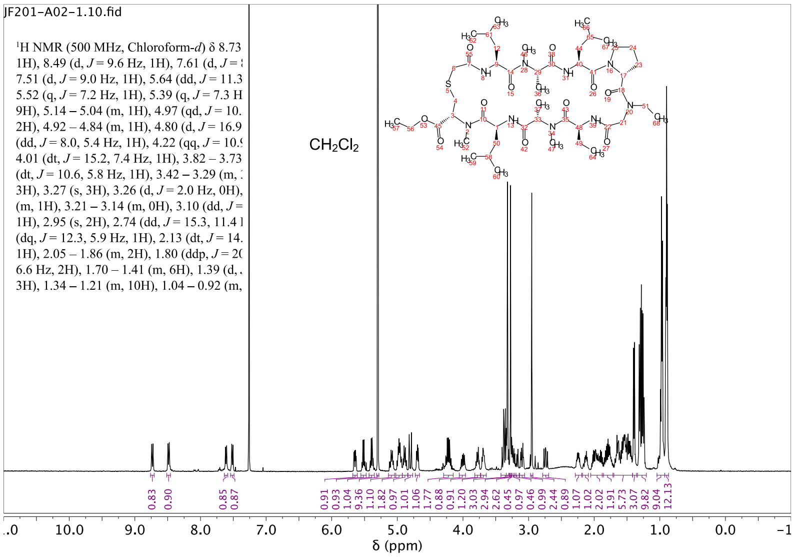

LA04

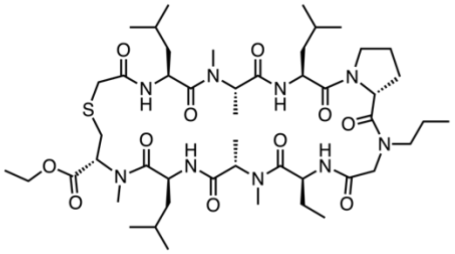

Exact Mass: 993.5933
Molecular Weight: 994.3040

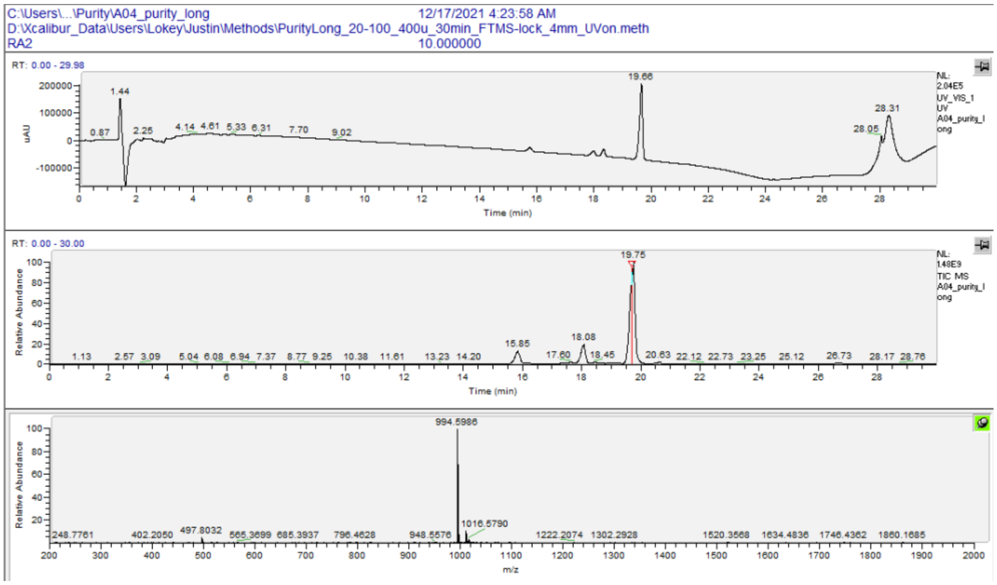

^{1}H NMR ($CDCl_3$)

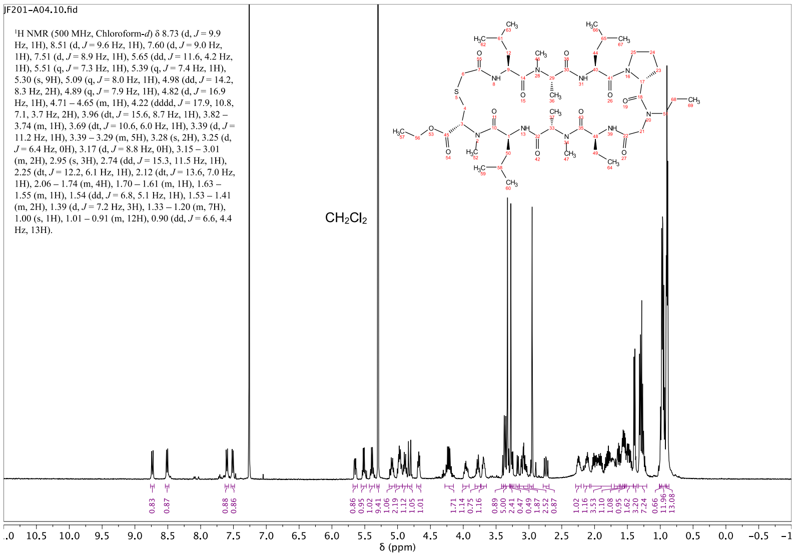

LA07

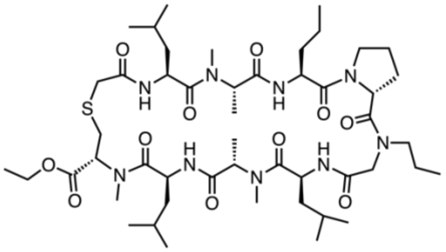

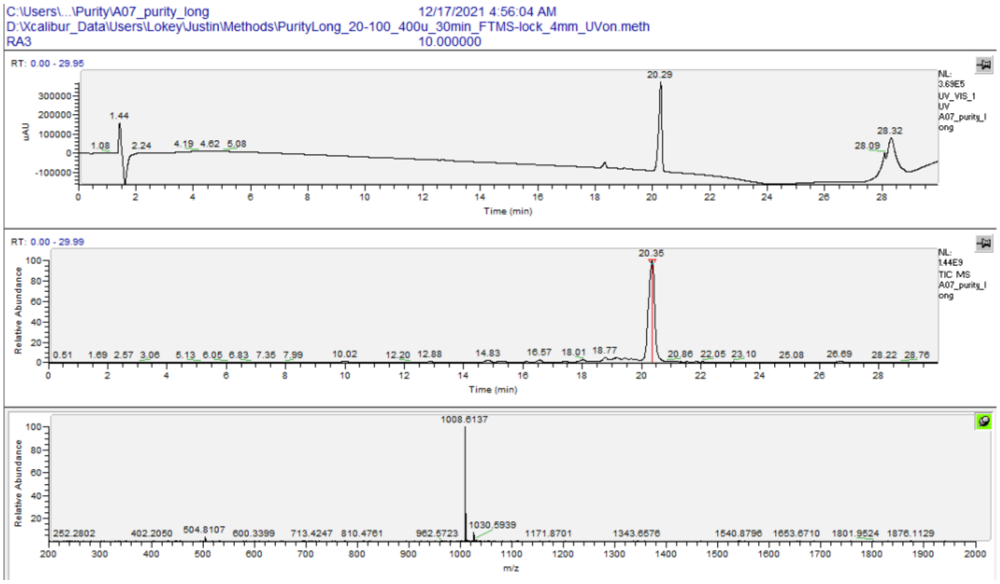

^{1}H NMR ($CDCl_3$)

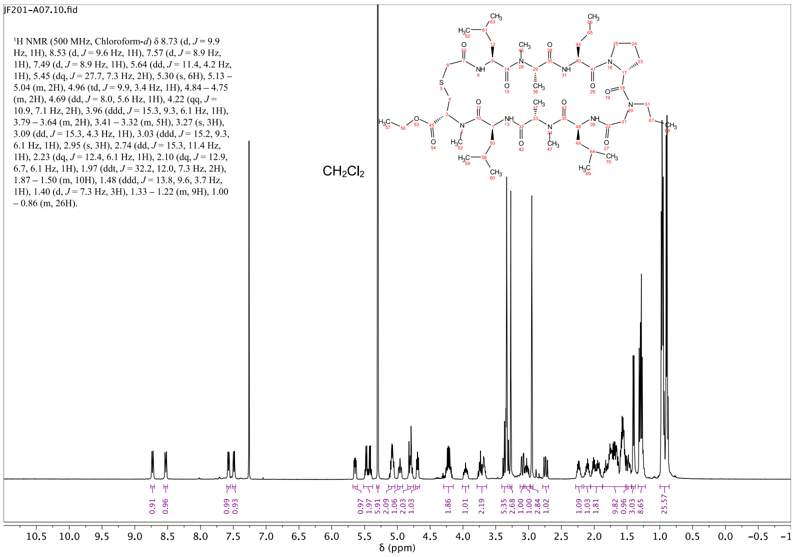

LA09

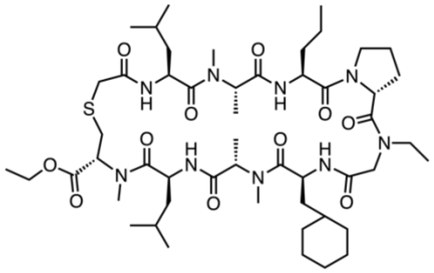

Exact Mass: 1033.6246
Molecular Weight: 1034.3690

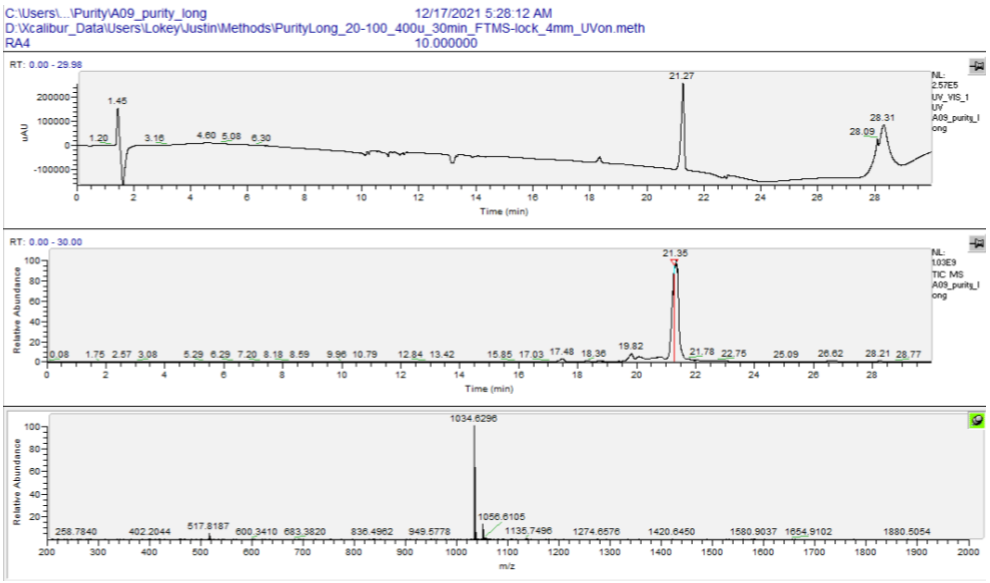

^{1}H NMR ($CDCl_3$)

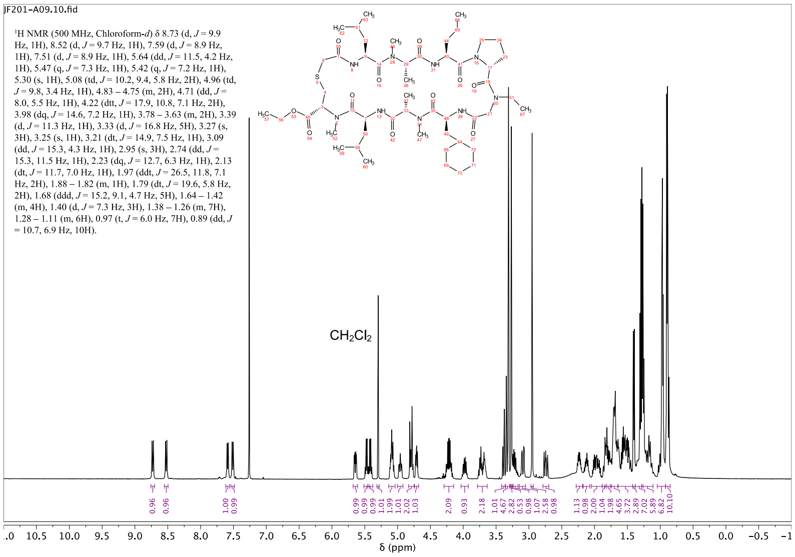

LB02

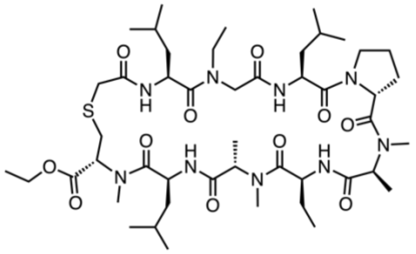

Exact Mass: 979.5776
Molecular Weight: 980.2770

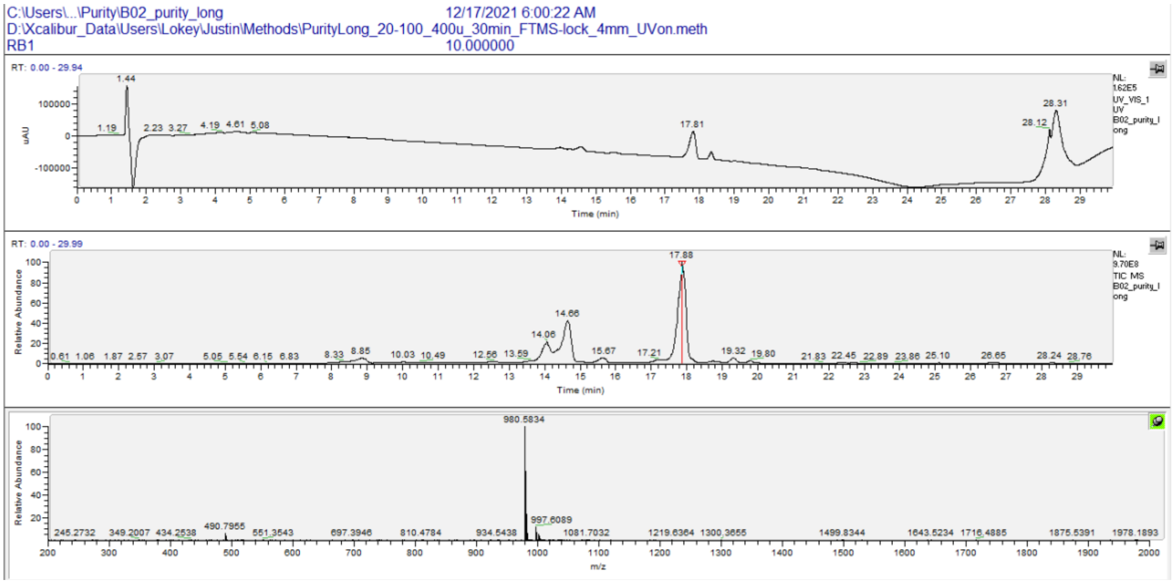

^{1}H NMR ($CDCl_3$)

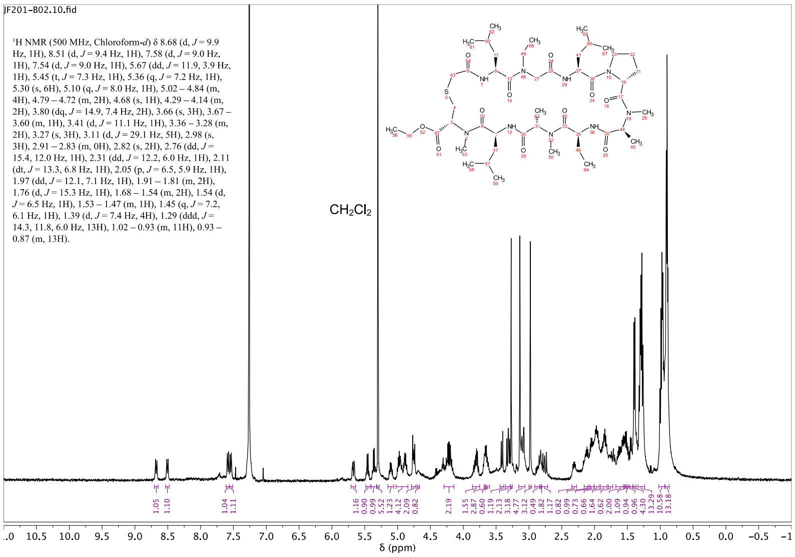

LB04

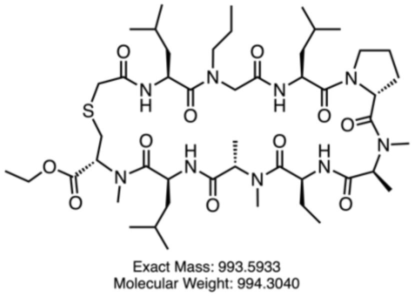

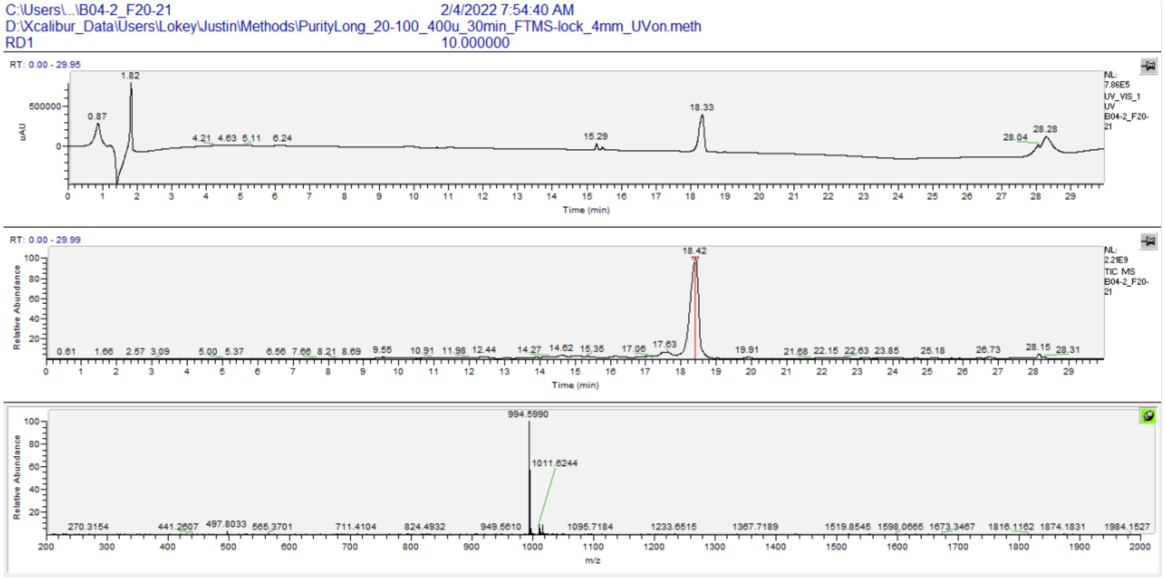

^{1}H NMR ($CDCl_3$)

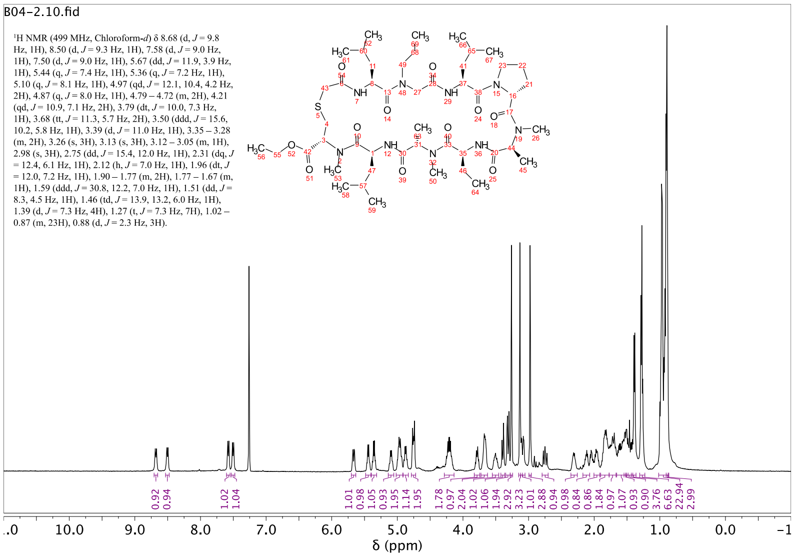

LB07

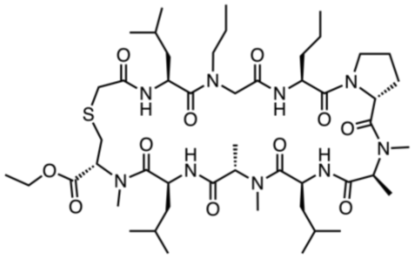

Exact Mass: 1007.6089
Molecular Weight: 1008.3310

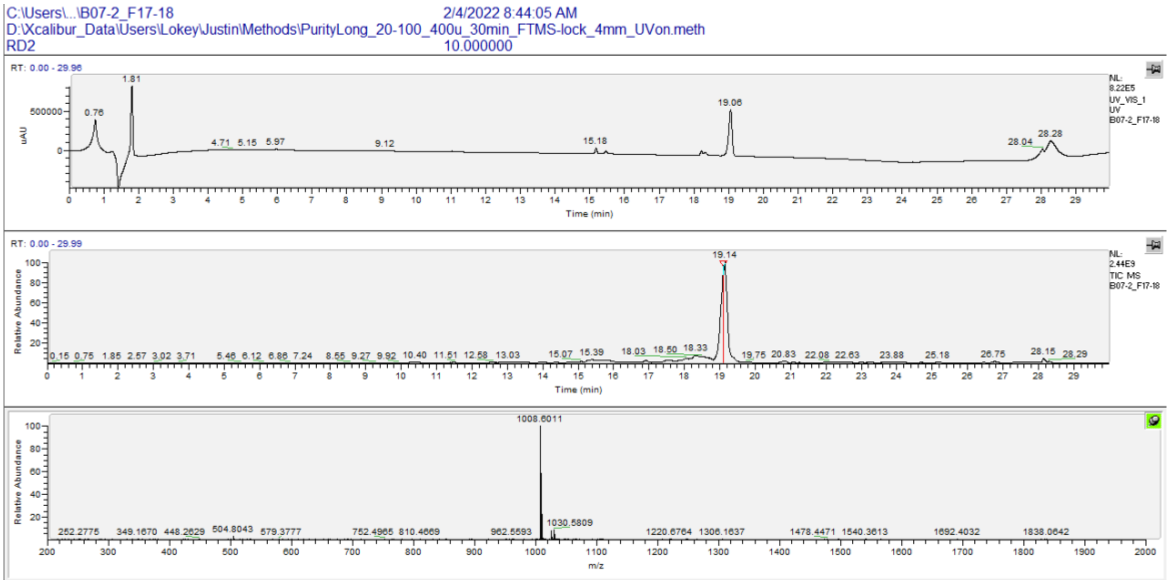

^{1}H NMR ($CDCl_3$)

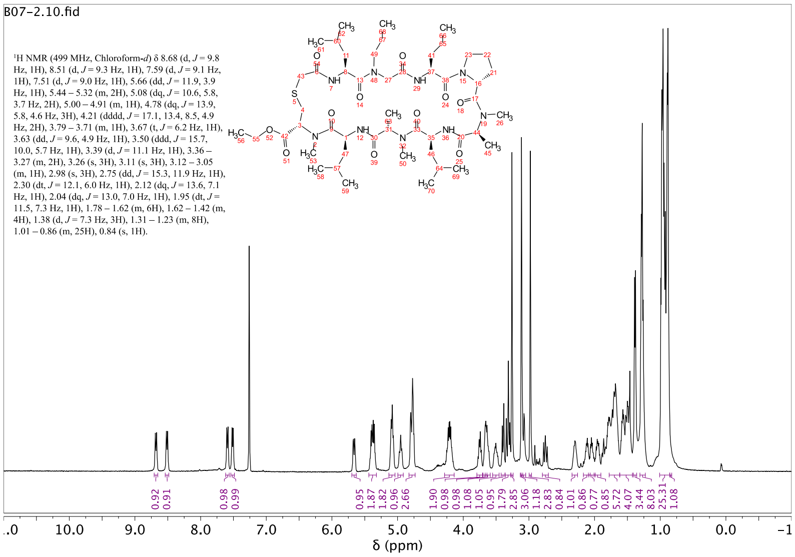

LB09

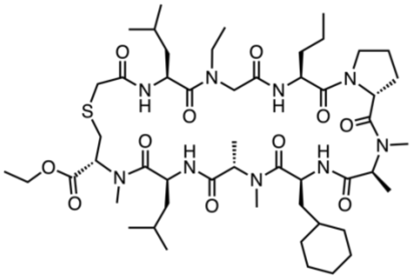

Exact Mass: 1033.6246
Molecular Weight: 1034.3690

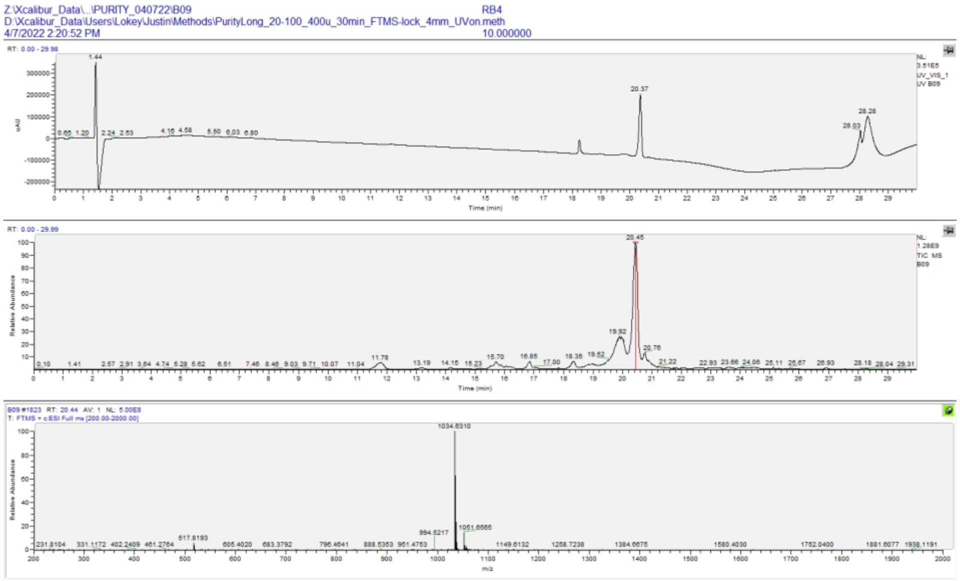

^1H NMR ($CDCl_3$)

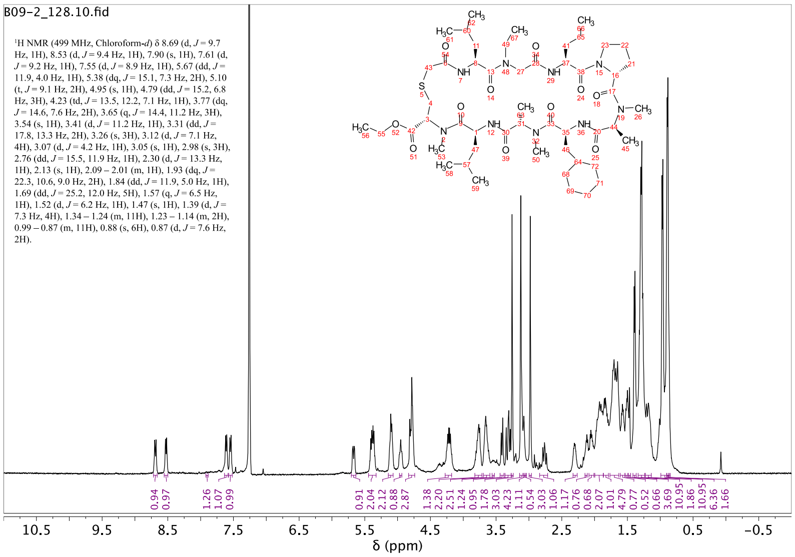

LC02

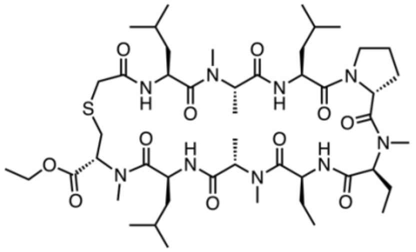

Exact Mass: 993.5933
Molecular Weight: 994.3040

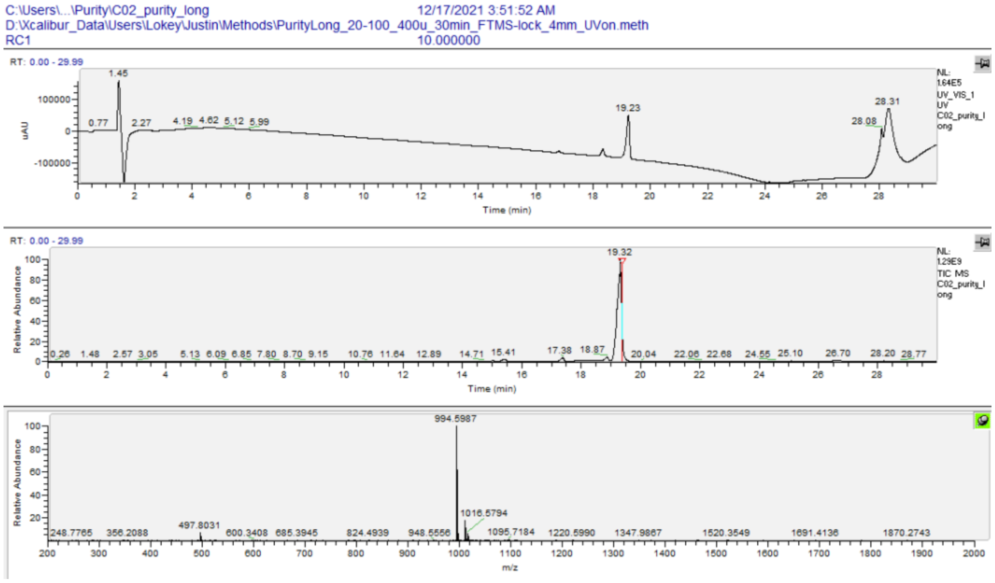

^{1}H NMR ($CDCl_3$)

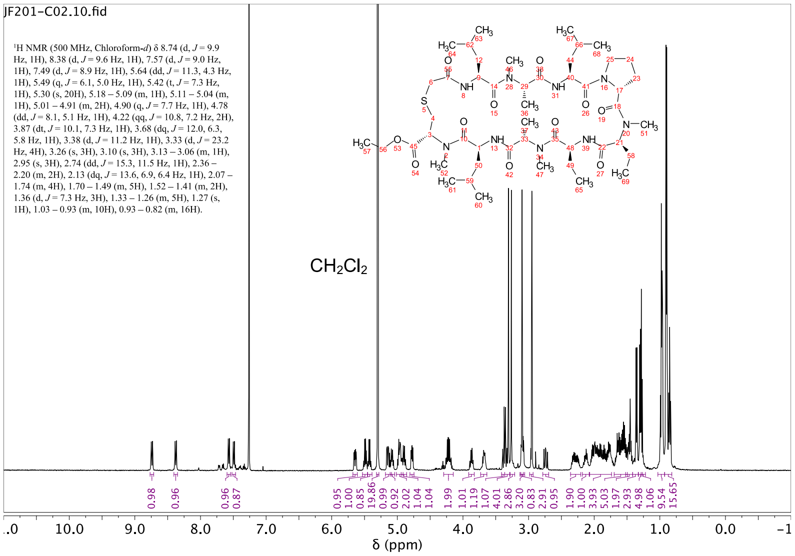

LC04

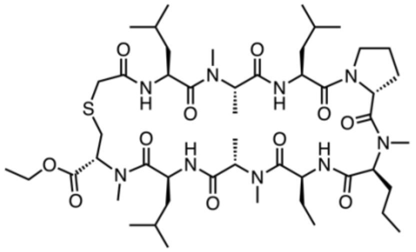

Exact Mass: 1007.6089
Molecular Weight: 1008.3310

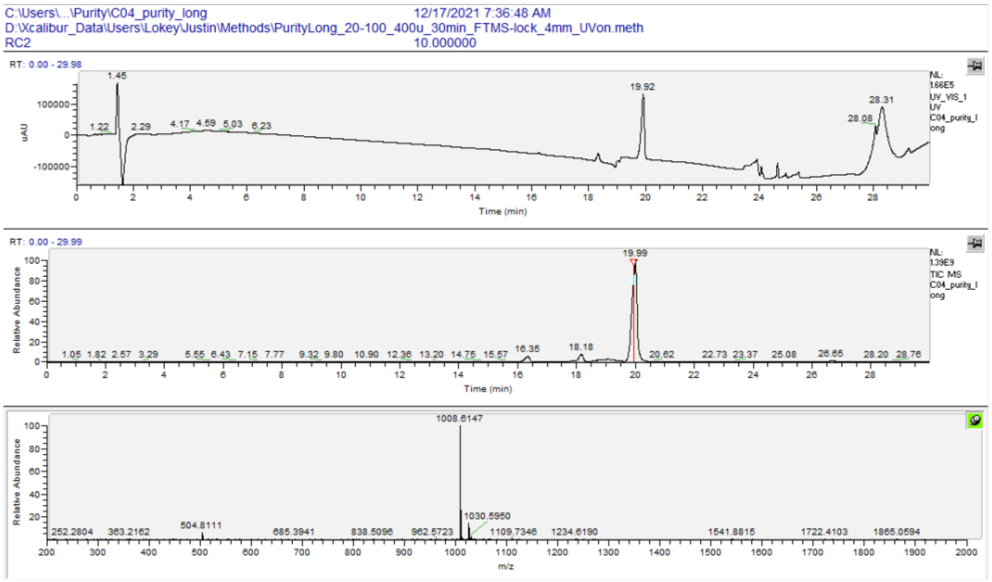

^{1}H NMR ($CDCl_3$)

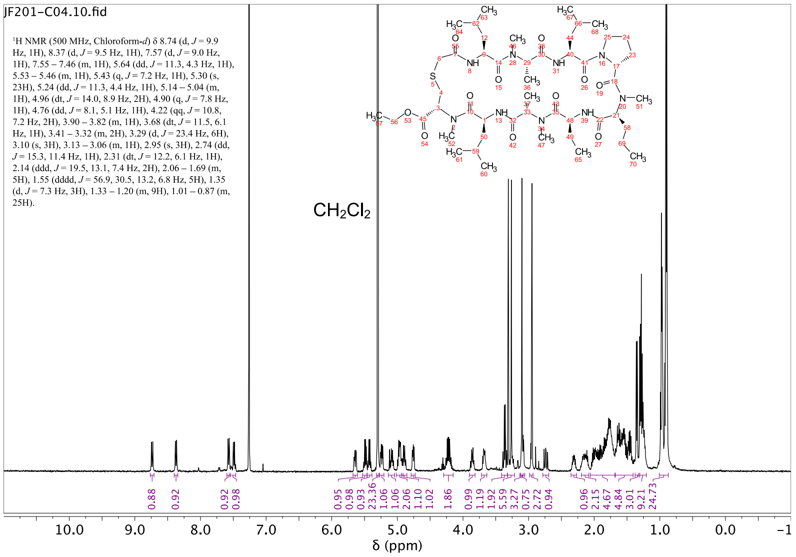

LC07

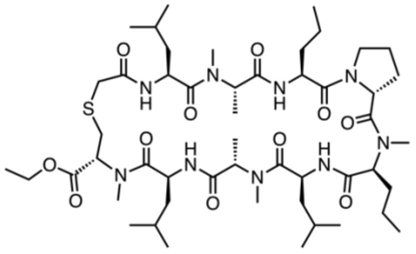

Exact Mass: 1021.6246
Molecular Weight: 1022.3580

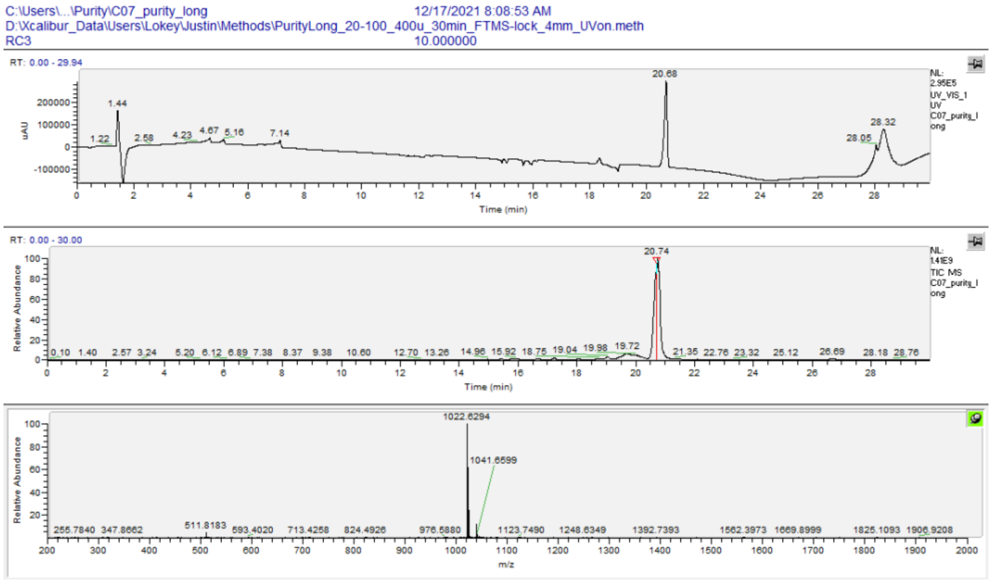

^{1}H NMR ($CDCl_3$)

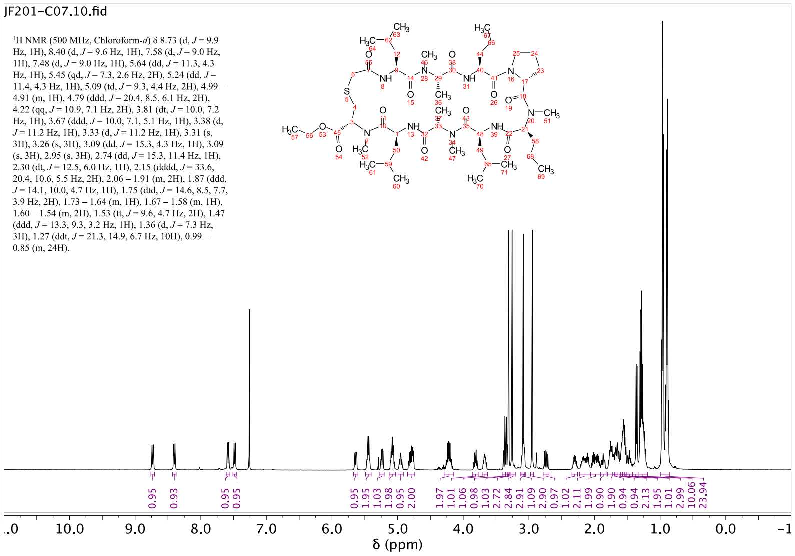

LC09

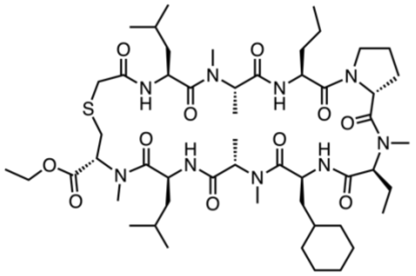

Exact Mass: 1047.6402
Molecular Weight: 1048.3960

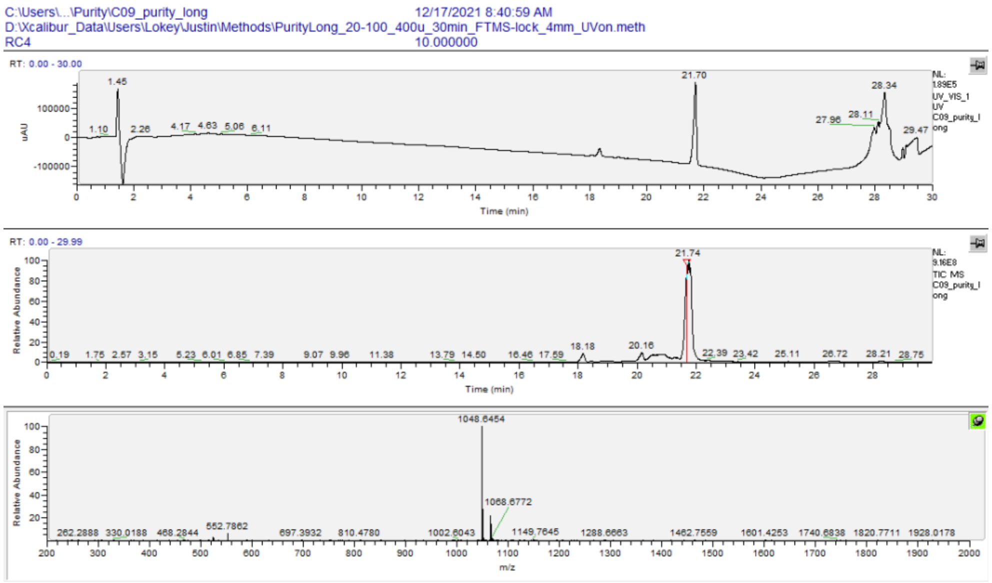

^{1}H NMR ($CDCl_3$)

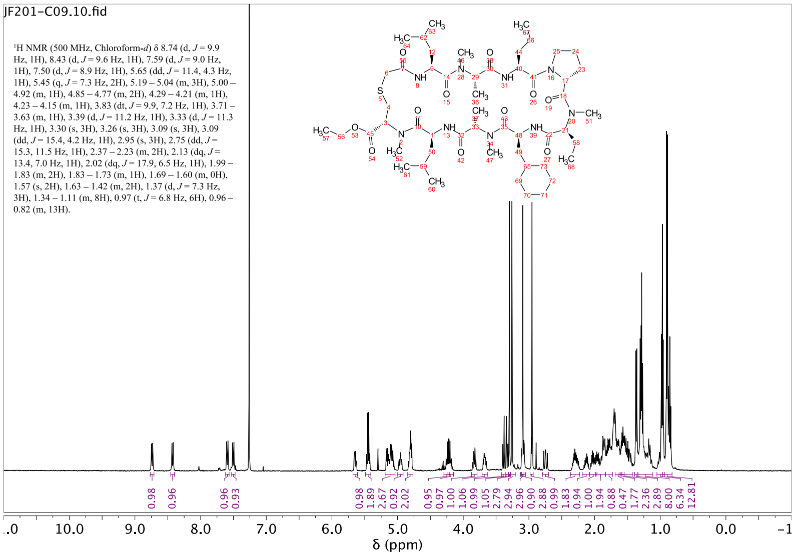

Stereoscan

A1

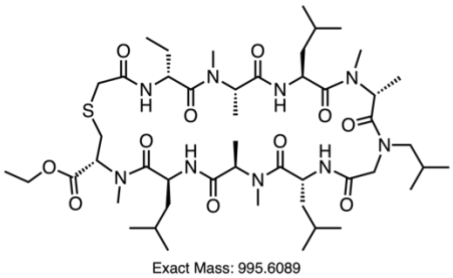

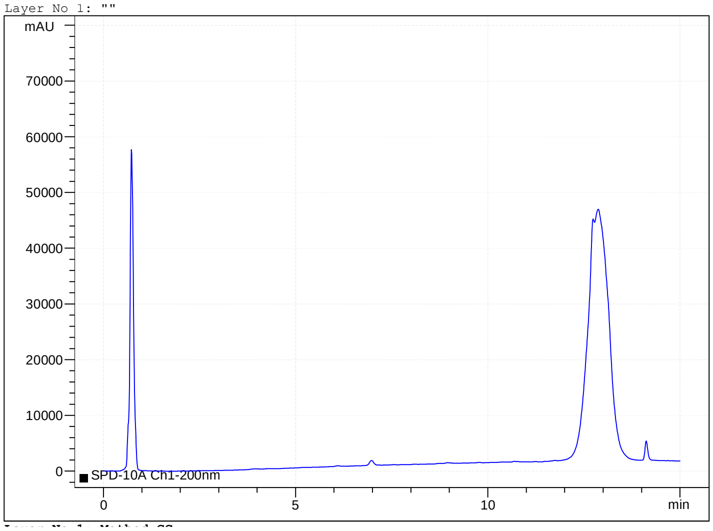

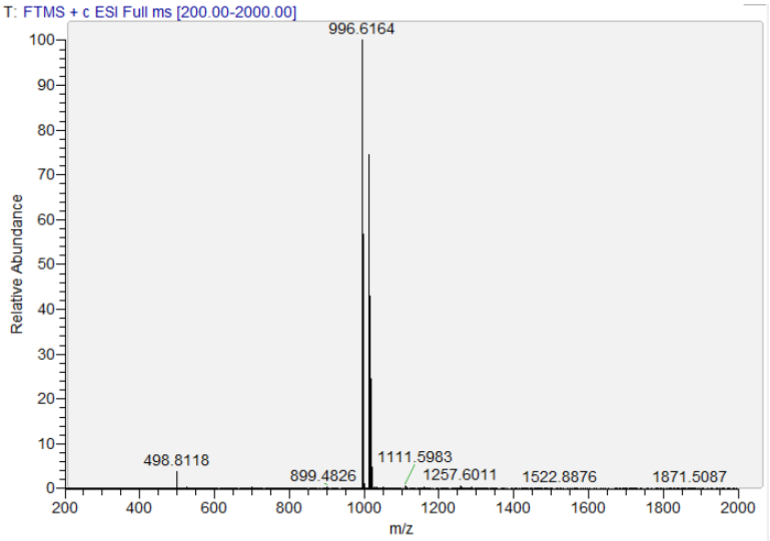

^{1}H NMR ($CDCl_3$)

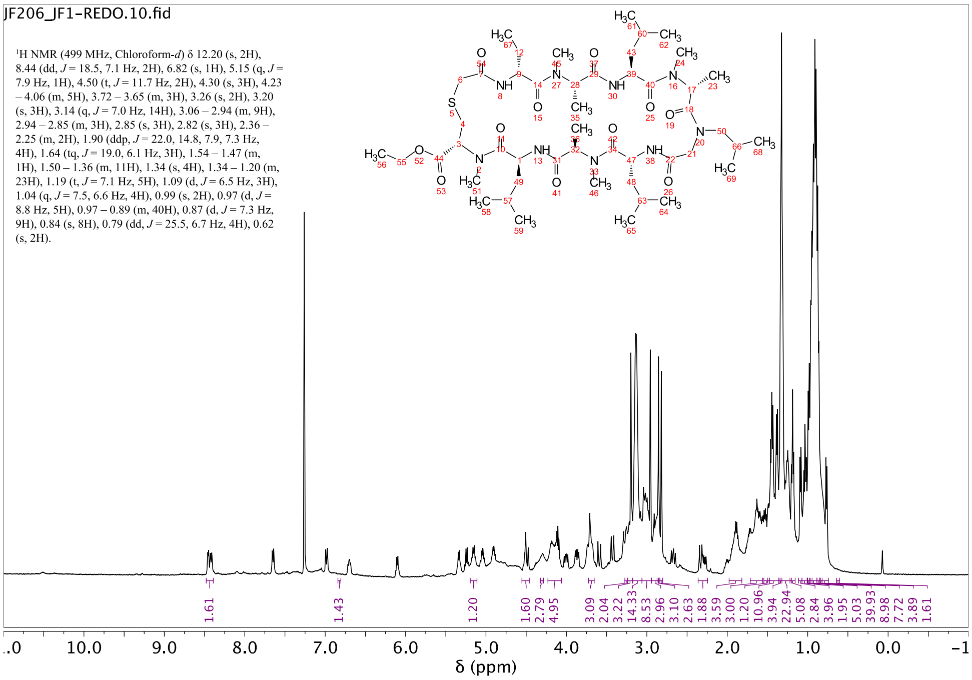

A1-1L

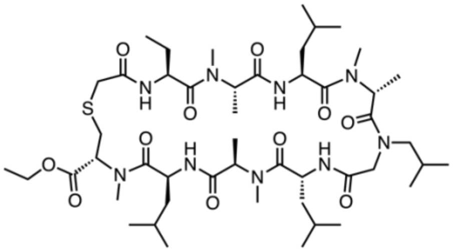

Exact Mass: 995.6089
Molecular Weight: 996.3200

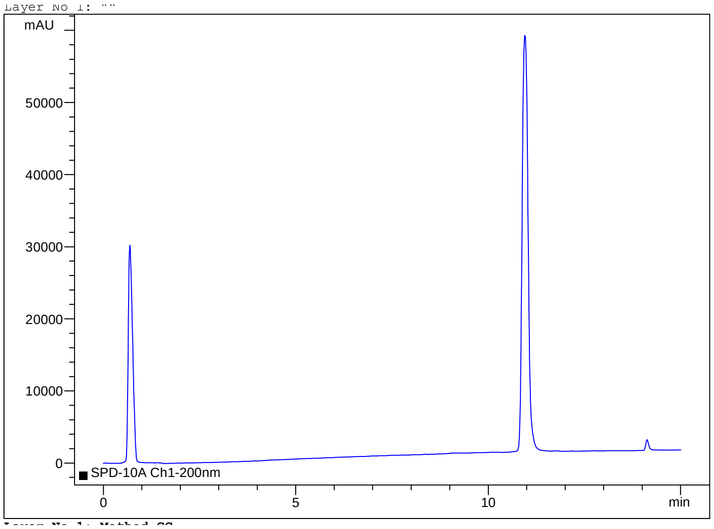

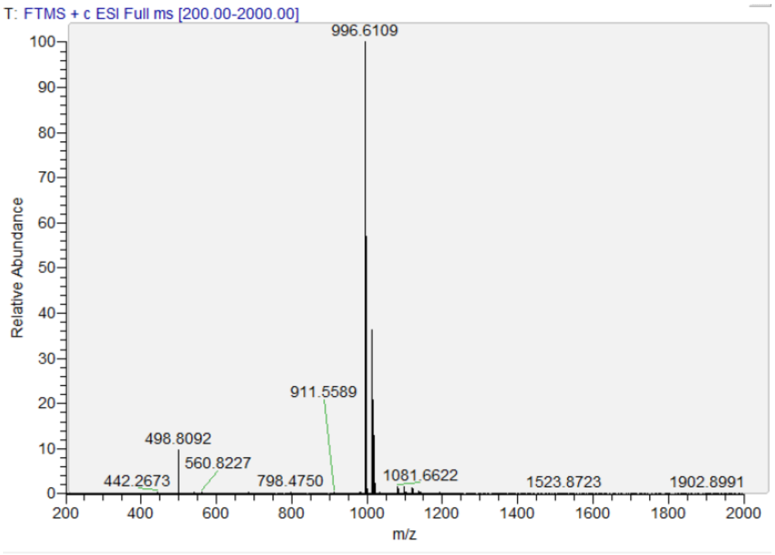
T: FTMS + c ESI Full ms [200.00-2000.00]
996.6109
911.5589
498.8092
560.8227
442.2673
798.4750
1081.6622
1523.8723
1902.8991
Relative Abundance
m/z

^1H NMR ($CDCl_3$)

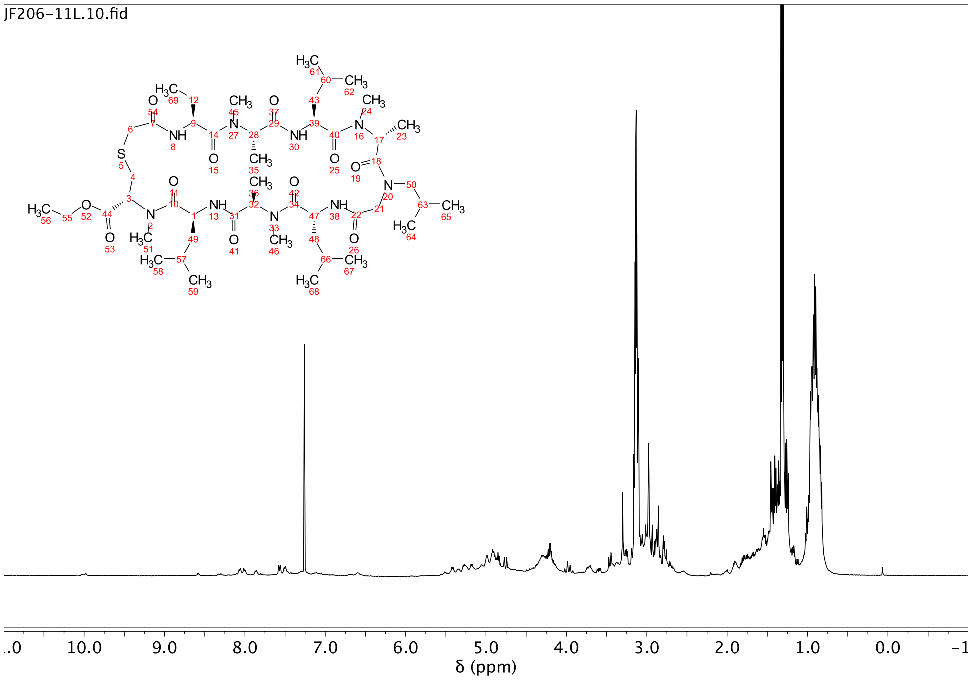
JF206-11L.10.fid
δ (ppm)

A1-3D

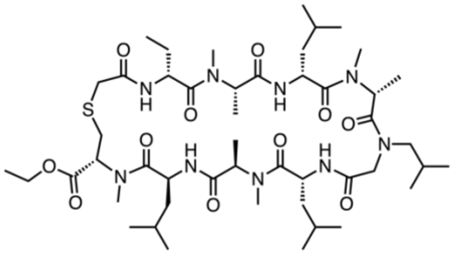

Exact Mass: 995.6089
Molecular Weight: 996.3200

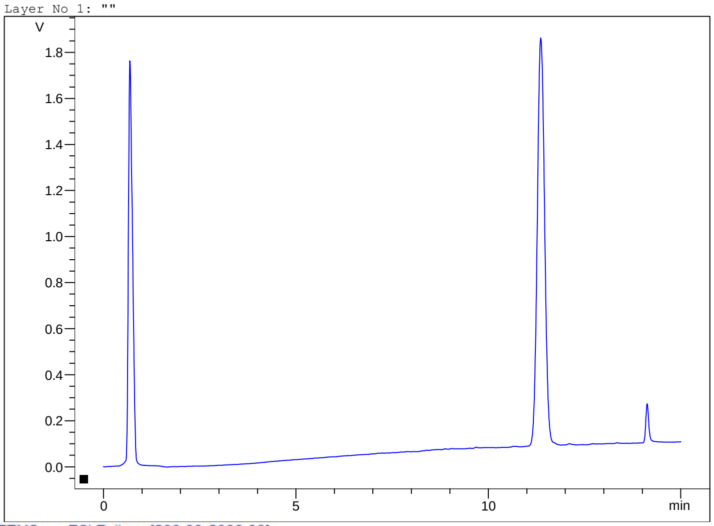

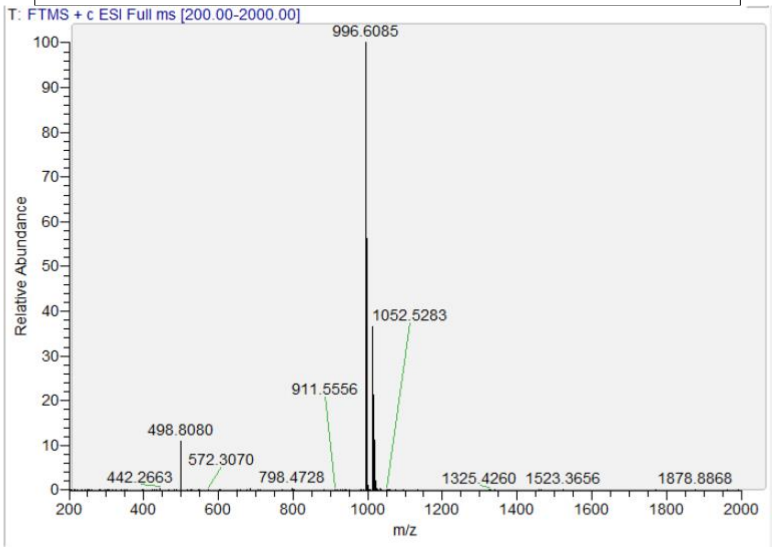

^{1}H NMR ($CDCl_3$)

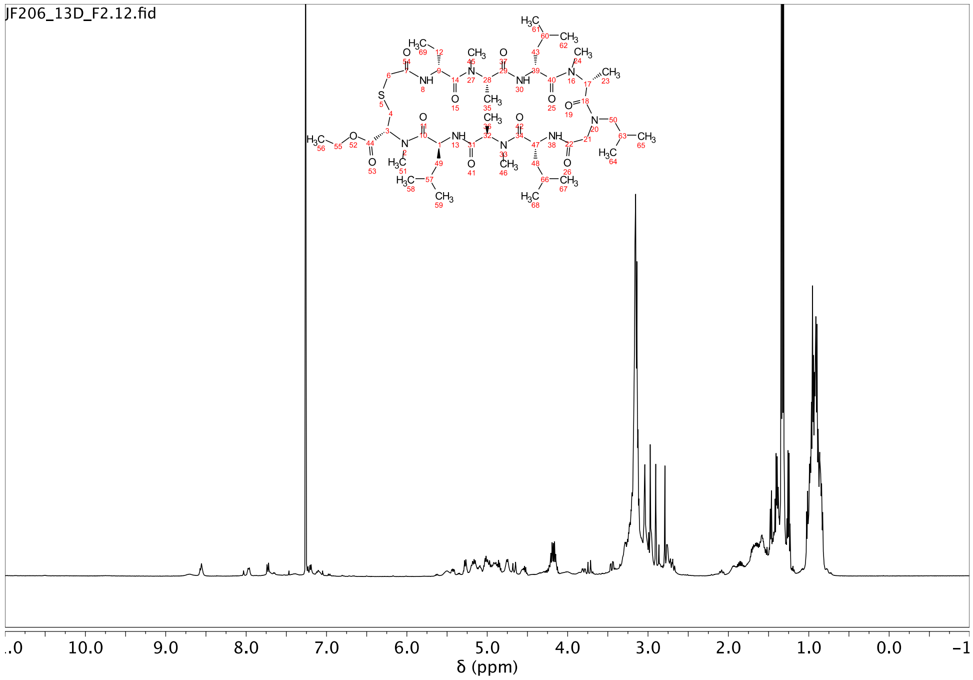

A1-4Dpro

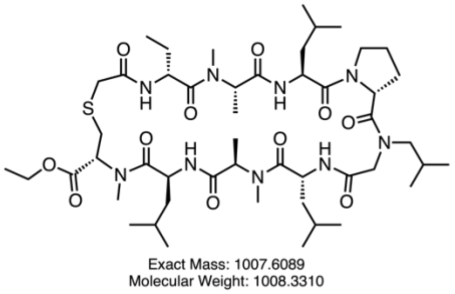

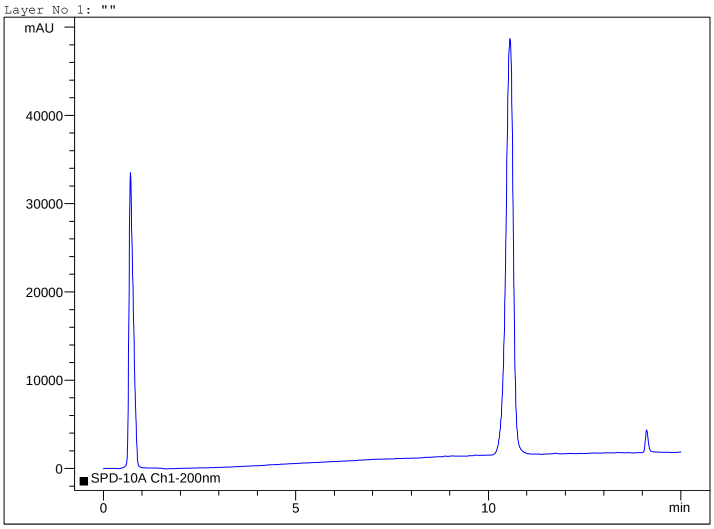

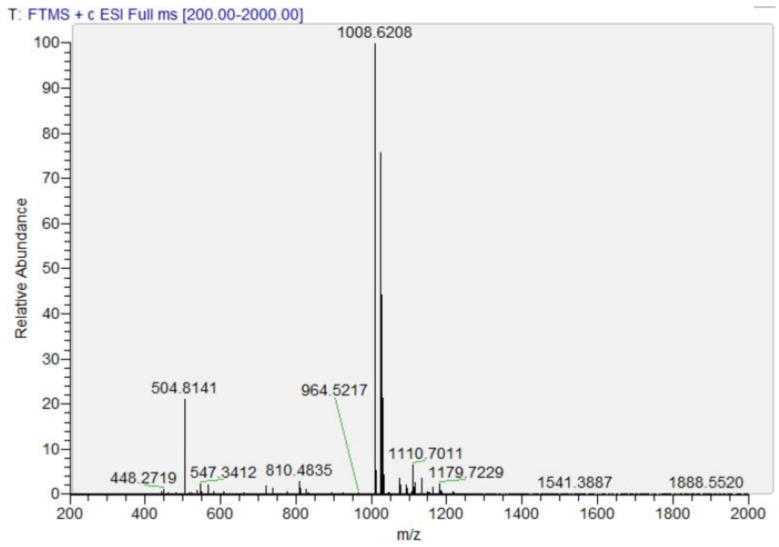
T: FTMS + c ESI Full ms [200.00-2000.00]
1008.6208
504.8141
964.5217
1110.7011
1179.7229
448.2719
547.3412
810.4835
1541.3887
1888.5520
Relative Abundance
m/z

^{1}H NMR ($CDCl_3$)

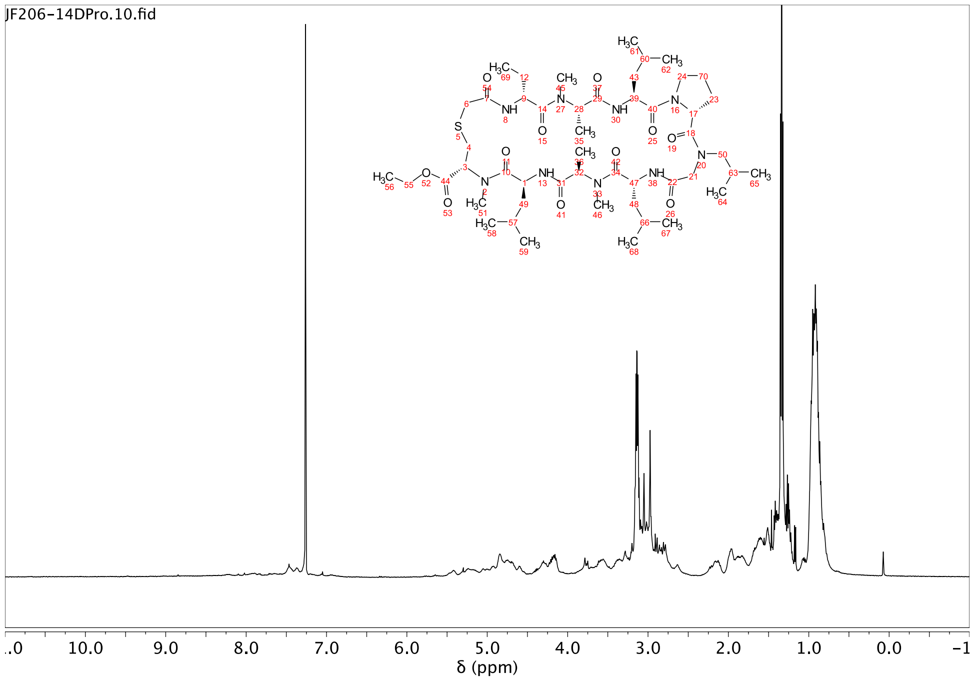
JF206-14DPro.10.fid
δ (ppm)

A1-6L

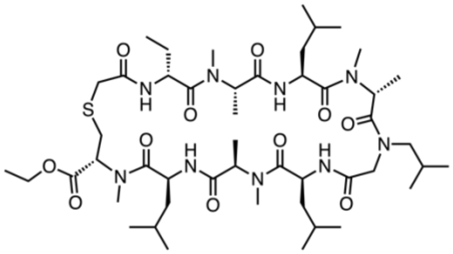
Exact Mass: 995.6089
Molecular Weight: 996.3200

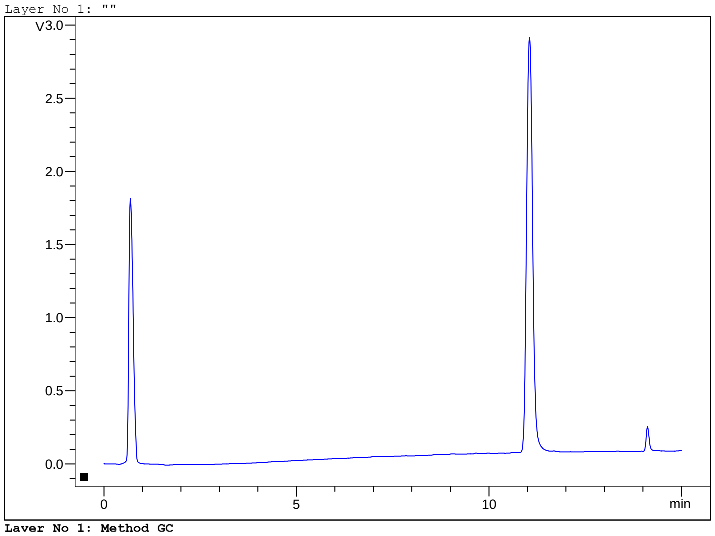
Layer No 1: ""
V
3.0
2.5
2.0
1.5
1.0
0.5
0.0
0
5
10
min
Layer No 1: Method GC

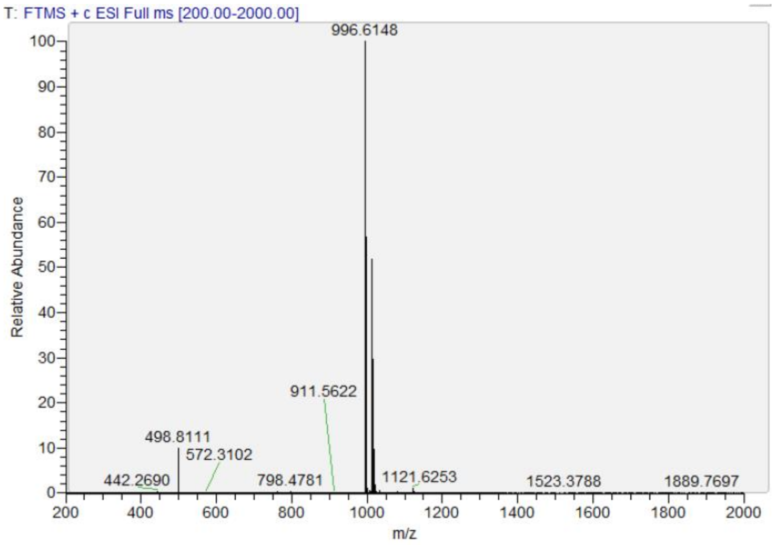
T: FTMS + c ESI Full ms [200.00-2000.00]
996.6148
911.5622
498.8111
572.3102
442.2690
798.4781
1121.6253
1523.3788
1889.7697
Relative Abundance
m/z

^{1}H NMR ($CDCl_3$)

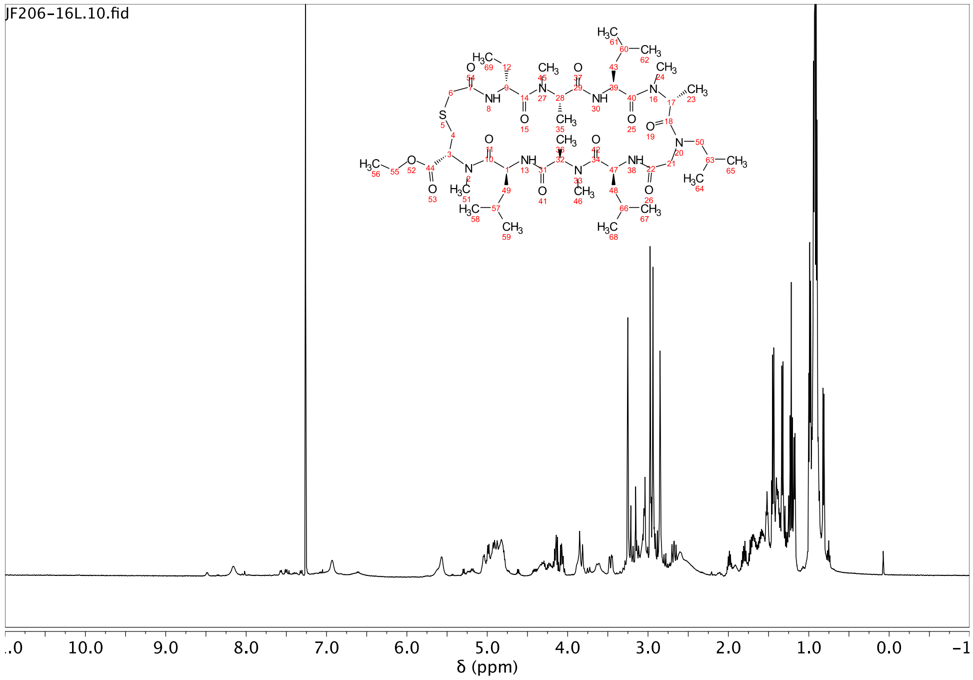

A1-7L

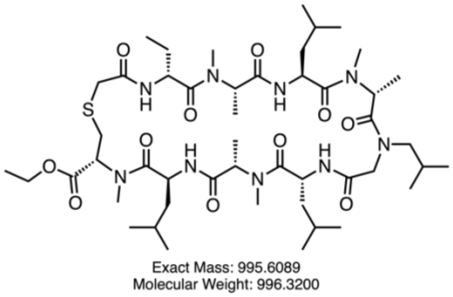

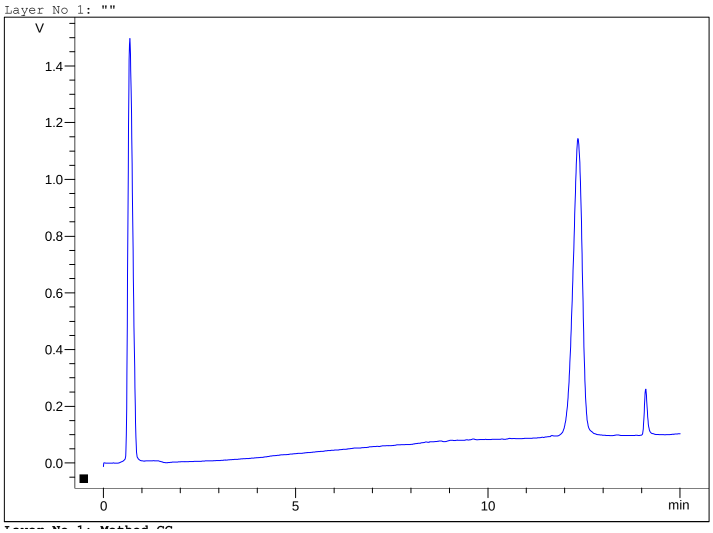

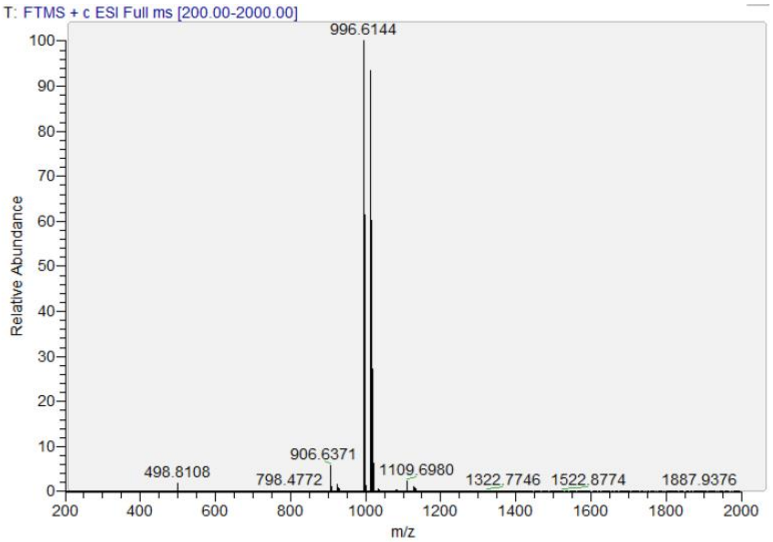
T: FTMS + c ESI Full ms [200.00-2000.00]
996.6144
906.6371
498.8108
798.4772
1109.6980
1322.7746
1522.8774
1887.9376
Relative Abundance
m/z

^{1}H NMR ($CDCl_3$)

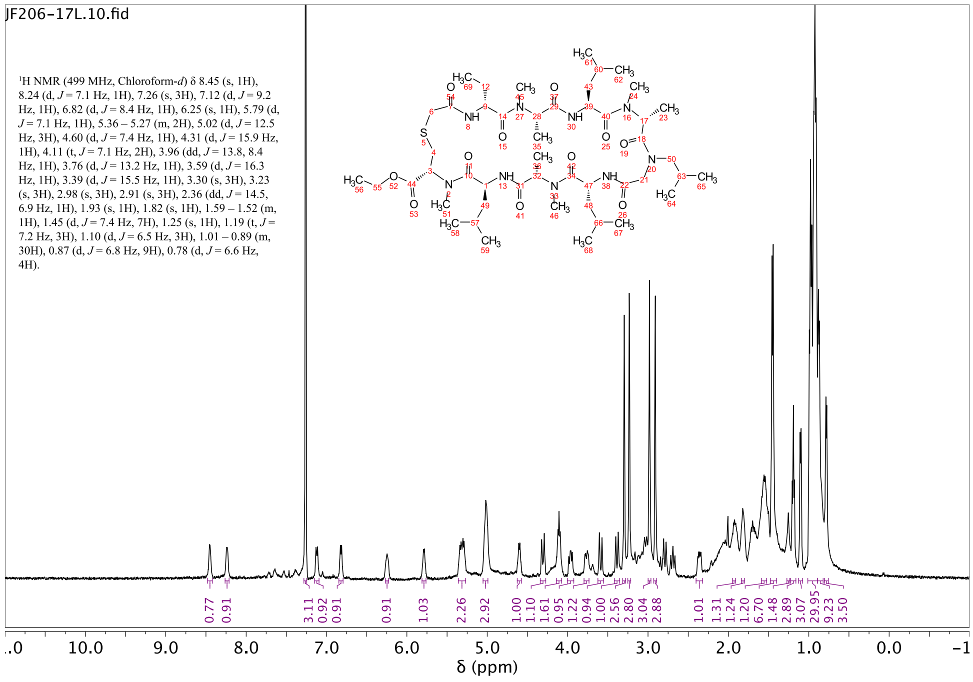
JF206-17L.10.fid
1H NMR (499 MHz, Chloroform-d) δ 8.45 (s, 1H), 8.24 (d, J = 7.1 Hz, 1H), 7.26 (s, 3H), 7.12 (d, J = 9.2 Hz, 1H), 6.82 (d, J = 8.4 Hz, 1H), 6.25 (s, 1H), 5.79 (d, J = 7.1 Hz, 1H), 5.36 – 5.27 (m, 2H), 5.02 (d, J = 12.5 Hz, 3H), 4.60 (d, J = 7.4 Hz, 1H), 4.31 (d, J = 15.9 Hz, 1H), 4.11 (t, J = 7.1 Hz, 2H), 3.96 (dd, J = 13.8, 8.4 Hz, 1H), 3.76 (d, J = 13.2 Hz, 1H), 3.59 (d, J = 16.3 Hz, 1H), 3.39 (d, J = 15.5 Hz, 1H), 3.30 (s, 3H), 3.23 (s, 3H), 2.98 (s, 3H), 2.91 (s, 3H), 2.36 (dd, J = 14.5, 6.9 Hz, 1H), 1.93 (s, 1H), 1.82 (s, 1H), 1.59 – 1.52 (m, 1H), 1.45 (d, J = 7.4 Hz, 7H), 1.25 (s, 1H), 1.19 (t, J = 7.2 Hz, 3H), 1.10 (d, J = 6.5 Hz, 3H), 1.01 – 0.89 (m, 30H), 0.87 (d, J = 6.8 Hz, 9H), 0.78 (d, J = 6.6 Hz, 4H).
δ (ppm)

B1

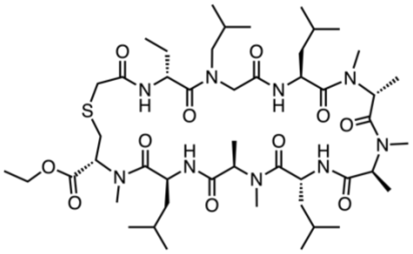
O
N
H
S
O
Exact Mass: 995.6089
Molecular Weight: 996.3200

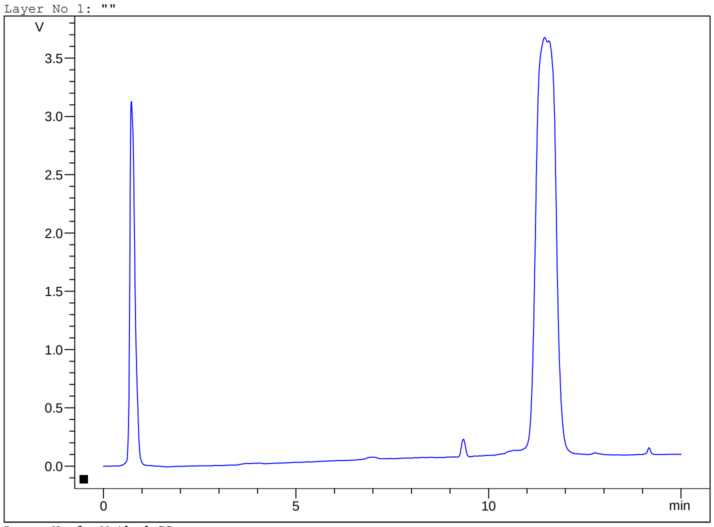
Layer No 1: ""
V
3.5
3.0
2.5
2.0
1.5
1.0
0.5
0.0
0
5
10
min

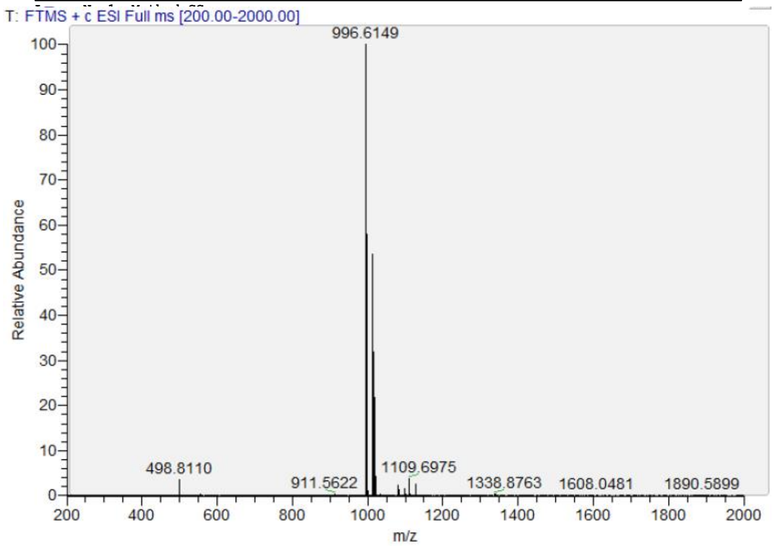
T: FTMS + c ESI Full ms [200.00-2000.00]
996.6149
Relative Abundance
498.8110
911.5622
1109.6975
1338.8763
1608.0481
1890.5899
m/z

^{1}H NMR ($CDCl_3$)

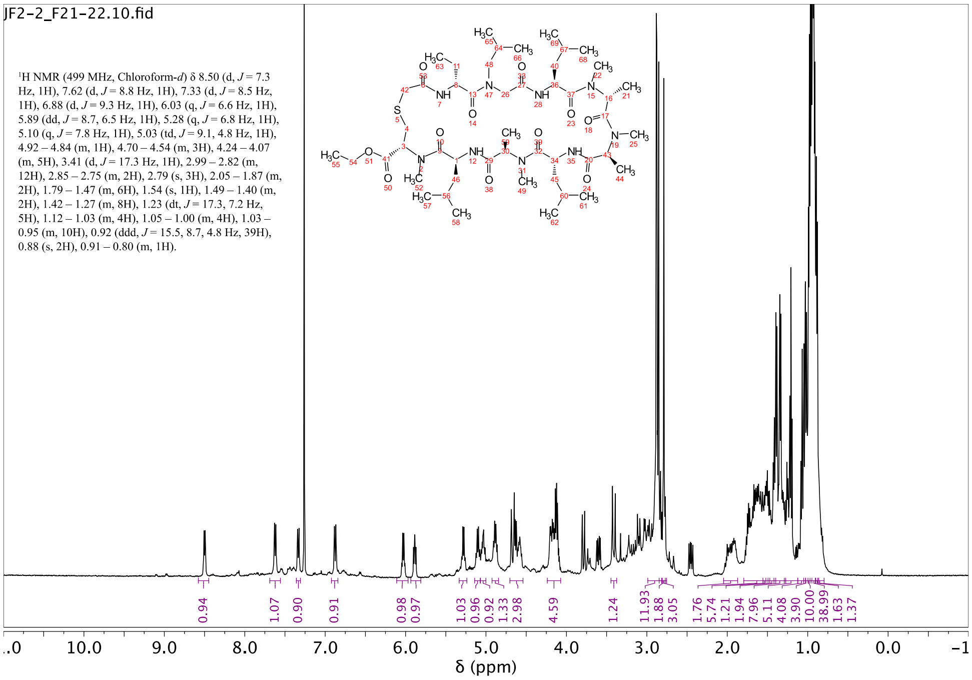

B1-1L

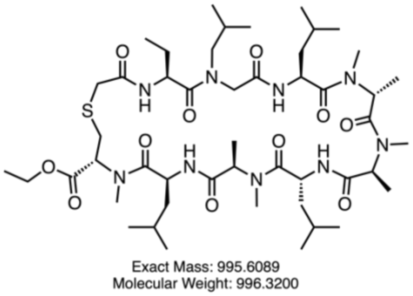

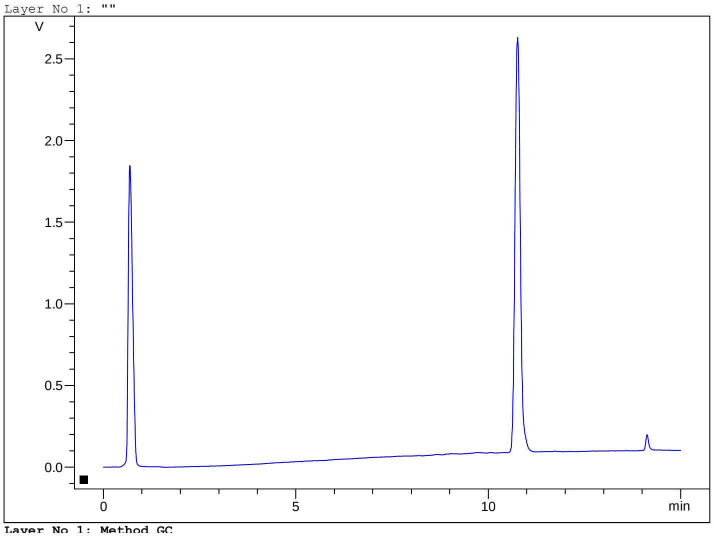

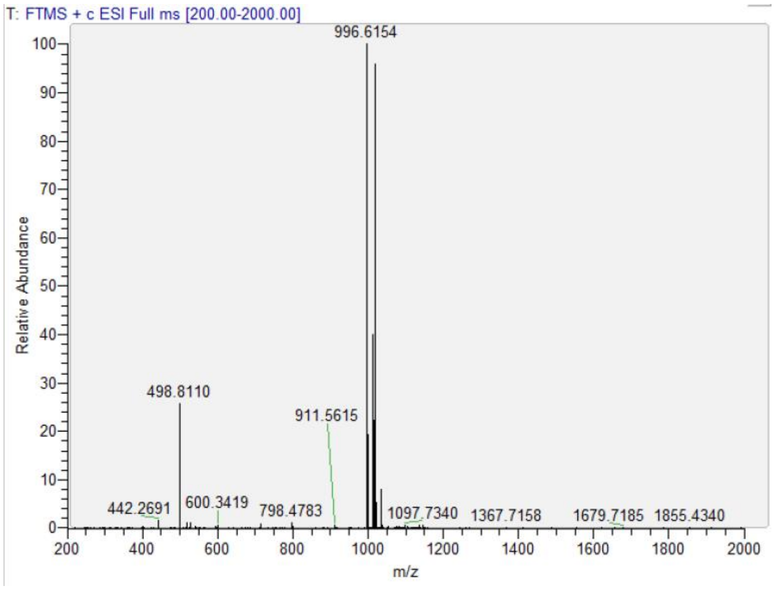

T: FTMS + c ESI Full ms [200.00-2000.00]
996.6154
498.8110
911.5615
442.2691
600.3419
798.4783
1097.7340
1367.7158
1679.7185
1855.4340
Relative Abundance
m/z

^{1}H NMR ($CDCl_3$)

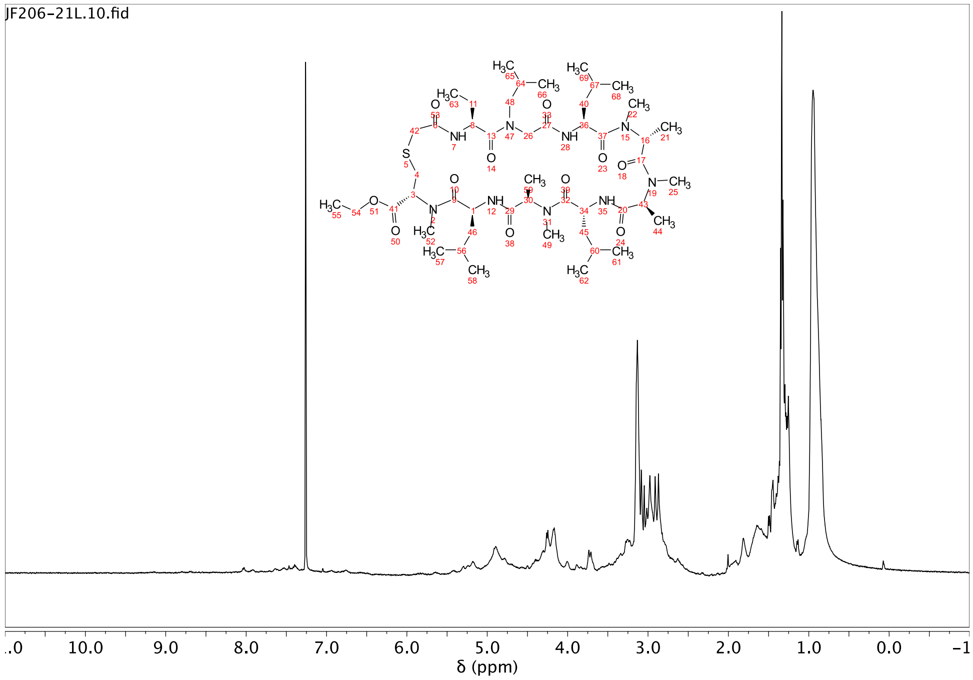

JF206-21L.10.fid
δ (ppm)

B1-3D

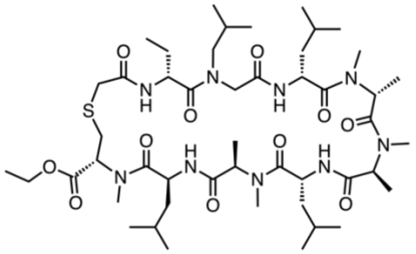

Exact Mass: 995.6089
Molecular Weight: 996.3200

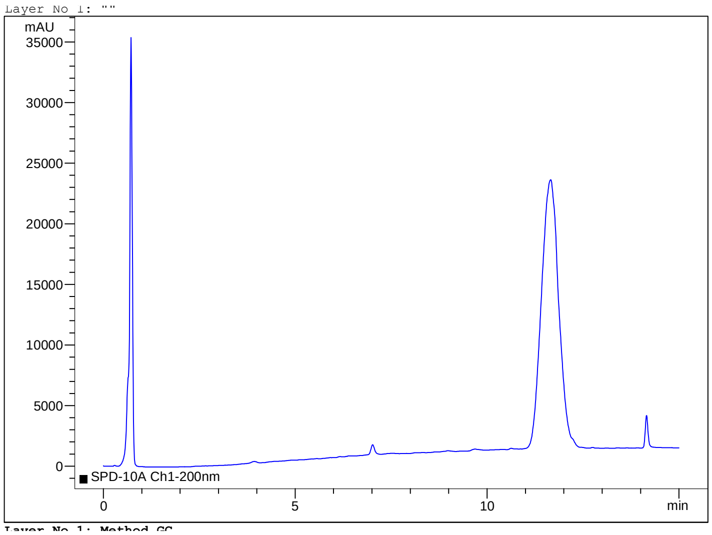

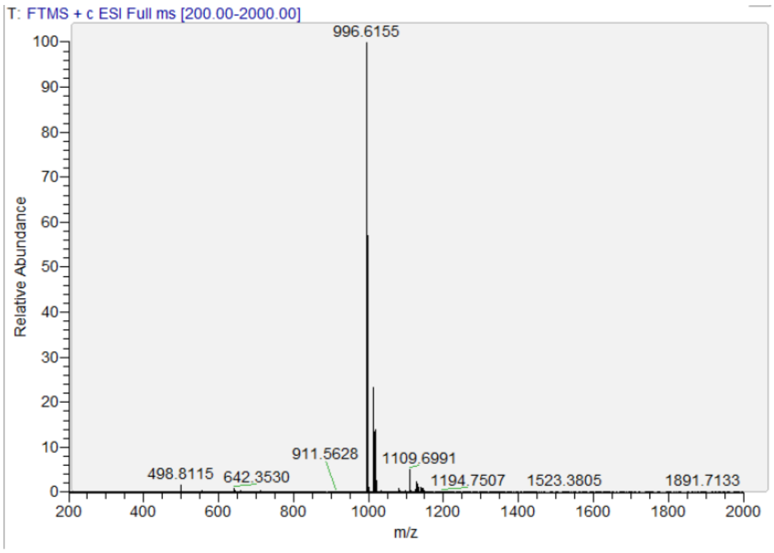

^{1}H NMR (CDCl$_3$)

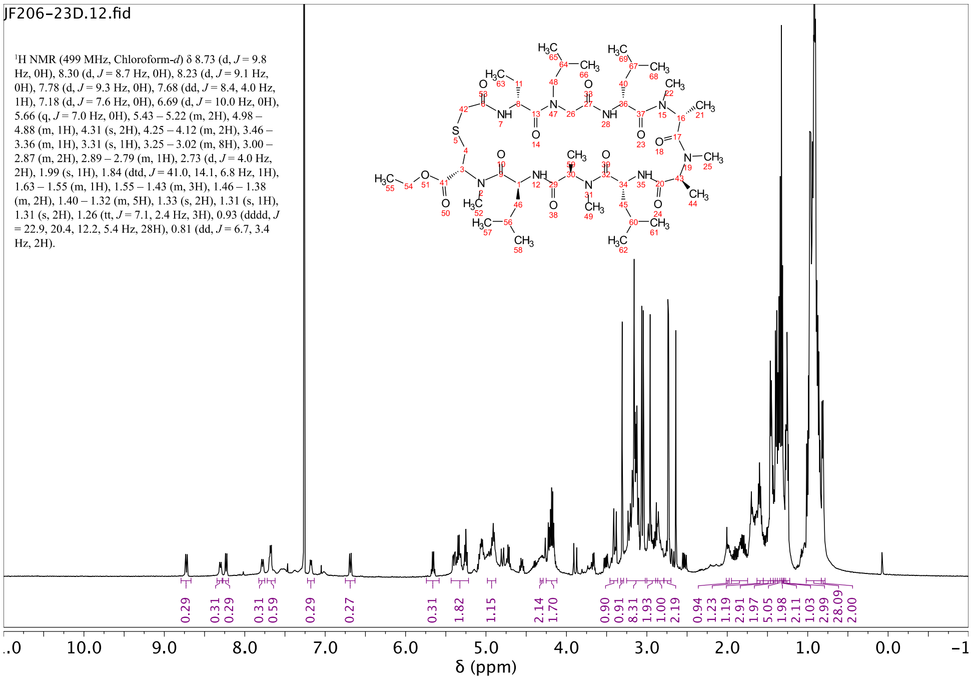

B1-4Dpro

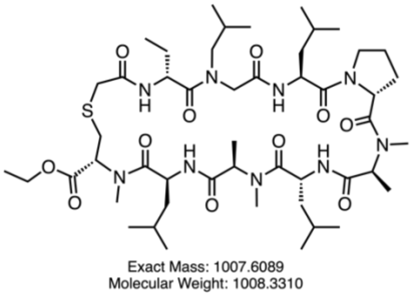

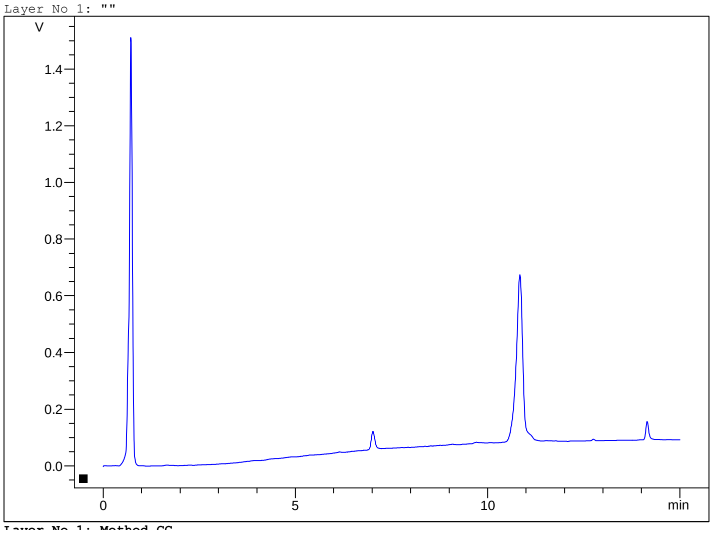

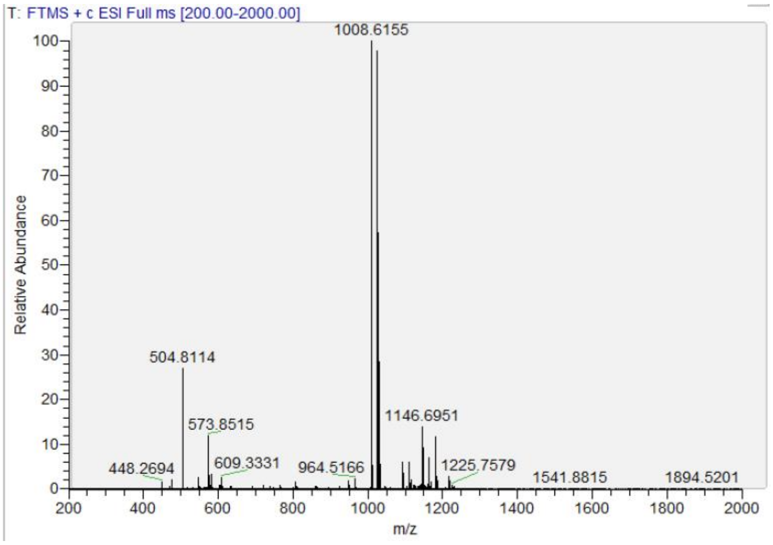
T: FTMS + c ESI Full ms [200.00-2000.00]
1008.6155
504.8114
573.8515
1146.6951
448.2694
609.3331
964.5166
1225.7579
1541.8815
1894.5201
Relative Abundance
m/z

^{1}H NMR ($CDCl_3$)

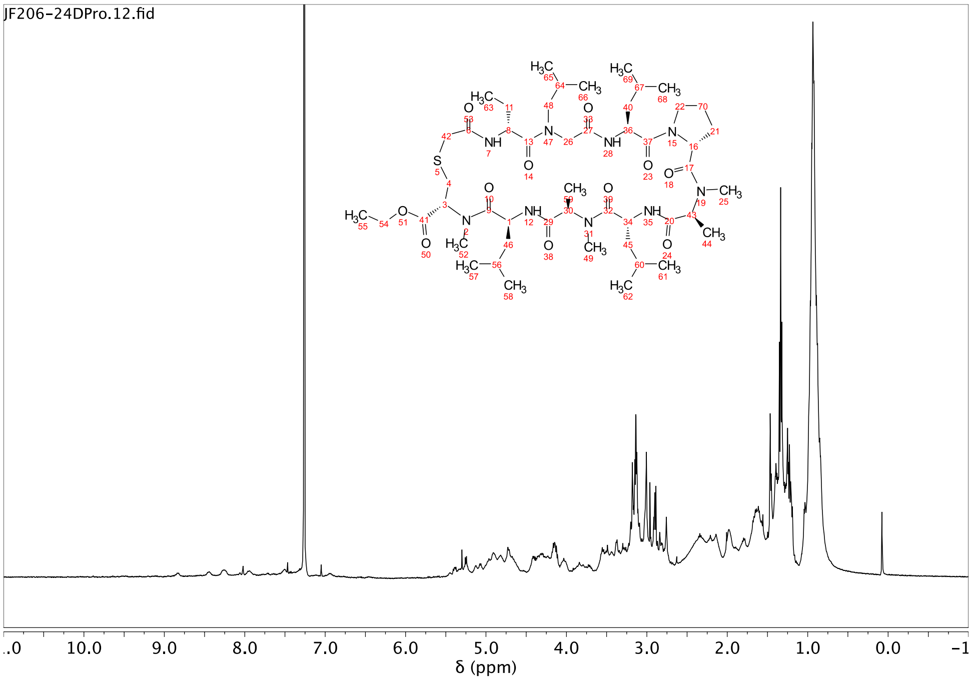
JF206-24DPro.12.fid
δ (ppm)

B1-6L

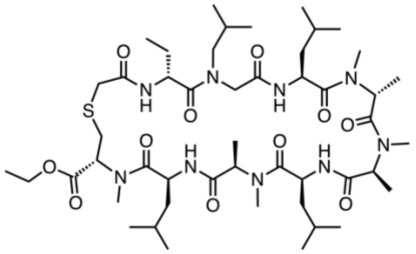
Exact Mass: 995.6089
Molecular Weight: 996.3200

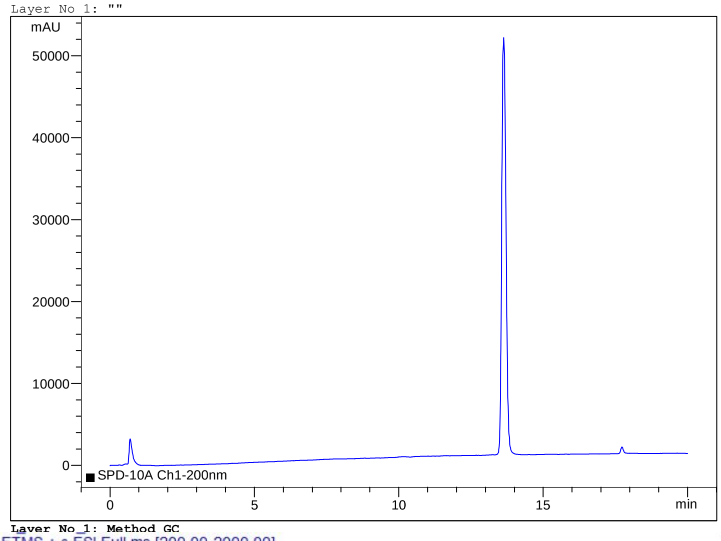
Layer No 1: ""
mAU
50000
40000
30000
20000
10000
0
SPD-10A Ch1-200nm
0
5
10
15
min
Layer No 1: Method GC

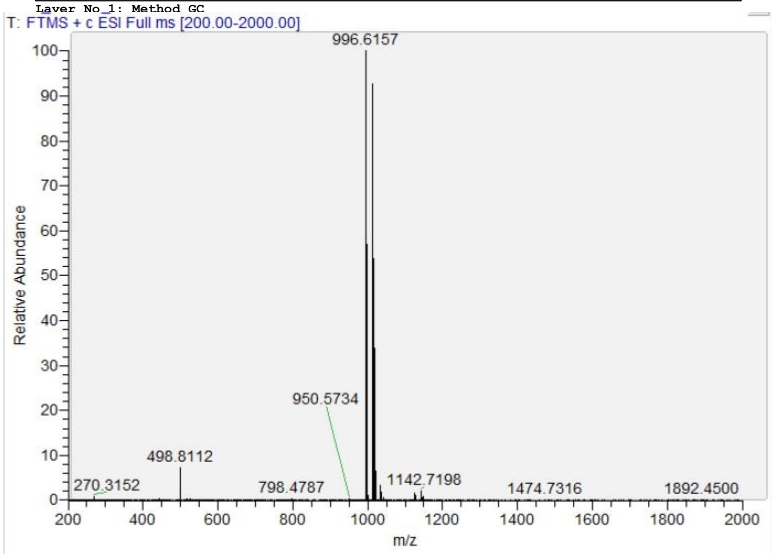
T: FTMS + c ESI Full ms [200.00-2000.00]
996.6157
950.5734
498.8112
270.3152
798.4787
1142.7198
1474.7316
1892.4500
Relative Abundance
m/z

^{1}H NMR (CDCl$_3$)

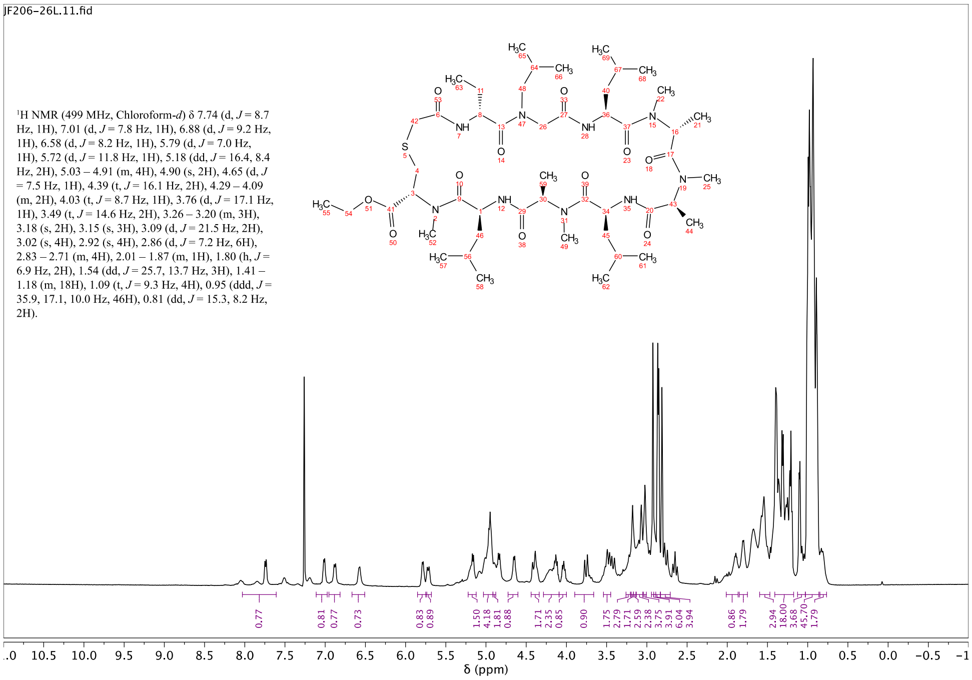

B1-7L

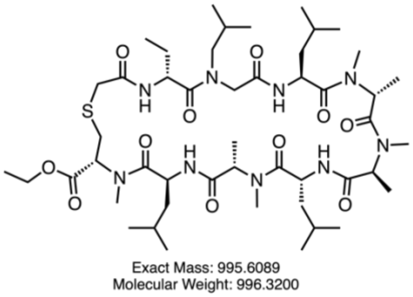

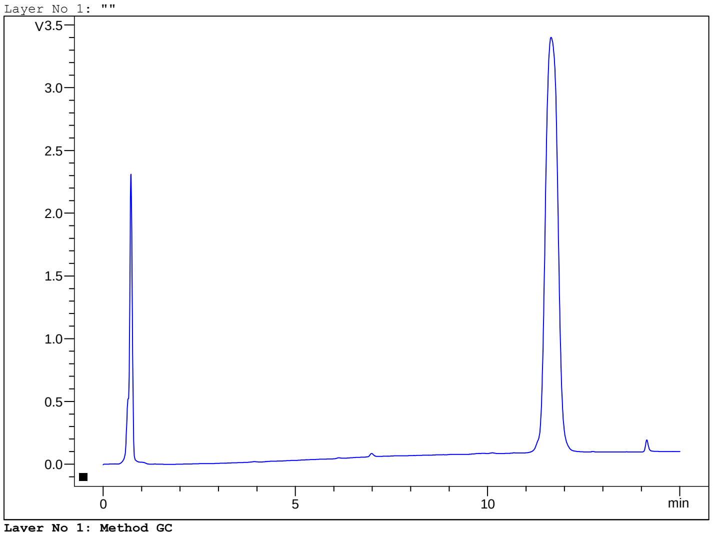

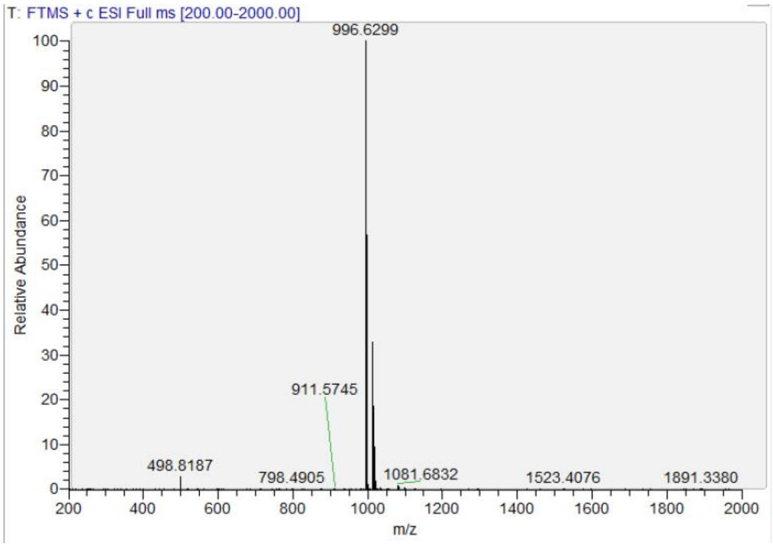
T: FTMS + c ESI Full ms [200.00-2000.00]
996.6299
911.5745
498.8187
798.4905
1081.6832
1523.4076
1891.3380
Relative Abundance
m/z

^{1}H NMR ($CDCl_3$)

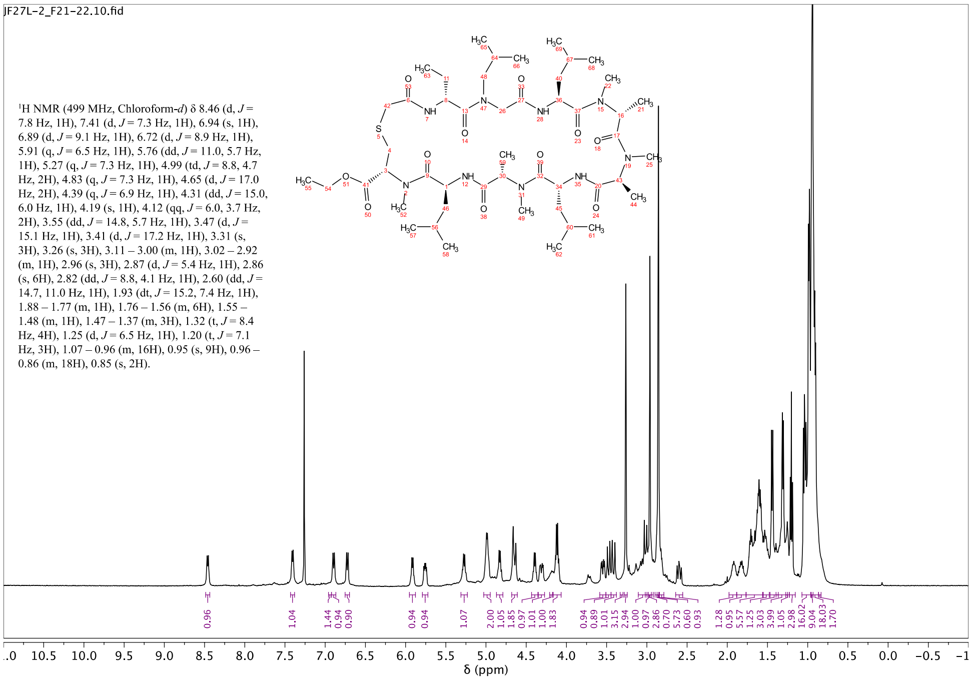
JF27L-2_F21-22.10.fid
1H NMR (499 MHz, Chloroform-d) δ 8.46 (d, J = 7.8 Hz, 1H), 7.41 (d, J = 7.3 Hz, 1H), 6.94 (s, 1H), 6.89 (d, J = 9.1 Hz, 1H), 6.72 (d, J = 8.9 Hz, 1H), 5.91 (q, J = 6.5 Hz, 1H), 5.76 (dd, J = 11.0, 5.7 Hz, 1H), 5.27 (q, J = 7.3 Hz, 1H), 4.99 (td, J = 8.8, 4.7 Hz, 2H), 4.83 (q, J = 7.3 Hz, 1H), 4.65 (d, J = 17.0 Hz, 2H), 4.39 (q, J = 6.9 Hz, 1H), 4.31 (dd, J = 15.0, 6.0 Hz, 1H), 4.19 (s, 1H), 4.12 (qq, J = 6.0, 3.7 Hz, 2H), 3.55 (dd, J = 14.8, 5.7 Hz, 1H), 3.47 (d, J = 15.1 Hz, 1H), 3.41 (d, J = 17.2 Hz, 1H), 3.31 (s, 3H), 3.26 (s, 3H), 3.11 – 3.00 (m, 1H), 3.02 – 2.92 (m, 1H), 2.96 (s, 3H), 2.87 (d, J = 5.4 Hz, 1H), 2.86 (s, 6H), 2.82 (dd, J = 8.8, 4.1 Hz, 1H), 2.60 (dd, J = 14.7, 11.0 Hz, 1H), 1.93 (dt, J = 15.2, 7.4 Hz, 1H), 1.88 – 1.77 (m, 1H), 1.76 – 1.56 (m, 6H), 1.55 – 1.48 (m, 1H), 1.47 – 1.37 (m, 3H), 1.32 (t, J = 8.4 Hz, 4H), 1.25 (d, J = 6.5 Hz, 1H), 1.20 (t, J = 7.1 Hz, 3H), 1.07 – 0.96 (m, 16H), 0.95 (s, 9H), 0.96 – 0.86 (m, 18H), 0.85 (s, 2H).
δ (ppm)

References

(1) Diaz-Rodriguez, V.; Ganusova, E.; Rappe, T. M.; Becker, J. M.; Distefano, M. D. Synthesis of Peptides Containing C-Terminal Esters Using Trityl Side-Chain Anchoring: Applications to the Synthesis of C-Terminal Ester Analogs of the Saccharomyces cerevisiae Mating Pheromone a-Factor. *J Org Chem* **2015**, *80* (22), 11266-11274. DOI: 10.1021/acs.joc.5b01376 From NLM Medline.
(2) Diaz-Rodriguez, V.; Mullen, D. G.; Ganusova, E.; Becker, J. M.; Distefano, M. D. Synthesis of peptides containing C-terminal methyl esters using trityl side-chain anchoring: application to the synthesis of a-factor and a-factor analogs. *Org Lett* **2012**, *14* (22), 5648-5651. DOI: 10.1021/ol302592v From NLM Medline.
(3) Freidinger, R. M.; Hinkle, J. S.; Perlow, D. S.; Arison, B. H. Synthesis of 9-Fluorenylmethyloxycarbonyl-Protected N-Alkyl Amino-Acids by Reduction of Oxazolidinones. *Journal of Organic Chemistry* **1983**, *48* (1), 77-81. DOI: DOI 10.1021/jo00149a016.
(4) Naylor, M. R.; Ly, A. M.; Handford, M. J.; Ramos, D. P.; Pye, C. R.; Furukawa, A.; Klein, V. G.; Noland, R. P.; Edmondson, Q.; Turmon, A. C.; et al. Lipophilic Permeability Efficiency Reconciles the Opposing Roles of Lipophilicity in Membrane Permeability and Aqueous Solubility. *J Med Chem* **2018**, *61* (24), 11169-11182. DOI: 10.1021/acs.jmedchem.8b01259 From NLM Medline.
(5) Hewitt, W. M.; Leung, S. S.; Pye, C. R.; Ponkey, A. R.; Bednarek, M.; Jacobson, M. P.; Lokey, R. S. Cell-permeable cyclic peptides from synthetic libraries inspired by natural products. *J Am Chem Soc* **2015**, *137* (2), 715-721. DOI: 10.1021/ja508766b From NLM Medline.
(6) *Molecular Operating Environment (MOE)*; Chemical Computing Group ULC: 1010 Sherbooke St. West, Suite #910, Montreal, QC, Canada H3A 2R7, 2018. (accessed.
(7) Martinez, L.; Andrade, R.; Birgin, E. G.; Martinez, J. M. PACKMOL: a package for building initial configurations for molecular dynamics simulations. *J Comput Chem* **2009**, *30* (13), 2157-2164. DOI: 10.1002/jcc.21224 From NLM PubMed-not-MEDLINE.
(8) Duan, Y.; Wu, C.; Chowdhury, S.; Lee, M. C.; Xiong, G.; Zhang, W.; Yang, R.; Cieplak, P.; Luo, R.; Lee, T.; et al. A point-charge force field for molecular mechanics simulations of proteins based on condensed-phase quantum mechanical calculations. *J Comput Chem* **2003**, *24* (16), 1999-2012. DOI: 10.1002/jcc.10349 From NLM Medline.
(9) Khoury, G. A.; Smadbeck, J.; Tamamis, P.; Vandris, A. C.; Kieslich, C. A.; Floudas, C. A. Forcefield_NCAA: ab initio charge parameters to aid in the discovery and design of therapeutic proteins and peptides with unnatural amino acids and their application to complement inhibitors of the compstatin family. *ACS Synth Biol* **2014**, *3* (12), 855-869. DOI: 10.1021/sb400168u From NLM Medline.
(10) Wang, J.; Wolf, R. M.; Caldwell, J. W.; Kollman, P. A.; Case, D. A. Development and testing of a general amber force field. *J Comput Chem* **2004**, *25* (9), 1157-1174. DOI: 10.1002/jcc.20035 From NLM Medline.
(11) Dupradeau, F. Y.; Pigache, A.; Zaffran, T.; Savineau, C.; Lelong, R.; Grivel, N.; Lelong, D.; Rosanski, W.; Cieplak, P. The R.E.D. tools: advances in RESP and ESP charge derivation and force field library building. *Phys Chem Chem Phys* **2010**, *12* (28), 7821-7839. DOI: 10.1039/c0cp00111b From NLM Medline.
(12) Higo, J.; Umezawa, K.; Nakamura, H. A virtual-system coupled multicanonical molecular dynamics simulation: Principles and applications to free-energy landscape of protein-protein interaction with an all-atom model in explicit solvent. *J Chem Phys* **2013**, *138* (18). DOI: Artn 184106
10.1063/1.4803468.
(13) Higo, J.; Kamiya, N.; Sugihara, T.; Yonezawa, Y.; Nakamura, H. Verifying trivial parallelization of multicanonical molecular dynamics for conformational sampling of a polypeptide in explicit water. *Chem Phys Lett* **2009**, *473* (4-6), 326-329. DOI: 10.1016/j.cplett.2009.03.077.
(14) Bussi, G.; Donadio, D.; Parrinello, M. Canonical sampling through velocity rescaling. *J Chem Phys* **2007**, *126* (1). DOI: Artn 014101
10.1063/1.2408420.
(15) Guntert, P. Automated NMR structure calculation with CYANA. *Methods Mol Biol* **2004**, *278*, 353-378. DOI: 10.1385/1-59259-809-9:353 From NLM Medline.
(16) Yilmaz, E. M.; Guntert, P. NMR structure calculation for all small molecule ligands and non-standard residues from the PDB Chemical Component Dictionary. *J Biomol NMR* **2015**, *63* (1), 21-37. DOI: 10.1007/s10858-015-9959-y From NLM Medline.
